# High-performance red light-emitting diodes from quasi-two-dimensional perovskite nanocrystals

**DOI:** 10.1038/s41467-026-72733-w

**Published:** 2026-05-07

**Authors:** Jibin Zhang, Tianjun Liu, Qichun Gu, Shuailing Lin, Jian Mao, Zimu Wei, Meng Wang, Yang Lu, Bo Cai, Zher Ying Ooi, Alessandro James Mirabelli, Linyuan Lian, Miguel Anaya, Ying Liu, Mochen Jia, Xu Chen, Yanbing Han, Xinzhen Ji, Yiwei Zhang, Zhuangzhuang Ma, Xinping Zhang, Xin Zhou, Xinjian Li, Fanglong Yuan, Lintao Hou, Chongxin Shan, Neil C. Greenham, Samuel D. Stranks, Zhifeng Shi

**Affiliations:** 1 Key Laboratory of Materials Physics of Ministry of Education, School of Physics, Zhengzhou University, Zhengzhou, China; 2 Faculty of Informatics and Engineering, The University of Electro-Communications, Chofu, Tokyo Japan; 3 Cavendish Laboratory, University of Cambridge, Cambridge, UK; 4 Department of Chemical Engineering and Biotechnology, University of Cambridge, Cambridge, UK; 5 State Key Laboratory of Photovoltaic Science and Technology, Shanghai Frontiers Science Research Base of Intelligent Optoelectronics and Perception, Institute of Optoelectronics, Fudan University, Shanghai, China; 6 Department of Materials Science and Metallurgy, University of Cambridge, Cambridge, UK; 7 Departamento Física de la Materia Condensada, Instituto de Ciencia de Materiales de Sevilla, Universidad de Sevilla-CSIC, Sevilla, Spain; 8 Institute of Information Photonics Technology, Beijing University of Technology, Beijing, China; 9 Guangdong Provincial Key Laboratory of Semiconductor Micro Display, Foshan Nationstar Optoelectronics Company Ltd., Foshan, China; 10 Key Laboratory of Theoretical & Computational Photochemistry of Ministry of Education, College of Chemistry, Beijing Normal University, Beijing, China; 11 Guangzhou Key Laboratory of Vacuum Coating Technologies and New Energy Materials, College of Physics and Optical Engineering, Jinan University, Guangzhou, China

**Keywords:** Inorganic LEDs, Inorganic LEDs

## Abstract

Metal halide perovskite light-emitting diodes offer a promising platform for low-cost full-color displays, yet achieving high-performance pure-red emission remains challenging. Here, we report a crystallization regulation strategy for mixed bromide/iodide quasi-two-dimensional perovskites using a multifunctional molecule, 4-(trifluoromethyl)benzenesulfonamide, which simultaneously coordinates with organic spacer cations, Pb^2+^ ions and halide ions. Moreover, the combination of large steric hindrance and ordered molecular assembly in the precursor solution plays a decisive role in directing the formation of nanocrystals, thereby suppressing defect formation, inhibiting halide ions migration, and enhancing exciton binding energy. The resulting light-emitting diodes exhibited pure-red emission at ~635 nm, delivering a peak external quantum efficiency of 30.2%, a maximum luminance exceeding 25,000 cd m^-2^, and a half-lifetime of 8426 min. Achieving perovskite light-emitting diodes with performance comparable to that of quantum-dot or organic light-emitting diodes would mark a major milestone toward commercialization. This work would expand opportunities beyond conventional light-emitting diode technologies.

## Abstract (web summary)

Zhang et al. report a multifunctional molecule to control the growth of quasi-2D perovskite nanocrystals, reducing defects and ion migration. This enables pure-red LEDs with an efficiency of 30.2%, operational stability of 8426 min, and 12.25 cm^2^ devices maintaining above 20% efficiencies.

## Introduction

For next-generation high-resolution displays, metal halide perovskites light-emitting diodes (PeLEDs) are particularly appealing because they combine facile spectral tunability, high color saturation and low-temperature solution processability^1–5^. With continued advances in light extraction and defect suppression, PeLEDs have now reached external quantum efficiencies (EQEs) exceeding 30%^6–8^, narrowing the performance gap with established QLED and OLED technologies^9,10^. Pure-red PeLEDs emitting at ~635 nm is desired to fulfill the color gamut specified by Rec. 2020 display standard, but the realization of pure-red PeLEDs with both high luminance and high efficiency remains challenging. Recently, perovskite nanocrystals synthesized in colloidal form have been identified as promising light emitters for high-performance pure-red PeLEDs^11–16^. These nanocrystals exhibit high photoluminescence quantum efficiency (PLQE) due to the carrier confinement properties^17,18^. Moreover, one-step solution-processed quasi-2D perovskites exhibit great application potential in PeLEDs. Typically, the quasi-2D perovskites consist of lead halide octahedra (which constitute quantum wells with different bandgaps) separated by bilayers of organic spacer cations^19–21^. The distinctive energy funnel or cascade structure of quasi-2D perovskites facilitates rapid and efficient transfer of photocarriers from higher bandgap to lower bandgap sites, which favor for enhanced charge-transport characteristics and radiative recombination efficiency compared with 3D perovskites^22,23^. Given the advantages of nanocrystals and quasi-2D perovskites, leveraging nanocrystals based on quasi-2D perovskites, which combine efficient carrier transfer, recombination properties, and enhanced light out-coupling efficiency, presents a promising pathway for developing high-performance pure-red PeLEDs.

**Fig. 1:**
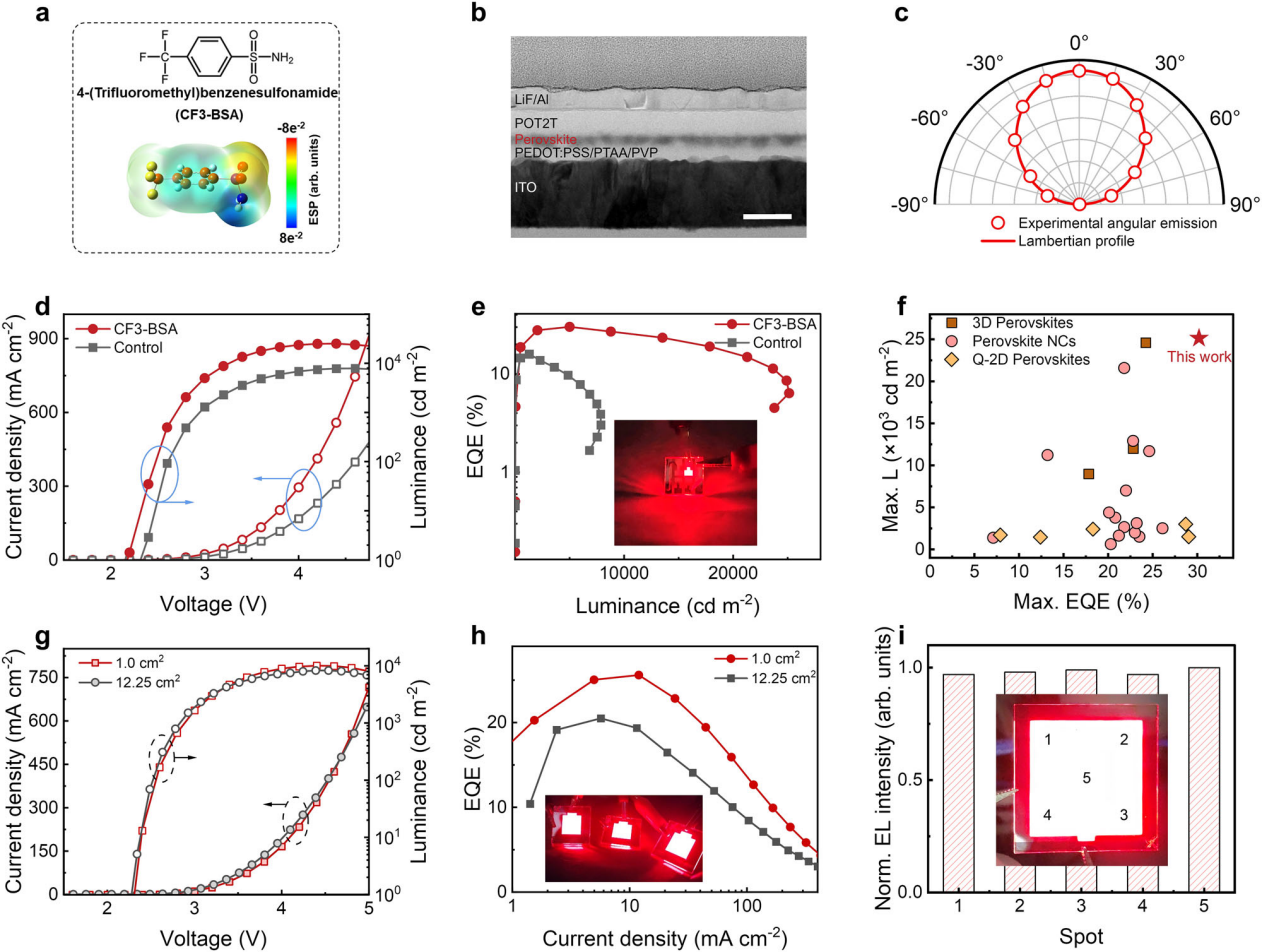
Performance of the pure-red PeLEDs. **a** Molecular structure and calculated ESP of CF3-BSA. **b** Cross-sectional transmission electron microscopy (TEM) image of the device (scale bar: 100 nm). **c** Angle-dependent EL intensity of PeLEDs. **d** Current density versus voltage and luminance versus voltage characteristics of the champion control and CF3-BSA based PeLEDs. **e** EQE versus luminance characteristics of the devices. Inset: photograph of a working device with 0.07 cm^2^ active area. **f** Reported peak EQE and peak luminance of PeLEDs in the pure-red regime (630–650 nm) based on 3D perovskites, quasi-2D perovskites, and colloidal perovskite nanocrystals according to the data provided in Supplementary Table 1. **g** Current density versus voltage and luminance versus voltage characteristics of the large area devices. **h** EQE versus current density curves of the large area devices. Inset: photograph of three working devices with 1.0 cm^2^ active area. **i** EL uniformity statistics of the large-area PeLED with an emitting area of 12.25 cm^2^.

In this work, we employ a multifunctional molecule of 4-(trifluoromethyl)benzenesulfonamide (CF3-BSA, Fig. 1a), featuring large steric hindrance and ordered molecular assembly in precursor solutions, to regulate the crystallization of quasi-2D perovskite films through simultaneous interactions with organic spacer cations, Pb^2+^ ions, and halide ions. This approach enables effective manipulation of the optoelectronic, crystalline, and morphological properties of mixed-halide quasi-2D perovskites with nanocrystal structures. The resulting quasi-2D perovskite nanocrystals exhibit low density of defects, homogenous phase distribution, suppressed halide ion migration, and increased exciton binding energy. As a result, these high-quality perovskite emitters enable pure-red PeLEDs with a peak EQE of 30.2% and a maximum luminance exceeding 25,000 cd m^−2^. Impressively, they demonstrate an operational half-lifetime of 8426 min at an initial luminance of 100 cd m^−2^, setting a benchmark for the stability of pure-red PeLEDs. Moreover, this strategy also yields large-area devices with peak EQEs of 25.6% for 1.0 cm^2^ and 20.5% for 12.25 cm^2^. This achievement represents a high level of performance among pure-red PeLEDs, rivaling the state-of-the-art red OLEDs and QLEDs^24,25^.

## Results and discussion

### Performance of the devices

The quasi-2D perovskite films were deposited through a simple one step spin-coating route from DMF based precursors composed of phenoxyethylammonium iodide (POEAI), lead iodide (PbI_2_), lead dibromide (PbBr_2_), cesium iodide (CsI) and different amounts of CF3-BSA. The perovskite films prepared with CF3-BSA contents of 2, 4 and 6 mol% relative to the combined molar amount of PbI_2_ and PbBr_2_ are referred to as 2% CF3-BSA, 4% CF3-BSA and 6% CF3-BSA films, respectively. Detailed preparation procedures are described in the methods section. The mixed halides of bromide and iodide are selected to achieve the desired emission color, and the emitters are based on the pure-red quasi-2D perovskites with a composition of (POEA)_2_Cs_*n*-1_Pb_*n*_(Br/I)_3*n*+1_ (*n* represents the number of lead halide octahedra layers). The corresponding chemical structures and simulated electrostatic potential (ESP) distribution of CF3-BSA are presented in Fig. 1a, in which the red and blue regions at EPS indicate the areas of negative and positive charge, respectively. Oxygen and fluorine atoms display negative charge, while H atoms are positively charged. This simulation suggests that CF3-BSA is capable of interact with different species via different sites.

We fabricated PeLEDs based on a structure of glass/indium tin oxide (ITO)/poly (3,4-ethylenedioxythiophene):poly(styrene sulfonate) (PEDOT:PSS)/poly(triaryl amine) (PTAA)/polyvinylpyrrolidone (PVP)/perovskites/2,4,6-tris[3-(diphenylphosphinyl)-phenyl]-1,3,5-triazine (PO-T2T)/LiF/Al (Fig. 1b), and this optimized device structure demonstrates a high level of performance (Supplementary Fig. 1). The perovskite layer consists of discrete nanocrystals, and the formation mechanism will be discussed later. The light out-coupling efficiency of our PeLED was estimated to be ~35%, which falls within a reasonable range due to the discrete structures of the perovskite films (Supplementary Fig. 2). Depth profiles obtained through time-of-flight secondary ion mass spectrometry (ToF-SIMS) verify the chemical composition and elemental distribution across the device stacks (Supplementary Fig. 3). These profiles confirm the incorporation of CF3-BSA molecules throughout the perovskite layer, further supported by corresponding energy-dispersive X-ray spectroscopy (EDS) elemental mappings (Supplementary Fig. 4). The mappings reveal a homogeneous distribution of Br, I, F, and S within the perovskite layer (Supplementary Fig. 5). The I/Br ratio is measured as 2.57:1, closely matching the precursor ratio of I:Br (2.6:1). Supplementary Fig. 6 exhibits the different elemental contents at perovskite nanocrystals and CF3-BSA spacer. The device performance dependents on the molar fraction of CF3-BSA relative to (PbI_2_ + PbBr_2_), with optimal performance achieved at a CF3-BSA molar fraction  of 4% (Supplementary Fig. 7). The perovskite films or devices with this composition are hereafter referred to as CF3-BSA based films or PeLEDs, and the samples without incorporation of CF3-BSA are referred to as control films or PeLEDs. Both control and CF3-BSA based PeLEDs show nearly identical electroluminescence (EL) peaks at 635 nm with Commission Internationale de l’Eclairage (CIE) color coordinates of (0.69, 0.31) near their turn-on voltage, meeting the pure-red Rec. 2020 standards^26,27^ (Supplementary Fig. 8). Moreover, the angle-dependent EL intensity of our PeLEDs is in accord with a lambertian profile (Fig. 1c).

The current density-voltage-luminance (*J*-*V*-*L*) and luminance-EQE (*L*-EQE) characteristics of control and CF3-BSA based PeLEDs are shown in Fig. 1d, e. The CF3-BSA based PeLEDs show peak EQE of 30.2%, and the champion device exhibits a maximum luminance of over 25,000 cd m^-2^. These are substantially higher than that achieved for the control devices (peak EQE 15.9%, maximum luminance of 7866 cd m^−2^). In the pure-red regime (630–650 nm), this combination of high EQE and high luminance in the CF3-BSA based PeLEDs is ahead of previously reported 3D perovskites, quasi-2D perovskites, and colloidal perovskite nanocrystals as far as we know (Fig. 1f, Supplementary Table 1). Furthermore, CF3-BSA based PeLEDs show reasonable reproducibility (Supplementary Fig. 7a, b). On the basis of this strategy, high-performance deep-red PeLEDs emitting at 667 and 654 nm, with EQE of 35.6% and 31.5%, respectively, have also been successfully achieved (Supplementary Fig. 9).

Having established the role of CF3-BSA in modulating quasi-2D perovskite crystallization, we then extended this strategy to large-area pure-red PeLED fabrication. The resulting 3.5 × 3.5 cm^2^ CF3-BSA based film displays bright and spatially uniform red emission under UV illumination (Supplementary Fig. 10a). The film was subsequently sectioned into 25 pieces to assess the emission characteristics across different regions. As shown in Supplementary Fig. 10b, c, these pieces of film exhibit high PLQEs with small variations between different pieces, demonstrating an excellent emission homogeneity of the large-area CF3-BSA based films. It shows that large-area CF3-BSA based films exhibit discrete nanocrystal features (Supplementary Fig. 10d), which is similar to the small-area CF3-BSA based films. We subsequently fabricated large-area pure-red PeLEDs with active areas of 1.0 and 12.25 cm^2^, employing the same device architecture described earlier in this work. These devices achieved peak EQEs of 25.6% and 20.5%, respectively (Fig. 1g, h). In addition, the large-area pure-red PeLED demonstrates an almost uniform luminance distribution across five different positions within the emitting region, highlighting its excellent optical and electrical uniformity (Fig. 1i).

### Stability of the devices

**Fig. 2:**
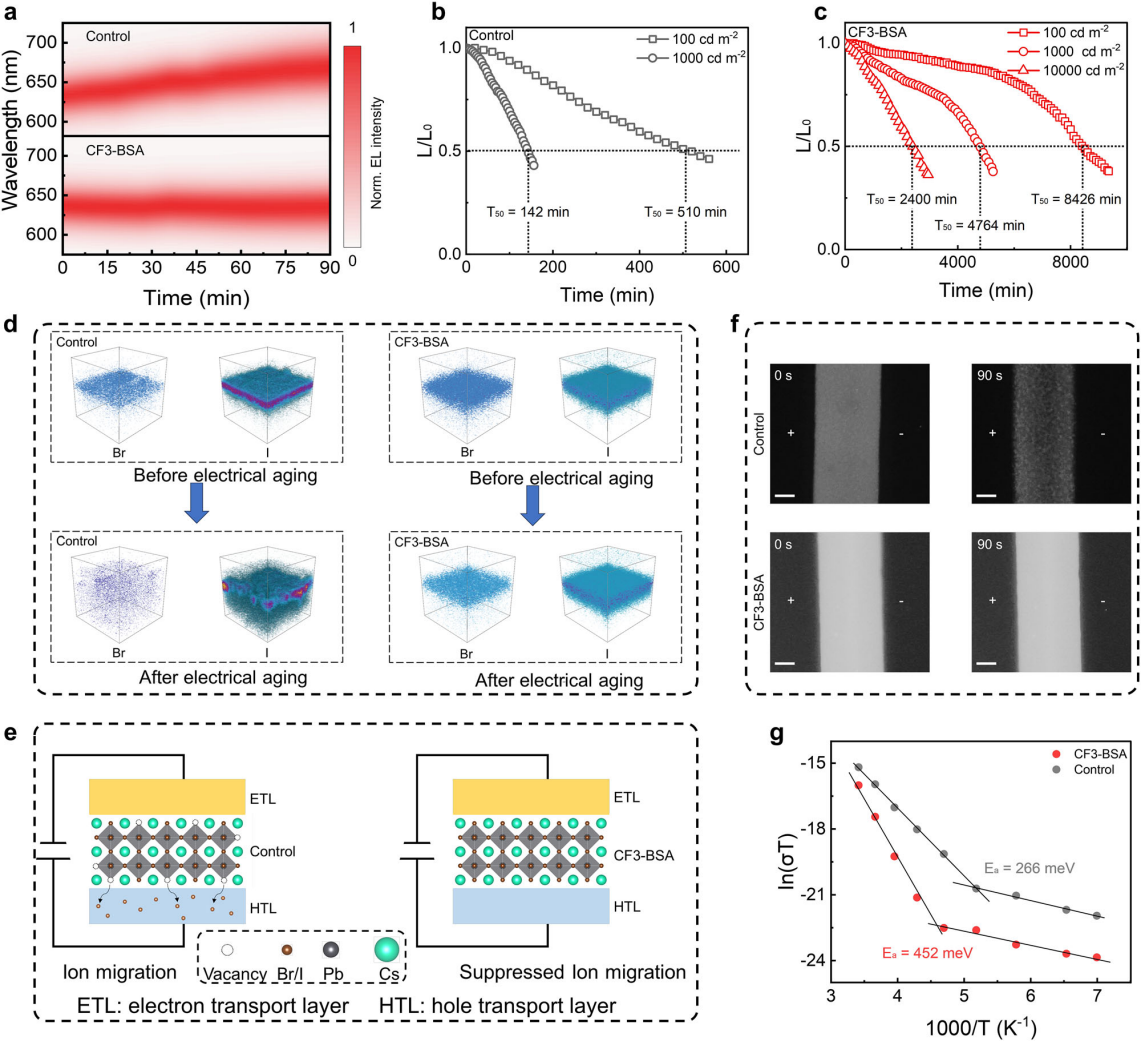
Stability of the PeLEDs. **a** Colormaps of normalized EL spectra with different operating times of the control and CF3-BSA based PeLEDs at constant current of 5 mA cm^−2^. **b** Operational stability measurement of the control PeLED. **c** Operational stability measurement of CF3-BSA based PeLED. **d** Halide ions distribution of the device before and after electrical aging. **e** Scheme illustrating the Br/I migration towards adjacent layers in the control, and the suppressed Br/I ion migration in CF3-BSA based PeLEDs. **f** Microscopic PL imaging of perovskite samples under external electric field (scale bar: 10 μm). **g** Conductivity measurement as a function of temperature for control and CF3-BSA based perovskites.

The operational emission stability for unencapsulated PeLEDs are further investigated inside a nitrogen-filled glove box. We performed the spectral stability measurement of the devices, with both devices initially running at a current density of 5 mA cm^−2^. The EL peak of the control device shifted from 635 to 669 nm over 90 min running, accompanied by a notable broadening of the EL spectrum, with FWHM increasing from 45 to 53 nm (Fig. 2a, Supplementary Fig. 11a), indicating serious halide segregation under electrical bias^28^. By comparison, the EL spectra of the CF3-BSA based PeLEDs remain almost consistent during the same operation time (Fig. 2a, Supplementary Fig. 11b), suggesting that halide ion migration is effectively suppressed in the CF3-BSA based perovskites. Furthermore, the luminance of the control device drops rapidly, with *T*_50_ (the time required for the luminance to drop to 50% of the initial value) of 510 min for the initial value luminance of 100 cd m^−2^ and 142 min for the initial value luminance of 1000 cd m^−2^ (Fig. 2b). In comparison, the CF3-BSA based PeLEDs demonstrate more longer *T*_50_ (8426, 4764, 2400 min for the initial value luminances of 100 cd m^−2^, 1000 cd m^−2^, and 10,000 cd m^−2^) (Fig. 2c), representing one of the most stable pure-red PeLEDs to date (Supplementary Table 1).

The reduced ion migration in CF3-BSA based PeLEDs is further evidenced by TOF-SIMS depth profiling of the PeLEDs and microscopic PL imaging of the perovskite films. The profiling of TOF-SIMS reveals that the control PeLEDs exhibit a broadened Br/I distribution indicative of ion migration towards the adjacent carrier transporting layers after electrical aging, while the CF3-BSA based PeLEDs maintain a consistent Br/I distribution without noticeable changes (Fig. 2d, e). In contrast, the control films showed pronounced PL quenching around the initially formed dark regions, which progressively spread over the perovskite surface during the stability test. This degradation behavior under an external bias serves as direct evidences of ion migration. In contrast, the CF3-BSA based samples showed no significant emission degradation throughout the test (Fig. 2f). We then fabricated parallel devices with a structure of Au/perovskites/Au to determine the activation energy (*E*_a_) of the perovskite films (Fig. 2g). The *E*_a_ was deduced from the Nernst-Einstein equation:

1 $${{\mathrm{ln}}}\,\sigma T=\,{{\mathrm{ln}}}\,{\sigma }_{0}-\frac{{E}_{{{\rm{a}}}}}{{k}_{{{\rm{B}}}}T}$$

where *σ*_0_ is a constant, *k*_B_ is the Boltzmann constant, and *T* is the temperature. *E*_a_ of the CF3-BSA based perovskites was calculated to be 452 meV, which is significantly higher than that of the control films (266 meV). The increased ion-migration activation energy signifies a reconstructed ionic energy landscape induced by the incorporation of CF3-BSA. Specifically, CF3-BSA simultaneously reduces the population of mobile ionic species by eliminating vacancy-mediated migration pathways and increases the energetic cost of halide displacement through stronger local binding interactions, as evidenced in Supplementary Fig. 12. This dual modulation reshapes both defect thermodynamics and migration kinetics, thereby intrinsically suppressing ionic transport and enhancing device stability.

### Optical properties of the perovskite films

**Fig. 3:**
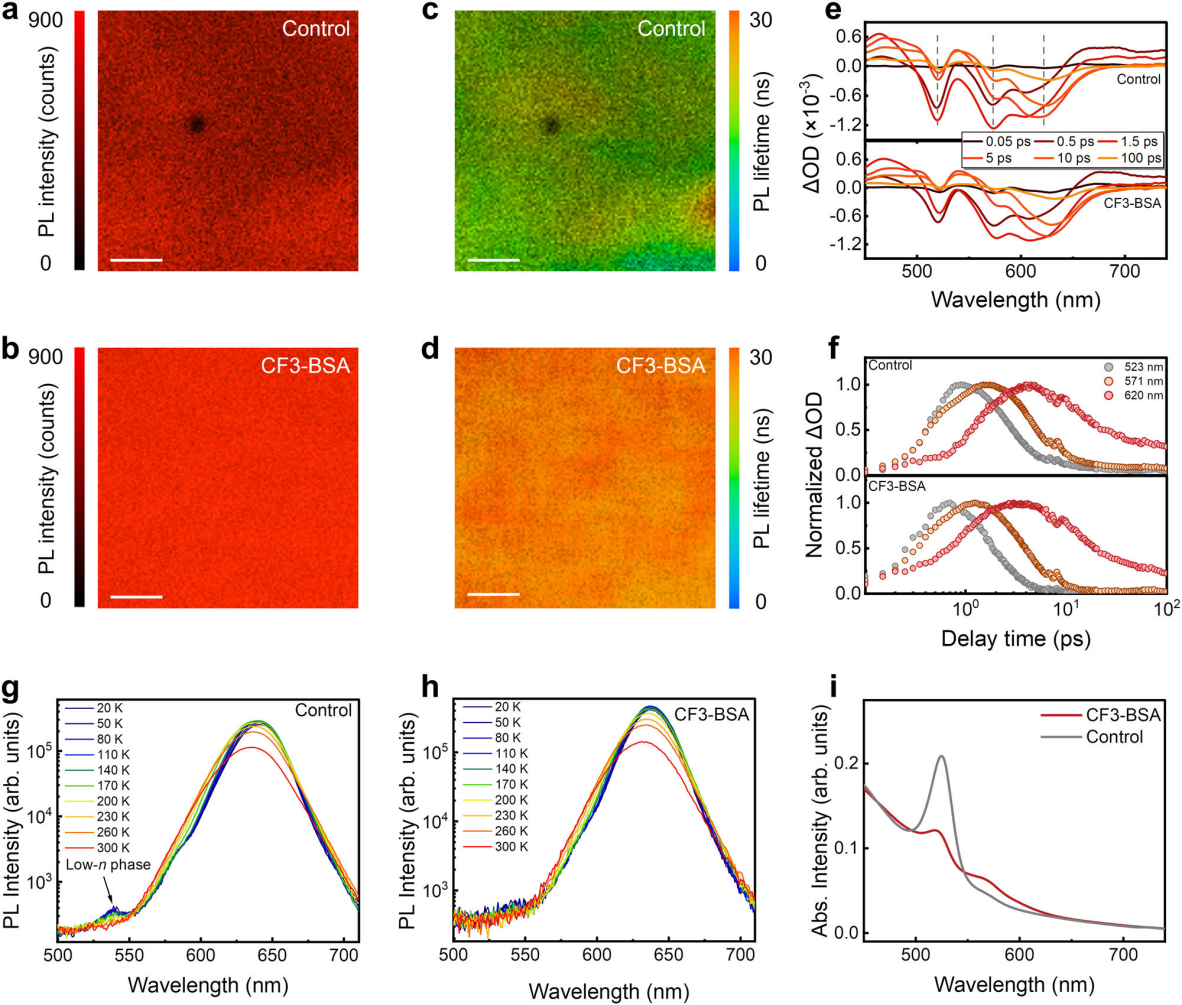
Optical characteristics of the perovskite films. **a**, **b** Confocal PL intensity of the control and CF3-BSA based films (scale bar: 10 μm). **c**, **d** Confocal PL lifetimes of the control and CF3-BSA based films (scale bar: 10 μm). **e** TA spectra at different probe delay times and **f** TA kinetics probed at selected wavelengths for the control and CF3-BSA based films. Temperature-dependent PL spectra of (**g**) control and **h** CF3-BSA based films excited at 365 nm. **i** UV-vis absorption spectra of the control and CF3-BSA based films.

Confocal PL microscopy reveals pronounced micrometer scale emission heterogeneity in the control films, with dark domains corresponding to defect rich regions that promote non-radiative recombination (Fig. 3a). By contrast, the CF3-BSA based films show a substantially more uniform PL distribution over the same spatial range, with no obvious dark features observed (Fig. 3b). This improved emission uniformity agrees well with the prolonged carrier lifetime extracted from time resolved PL measurements (Fig. 3c, d and Supplementary Fig. 13). Moreover, the CF3-BSA based films retain high PLQEs over a broad excitation power range, reaching 95.7% at an excitation intensity of 21.8 mW cm^−2^ (Supplementary Fig. 14). These results indicate that defect mediated non-radiative losses are effectively suppressed by CF3-BSA (Supplementary Figs. 15–20 and Supplementary Note 1).

To further probe the excited state evolution, we carried out ultrafast transient absorption (TA) spectroscopy on both films. The ground state photobleaching (PB) features located at ~523, ~571 and ~620 nm can be assigned to the low-*n* phase and mixed higher-*n* species in the quasi-2D perovskites (Fig. 3e). Immediately after photoexcitation, the *n* = 2 PB signal develops within 0.05 ps, indicating that carriers are initially generated predominantly in low-*n* domains. With increasing delay time, the PB signals from low-*n* phases gradually decay, accompanied by the growth of the high-*n* component (*n* ≥ 4). This spectral evolution evidences rapid exciton transfer from low-*n* to high-*n* phases. Notably, the CF3-BSA based films exhibit a weaker *n* = 2 bleaching signal than the control films, suggesting that CF3-BSA reduces the population of low-*n* phases. Such suppression is beneficial for carrier transport in quasi-2D perovskite films.

The charge carrier transfer processes in both control and CF3-BSA based films were further studied by extracting the decay time of different-*n* phases (Fig. 3f). The fitted kinetics further quantify the exciton-transfer process. For the control film, the fast decay components (*τ*_1_) for 523 and 571 nm, corresponding to the *n* = 2 and mixed high-*n* phases, are 0.17 and 0.29 ps, respectively, while the PB rise time (*τ*_et_) for the *n* ≥ 4 phase is 0.71 ps. After CF3-BSA treatment, both the decay of low-*n* signals and the formation of high-*n* bleaching become faster, with *τ*_1_ reduced to 0.11 ps for 523 nm and 0.22 ps for 571 nm, and *τ*_et_ shortened to 0.63 ps. These kinetic changes indicate that CF3-BSA optimizes the phase distribution and facilitates energy transfer toward high-*n* emissive domains in quasi-2D perovskites (Supplementary Fig. 21).

Temperature-dependent PL spectroscopy was then used to probe emissive species that are not readily resolved at room temperature, because interphase charge and energy transfer in mixed-dimensional perovskites is strongly affected by temperature^29^. With decreasing temperature, the control film develops an additional high-energy emission band centered at ~540 nm, which can be attributed to low-*n* phases (Fig. 3g). In contrast, no extra emission feature is detected in the CF3-BSA based film over the same temperature range (Fig. 3h), further confirming that the formation of low-*n* domains is effectively suppressed by CF3-BSA. UV-vis absorption spectra were subsequently collected to examine the excitonic absorption features associated with different phase components in the films (Fig. 3i). For both the control and CF3-BSA based films, the absorption spectra exhibit a shoulder at around 520 nm, corresponding to the *n* = 2 layered perovskite phase. Additionally, the shoulder at 566 nm and a red tail extending to ~625 nm are associated with mixed high-*n* phases of the quasi-2D perovskites. Compared with the control films, the absorption peak for the *n* = 2 phase in CF3-BSA based films is clearly weakened, while the absorption peak for the high-*n* phases is enhanced. It has been reported that suppressing low-*n* phases is conducive to energy transfer in quasi-2D perovskites because the low-*n* phases usually exhibit low conductivity^30–32^.

### Growth of the perovskite films

**Fig. 4:**
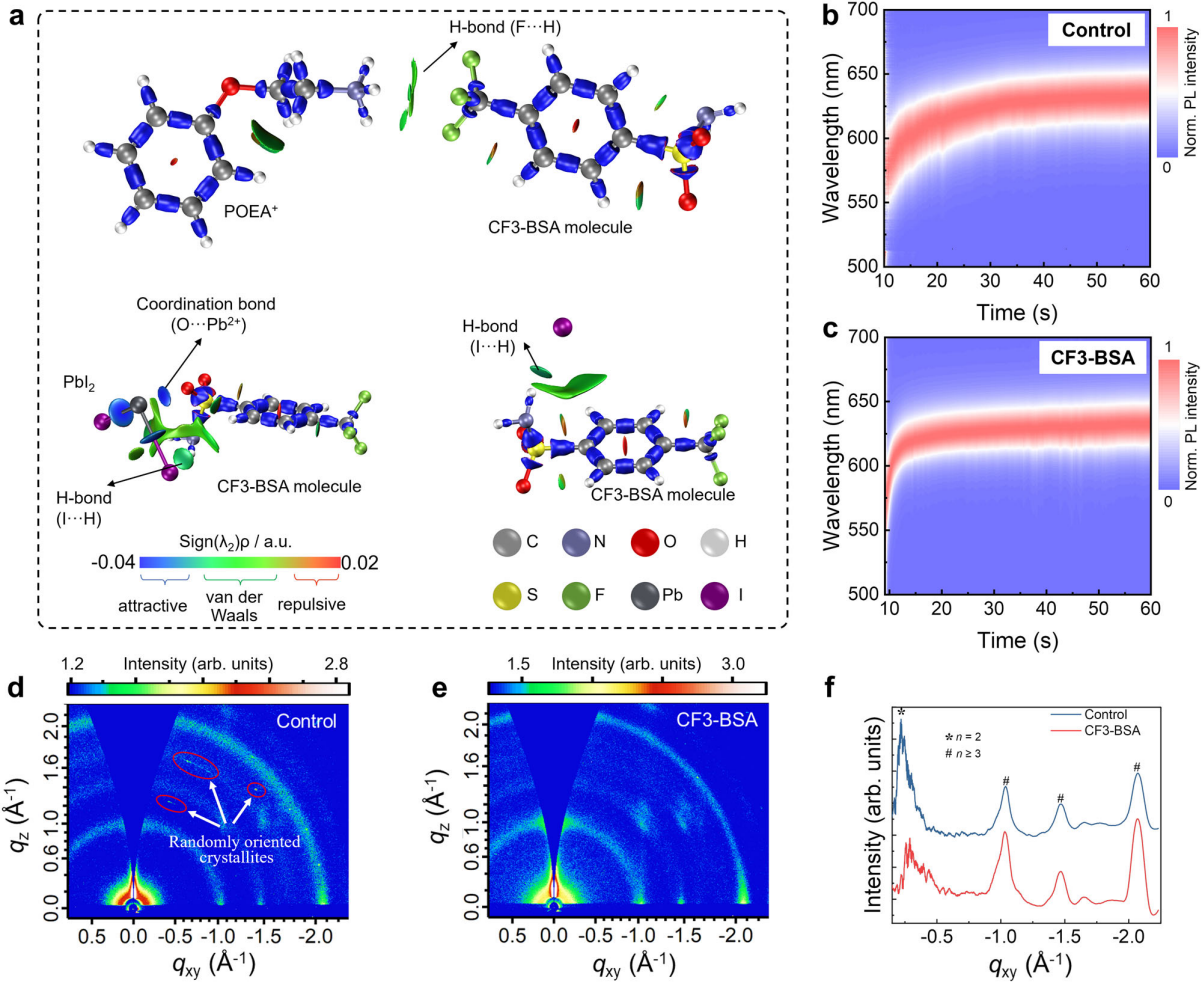
The influence of CF3-BSA on the growth of quasi-2D perovskite films. **a** Visualization of weak interactions between CF3-BSA and perovskite precursors by the IRI analysis. Normalized in-situ PL measurements of the (**b**) control and (**c**) CF3-BSA based films under UV light irradiation. 2D GIWAX of the (**d**) control and (**e**) CF3-BSA based films. **f** GIWAXS data integrated for the perovskite films.

To examine how the CF3-BSA molecule affects the phase distributions, we initially utilized a solvation model density based on the Interaction Region Indicator (IRI) to visualize the weak interactions (Fig. 4a) and the interaction energies (Supplementary Fig. 22) between the CF3-BSA molecule and the perovskite precursors. IRI analysis was performed using Multiwfn software^33^, and the resulting isosurfaces were visualized as color mapped surfaces in Visual Molecular Dynamics (VMD). We find that the CF3-BSA molecule can interact with perovskite precursors via multiple bonding forms. For example, the amide hydrogen of the CF3-BSA molecule and iodide ions can form a hydrogen bond (H-bond) of N-H···I^-^ with an interaction energy of 5.013 kcal mol^−1^; the interaction energy between POEA^+^ and CF3-BSA is 3.649 kcal mol^−1^, which derives from H-bond (C-F···H^+^) between POEA^+^ and the C-F bond in CF3-BSA; and there is a chelation interaction (with interaction energy of 8.148 kcal mol^−1^) between sulfonyl oxygen and lead cation (S = O:Pb^2+^). The interactions between CF3-BSA and perovskite precursors are also verified by the experimental results of solution nuclear magnetic resonance (Supplementary Fig. 23, Supplementary Note 2).

We then used in situ PL spectroscopy to track the crystallization process and elucidate the role of CF3-BSA during perovskite film formation (Fig. 4b, c and Supplementary Fig. 24). At the early stage of growth, both films display an emission maximum near 550 nm, characteristic of low-*n* phase of quasi-2D perovskites, followed by a comparable shift in PL peak position over time. However, the CF3-BSA modified perovskite films reach a stable PL peak at an earlier stage of crystallization than the control sample (Supplementary Fig. 25a). Moreover, the PL intensity of the CF3-BSA based films increases more rapidly during crystallization (Supplementary Fig. 25b). The accelerated PL redshift, together with the faster intensity buildup, indicates that CF3-BSA promotes the early formation of high-*n* phases at the initial crystallization stage. This behavior reflects a more homogeneous phase evolution and demonstrates effective regulation of the quasi-2D perovskite crystallization kinetics by CF3-BSA.

We subsequently conducted morphological characterizations of the perovskite films using scanning electron microscope (SEM). The addition of CF3-BSA induced a transition from continuous polycrystals with distinct grain boundaries to discrete nanocrystals (Supplementary Fig. 26). Due to the capability of CF3-BSA molecules to regulate the perovskite nanocrystal growth, increasing the molar ratios of CF3-BSA to (PbI_2_ + PbBr_2_) from 2 to 6% leads to a gradual average size decrease of nanograins from 21.6 to 10.1 nm (Supplementary Fig. 27). Interestingly, their PL peak positions remain unaffected by particle size (Supplementary Fig. 28). This phenomenon can be explained by photogenerated carriers transitioning from a low-*n* phase and ultimately recombining in a high-*n* phase due to the energy funneling effect. The above observation is obviously different from the 3D perovskite nanocrystals exhibiting quantum confinement effects^34–36^. Moreover, CF3-BSA based film exhibit a higher exciton binding energy (*E*_b_) (Supplementary Fig. 29), indicating that the nanocrystal structure experiences a stronger quantum confinement effect due to the grain size reduction in CF3-BSA based perovskite films.

We examined the structural characteristics of the films through X-ray diffraction (XRD), which revealed a prominent peak corresponding to the (200) plane in CF3-BSA based films. This observation indicates significantly enhanced crystallinity compared to the control films (Supplementary Fig. 30). However, the intensity of the (200) peak diminished with increasing CF3-BSA concentration to 6%, signifying that an excessive amount of CF3-BSA molecule could compromise the crystalline quality of perovskites. 2D grazing-incidence wide-angle X-ray scattering (GIWAXS) measurements reveal oriented growth in both perovskite films (Fig. 4d, e). Compared with the CF3-BSA films, the control films exhibit additional diffraction rings with extended segments (highlighted by red circles in Fig. 4d), indicating a higher fraction of randomly oriented crystallites^37^. Furthermore, the intensity of the diffraction peak at 0.227 Å^−1^ notably decreased in the CF3-BSA based films (Fig. 4f), suggesting effective suppression of the formation of low-*n* phase^38–40^. This enhanced structural order and reduced phase heterogeneity facilitate efficient charge transport and energy funneling, thereby contributing to the improved efficiency and operational stability of the PeLEDs.

Based on the above analysis, the CF3-BSA molecule contains three functional groups. Firstly, the electropositive amide hydrogen could form hydrogen bonds with halide ions, thus preventing halide segregation of the mixed-halide perovskite under electric field^41,42^. Secondly, electronegative fluorine atoms could form hydrogen bonds with the organic cations of POEA^+^, which would control the diffusion of organic cations during films deposition, realizing the goal of quasi-2D perovskite with energetically monodisperse quantum wells^40^. Thirdly, sulfone (S = O) could bind to uncoordinated lead ions at the perovskite grain boundaries, suppressing the non-radiative recombination channels and enhancing the PLQE of perovskites^43^. Moreover, the interaction between sulfone and lead ions helps avoid the reduction of Pb^2+^ to Pb^0^, contributing to the stability of perovskite films^44^, thereby promoting high crystallinity and uniform nanocrystal morphology (Supplementary Fig. 31).

### Design principle for additives

**Fig. 5:**
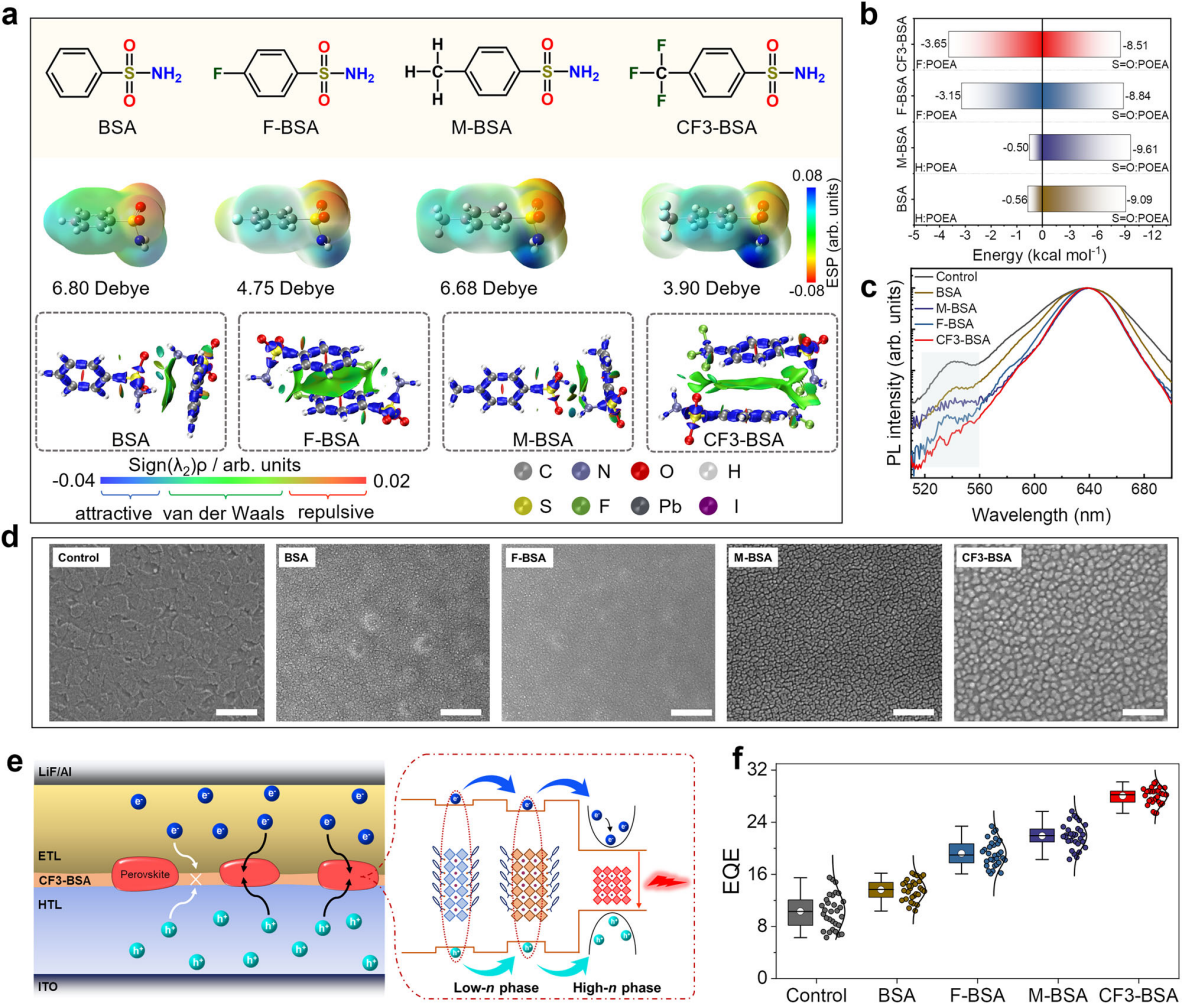
Interactions between analogous additives and perovskites. **a** Chemical structures, electrostatic potential (ESP) distributions, and IRI analyses of BSA, F-BSA, M-BSA, and CF3-BSA. **b** Calculated interaction energies between the additives and the spacer cation POEA^+^. **c** PL spectra of the perovskite films incorporated with different additives. **d** SEM images of the corresponding films (scale bar: 100 nm). **e** Schematic of the device architecture and cascade energy transfer among different *n*-phases in quasi-2D perovskites. **f** Statistics of EQEs of the PeLEDs based on different additives. For the box plots, the central line denotes the median, and the square indicates the mean. The lower and upper bounds of the box represent the 25th and 75th percentiles, respectively. The solid lines extending above and below the box indicate the maximum and minimum values, respectively.

To elucidate how molecular structure governs the crystallization of quasi-2D perovskite films, we introduced four analogous sulfonamide based additives with systematically varied dipole moments and steric profiles (Fig. 5a). Additives with reduced dipole moments have been shown to regulate spacer-cation diffusion during quasi-2D perovskite deposition, a process that is critical for achieving high-performance PeLEDs^45,46^. BSA and M-BSA exhibit relatively large dipole moments (6.80 and 6.68 D), whereas fluorinated derivatives, particularly CF3-BSA, display significantly reduced dipole moments (3.90 D) together with pronounced steric hindrance arising from the bulky CF3 substituent.

We then quantified the interactions between the additives and the organic spacer cation (POEA^+^). CF3-BSA exhibits the weakest interaction energy between the sulfonamide group and POEA^+^ (Fig. 5b), indicating that the sulfonamide moiety remains more accessible for defect passivation through coordination with Pb^2+^ ions. In contrast, stronger sulfonamide-cation interactions in BSA and M-BSA diminish their passivation efficiency. Meanwhile, CF3-BSA shows the strongest hydrogen-bonding interactions through its fluorine groups with organic cations, which effectively regulate spacer-cation diffusion and suppress the formation of low-*n* phases (Fig. 5c), as evidenced by the reduced *n* = 2 domain emission (~540 nm).

Furthermore, CF3-BSA and F-BSA form ordered molecular assemblies via hydrogen bonding (F···H) and π-π stacking interactions, as revealed by IRI analysis (Fig. 5a), whereas BSA and M-BSA predominantly exhibit disordered NH···π interactions. This molecular ordering translates into improved crystallographic orientation, consistent with GIWAXS results showing reduced random crystallite orientation in CF3-BSA and F-BSA based films (Supplementary Fig. 32). Owing to its larger steric hindrance, CF3-BSA further drives a transition from continuous polycrystalline films to nanocrystal architectures, as confirmed by SEM imaging (Fig. 5d).

Such nanocrystal architectures suppress interfacial quenching, promote balanced carrier injection, and enhance exciton radiative recombination (Fig. 5e). Meanwhile, CF3-BSA functions as a physical spacer between charge-transport layers, eliminating interfacial nonradiative pathways and enabling higher PeLED performance (Fig. 5f). To validate the generality of this design principle, nine additional analogous molecules were evaluated (Supplementary Figs. 33–35), confirming that the multifunctional CF3-BSA molecule delivers the superior optoelectronic performance (Supplementary Note 3). Moreover, sky-blue emitting (~478 nm) and green emitting (~518 nm) PeLEDs incorporating CF3-BSA also exhibit substantial performance enhancements (Supplementary Fig. 36). Collectively, these results demonstrate that the combination of large steric hindrance and ordered molecular assembly in additives plays a decisive role in regulating crystallization pathways and carrier recombination in PeLEDs, which is essential for achieving high-performance devices.

In summary, we introduce a multifunctional molecule of CF3-BSA to regulate the growth of pure-red quasi-2D perovskite films. CF3-BSA  combines large steric hindrance and ordered molecular assembly in precursor solutions, and simultaneously interactions with POEA^+^ spacer cations, Pb^2+^ ions, and halide ions. These features effectively modulate crystallization dynamics of the perovskites, enabling the controlled growth of quasi-2D perovskites with a  nanocrystalline morphology and enhanced light outcoupling. As a result, CF3-BSA enhances the crystallinity and PLQE of the films by passivating uncoordinated Pb^2+^ sites, thereby improving device stability and performance. Moreover, the interaction between CF3-BSA and perovskite precursors promotes phase redistribution in quasi-2D perovskite films, suppressing low-*n* phases and enhancing energy transfer efficiency. With this approach, we successfully achieved stable (8426 min) pure-red PeLEDs with both  high luminance (25,133 cd m^-2^) and EQE (30.2%). Moreover, we demonstrated large-area (12.25 cm^2^) pure-red PeLEDs with EQE exceeds 20%. This study provides a universal guideline for tuning the growth dynamics of quasi-2D perovskite films to achieve high-performance PeLEDs.

## Methods

### Materials

2-Phenoxyethylamine (POEA), 4-(trifluoromethyl)benzenesulfonamide (CF3-BSA), PbI_2_, PbBr_2_, CsI, and N,N-dimethylformamide (DMF, anhydrous, 99.9%) were purchased from Sigma-Aldrich. Poly(triaryl amine) (PTAA), poly(3,4-ethylenedioxythiophene):poly(styrenesulfonate) (PEDOT:PSS, Al 4083), and 2,4,6-tris[3-(diphenylphosphinyl)phenyl]-1,3,5-triazine (PO-T2T) were purchased from Xi’an Polymer Light Technology. All materials were directly used without further purification.

### Synthesis of organic ammonium salt of POEAI

For the preparation of POEAI, POEA (4 mmol) was first dissolved in 5 mL of ethanol, and an excess amount of aqueous hydroiodic acid (45 wt%) was then introduced rapidly under cooling. After stirring the reaction mixture at 0 °C for 2 h, 60 mL of diethyl ether was added to precipitate the product. The obtained solid was washed three times with diethyl ether and subsequently vacuum dried at room temperature.

### Precursor solution preparation

For the preparation of pure-red quasi-2D perovskite precursor solutions, the I/Br molar ratio was maintained at 2.6:1. POEAI, CsI, PbI_2_, PbBr_2_ were dissolved in DMF at a molar ratio of 1:0.6:0.5:0.5, with a precursor concentration of 0.1 mmol mL^−1^. For CF3-BSA based perovskites, CF3-BSA was introduced into the precursor solutions at different molar percentages relative to the total lead content, namely 2%, 4% and 6%. All solutions were stirred for 2 h and filtered through a 0.45 μm polytetrafluoroethylene filter before use. For perovskites with emission spectra of deep-red (EL peak at 653 nm), the molar ratio of iodide and bromide was fixed to be 5:1. POEAI, CsI, PbI_2_, PbBr_2_ were dissolved in DMF with a molar ratio of 1:0.6:0.7:0.3 with a concentration of 0.1 mmol mL^−1^. For perovskites with deep-red emission spectra (EL peak at 662 nm), the emitters are pure-iodide quasi-2D perovskites. POEAI, CsI, PbI_2_ were dissolved in DMF with a molar ratio of 1:0.6:1 with a concentration of 0.1 mmol mL^−1^. The sky-blue quasi-2D perovskite precursor solution was achieved with 0.067 mmol PEABr, 0.05 mmol EABr, 0.133 mmol CsBr, 0.1 mmol PbBr_2_ in 1 mL dimethyl sulfoxide under continuous stirring overnight at room temperature. The green quasi-2D perovskite precursor solution was achieved with 0.08 PEABr, 0.2 mmol CsBr, 0.2 mmol PbBr_2_ in 1 mL dimethyl sulfoxide under continuous stirring overnight at room temperature. Mole fractions of CF3-BSA relative to the lead (4%) were added into the perovskite precursor solutions.

### Device fabrication

Patterned indium tin oxide (ITO) coated glass substrates were cleaned sequentially by ultrasonication in aqueous detergent, acetone, deionized water and ethanol for 30 min each. After drying, the substrates were subjected to UV-ozone treatment for 15 min before device fabrication. The PeLEDs were constructed with the architecture of ITO/PEDOT:PSS/PTAA/PVP/perovskite/PO-T2T/LiF/Al. PEDOT:PSS was spin-coated onto the ITO substrates at 4000 r.p.m. for 60 s, followed by drying at 140 °C for 15 min in air. The PTAA was dissolved into toluene at 1 mg mL^−1^, and the PTAA layer was deposited onto the PEDOT:PSS layer at 4000 r.p.m. for 60 s and annealing at 100 °C for 10 min. PVP dissolved in anhydrous ethanol at 2 mg mL^−1^ was spin-coated onto the PTAA layer at 4000 r.p.m. for 60 s and then annealed at 100 °C for 5 min. The perovskite films were subsequently deposited onto the PEDOT:PSS/PTAA coated substrates by spin coating at 4000 r.p.m. for 120 s and then treated at 80 °C for 10 min. Finally, PO-T2T (40 nm), LiF (1 nm), and Al (100 nm) were deposited by thermal evaporation at a vacuum pressure below 4 × 10^−4 ^Pa. The device had an active area of 0.07 cm^2^ by the overlapping area of Al and ITO electrodes. The structure of large-area device was the same as that described above. For hole-only devices with the structure of ITO/PTAA/perovsktie/TCTA/MoO_3_/Au, PTAA and perovskite were spin coated onto ITO as described above, then the layers of TCTA (40 nm), MoO_3_ (5 nm) and Au (100 nm) were thermally evaporated at a vacuum pressure below 4 × 10^−4 ^Pa.

### Characterization of LED performance

All PeLED measurements were carried out on unencapsulated devices in a nitrogen-filled glove box at room temperature. The Current density-voltage-luminance (*J-V-L*) curves, EQEs, EL spectra, and CIE coordinates of the PeLEDs were recorded simultaneously with a calibrated commercial testing platform comprising a Keithley 2400 source meter, a QE-Pro spectrometer (Ocean Optics) and an integrating sphere (SPECTRUMTEQ-EL-Int-3.3). The reproducibility of the device performance was verified by independent measurements conducted at Zhengzhou University and the University of Cambridge (Supplementary Table 2). Device lifetimes were evaluated under nitrogen at room temperature using a commercial LED ageing system supplied by Guangzhou Xi Pu Optoelectronics Technology Co., Ltd.

### Material characterizations

Absorption and photoluminescence (PL) spectra were recorded on a Shimadzu UV-3600 ultraviolet-visible-near-infrared (UV-vis-NIR) spectrophotometer and an Edinburgh FLS1000 fluorescence spectrometer, respectively. The in-situ PL characterization of the perovskite films during the spin-coating process were conducted on a self-built system, which consists of a light source with 365 nm LED, a QE-Pro spectrometer (Ocean Optics), some optical fibers, and a computer, as shown in Supplementary Fig. 24. The PLQE values were determined with an integrating sphere, using a 405 nm continuous-wave laser as the excitation source. Confocal PL maps were acquired by PicoQuant MicroTime 200. The samples were excited with a 405 nm pulsed laser operating at 5 MHz and 1.8 pJ pulse^−1^, focused through a ×100 objective with an NA of 0.9. The PL signals were collected through the same objective, a 600 nm long pass filter and a 75 μm pinhole. Time-resolved PL decay was measured using time-correlated single-photo counting by Edinburgh FLS1000 with a 401 nm laser. Time-resolved PL decay curves were fitted to a biexponential decay function. TEM and HRTEM images of the perovskite samples were acquired using a Tecnai G2 20 U-Twin transmission electron microscope operated at 200 kV. SEM images were collected on an FEI Quanta 450 FEG microscope, and the particle sizes were analysed using ImageJ software. XRD patterns were recorded on a Bruker SMART-CCD diffractometer with Cu Kα radiation (*λ* = 0.15418 nm). XPS and UPS measurements were conducted using an AXIS-ULTRA DLD-600W photoelectron spectrometer (Kratos, Japan). FTIR spectra were measured at room temperature on a Bruker Vertex 70 spectrometer equipped with a PIKE MIRacle ATR accessory with a diamond prism and a DLaTGS detector. NMR spectra were obtained using a Bruker 600 MHz spectrometer (Avance Neo 600), with the mixtures dissolved in DMSO-d_6_. The XEUSS SAXS/WAXS system was used to perform the GIWAXS measurement. The incidence angle of X-ray beam with the wavelength of 1.54 Å was set to be 0.2° for the perovskite thin films prepared on Si substrates. ToF-SIMS was conducted using a PHI 7200 instrument (Physical Electronics). The analysis region was set to 50 × 50 μm^2^ within a sputtered area of 350 × 350 μm^2^. The optical constants of the perovskite films, including the refractive index *n* and extinction coefficient *k*, were determined using a dual rotating-compensator Mueller matrix ellipsometer (ME-L ellipsometer, Wuhan Eoptics Technology, China).

The *n* and *k* values of other functional layers are obtained from simulation software database. PL spectrum used for light outcoupling efficiency simulation of the PeLEDs was measured by the Setfos-Pholos integration of the Phelos system. In the end, the light outcoupling efficiency was simulated by Setfos simulation software.

### TA measurements

The fs-TA experiments were carried out on a Helios transient absorption spectrometer (Ultrafast Systems LLC) driven by a Coherent amplified femtosecond laser system. Pump excitation at 380 nm was supplied by a TOPAS-800-fs optical parametric amplifier, delivering approximately 1 nJ pulse^−1^ at the sample. The amplifier was pumped by a Ti:sapphire regenerative amplifier (Legend Elite-1K-HE; 800 nm, 35 fs, 7 mJ pulse^−1^, 1 kHz), which was seeded with a mode-locked Ti:sapphire oscillator (Micra 5) and pumped by an Nd:YLF laser (Evolution 30). A broadband white-light continuum probe spanning 420–780 nm was generated by directing a small fraction of the 800 nm amplifier output, approximately 400 nJ pulse^−1^, onto a sapphire plate. A reference channel split from the continuum probe was used to compensate for pulse-to-pulse intensity fluctuations. Pump-probe delay times were adjusted with a motorized optical delay stage, and the instrument response function was estimated to be approximately 100 fs from cross-correlation measurements.

### UPS measurements of the bottom interface of the perovskite film

The perovskite film was first spin-coated onto an ITO substrate, and the UPS spectrum of the top surface was collected directly from the as-prepared film. To probe the bottom interface, the film was subjected to controlled Ar^+^ ion etching for 90 s before the second UPS measurement.

### DFT calculations

The theoretical calculations of visualization of weak interactions between CF3-BSA and perovskite precursors were performed via the density functional theory (DFT) programs^47^. The structures of the studied molecule and its dimer were fully optimized at the B3LYP-D3BJ/def2-SVP level of theory. The solvent effect (DMF) was included in the calculations using the solvation model based on the density (SMD) model. The vibrational frequencies of the optimized structures were carried out at the same level. All optimized geometries were confirmed to correspond to local minima on the potential energy surface by the absence of imaginary vibrational frequencies. To visualize the interaction regions within the chemical systems, IRI analysis was performed, which enables weak interactions and chemical bonds to be identified in real space^48^. The IRI data were calculated using Multiwfn software^33^, and the corresponding isosurface maps were rendered as color-filled surfaces with Visual Molecular Dynamics (VMD)^49^. In the IRI plots, blue, green and red regions represent hydrogen-bonding interactions, van der Waals interactions and steric effects, respectively. The optimized atomic coordinates of the molecular interaction models are provided in Supplementary Data 1.

We performed the first-principles calculations in the DFT for the interaction between perovskite and CF3-BSA^50^. The exchange-correlation interactions were treated using the Perdew-Burke-Ernzerhof (PBE) functional within the generalized-gradient approximation (GGA)^50,51^. For CsPbI_3_, structural relaxation was performed by optimizing all atomic coordinates while keeping the lattice parameters fixed. The relaxation was considered converged when the total-energy change between successive ionic steps was below 10^−4 ^eV and the residual force on each atom was less than 0.015 eV Å^−1^. A plane-wave kinetic-energy cutoff of 400 eV was used. The *k*-point mesh was chosen to ensure a spacing smaller than 0.03 Å^−1^ throughout the Brillouin zone^52^. For the isolated CF3-BSA molecule, geometry optimization was carried out in a 15 Å×15 Å×15 Å cubic cell.

To calculate the adsorption energy of the CF3-BSA molecule on the iodine defective CsPbI_3_ surface, the corresponding slab models were first constructed. To obtain the most stable adsorption configurations, structural optimizations were performed with the middle three layers fixed and a vacuum thickness of 15 Å. The adsorption energies were calculated by the following equation:

2 $$\Delta E={E}_{{{\rm{adsorbate}}}}+{E}_{{{\rm{slab}}}}-{E}_{{{\rm{system}}}}$$

where, *E*_system_, *E*_slab_ and *E*_adsorbate_ represent the total energies of the adsorption system, substrate surface and adsorbate, respectively. The ion migration barriers were investigated using the climbing image nudged elastic band (CI-NEB) method^53^ with dipole correction. The convergence criteria for the CI-NEB calculations were set to 10^−5^ eV for the total energy and 0.05 eV Å^−1^ for the residual forces.

## Supplementary information

Supplementary Information

Description of Additional Supplementary Files

Supplementary Data 1

Transparent Peer Review file

## Source data

Source Data

## Data Availability

The data that support the findings of this study are available from the corresponding author on request. Source data are provided with this paper.
