# Supplementary material for: High-performance red light-emitting diodes from quasi-two-dimensional perovskite nanocrystals
Source: Nat Commun. 2026 May 7;17:6175. doi: 10.1038/s41467-026-72733-w (PMC13365440; doi:10.1038/s41467-026-72733-w)
Supplement: Supplementary file 1 — Supplementary Information [file 41467_2026_72733_MOESM1_ESM.docx]

**High-performance red light-emitting diodes from quasi-two-dimensional perovskite nanocrystals**

Jibin Zhang^1,2^, Tianjun Liu^3^, Qichun Gu^4^, Shuailing Lin^1^, Jian Mao^4,5^, Zimu Wei^4^, Meng Wang^1^, Yang Lu^4^, Bo Cai^6^, Zher Ying Ooi^4^, Alessandro Mirabelli^4^, Linyuan Lian^1^, Miguel Anaya^4,7^, Ying Liu^1^, Mochen Jia^1^, Xu Chen^1^, Yanbing Han^1^, Xinzhen Ji^1^, Yiwei Zhang^8^, Zhuangzhuang Ma^1^, Xinping Zhang^8^, Xin Zhou^9^, Xinjian Li^1^, Fanglong Yuan^10*^, Lintao Hou^11*^, Chongxin Shan^1*^, Neil C. Greenham^3^, Samuel D. Stranks^3,4^, Zhifeng Shi^1^^*^

^1^Key Laboratory of Materials Physics of Ministry of Education, School of Physics, Zhengzhou University, Zhengzhou 450001, China.

^2^Faculty of Informatics and Engineering, The University of Electro-Communications, 1-5-1 Chofugaoka, Chofu, Tokyo 182-8585, Japan.

^3^Cavendish Laboratory, University of Cambridge, Cambridge, UK.

^4^Department of Chemical Engineering and Biotechnology, University of Cambridge, Cambridge, UK.

^5^State Key Laboratory of Photovoltaic Science and Technology, Shanghai Frontiers Science Research Base of Intelligent Optoelectronics and Perception, Institute of Optoelectronics, Fudan University, Shanghai 200438, China

^6^Department of Materials and Metallurgy, University of Cambridge, Cambridge, UK.

^7^Departamento Física de la Materia Condensada, Instituto de Ciencia de Materiales de Sevilla, Universidad de Sevilla-CSIC, Calle Américo Vespucio 49, Sevilla 41012, Spain

^8^Institute of Information Photonics Technology, Beijing University of Technology, Beijing, China.

^9^Guangdong Provincial Key Laboratory of Semiconductor Micro Display, Foshan Nationstar Optoelectronics Company Ltd., Foshan 528000, China

^10^Key Laboratory of Theoretical & Computational Photochemistry of Ministry of Education, College of Chemistry, Beijing Normal University, Beijing, China.

^11^Guangzhou Key Laboratory of Vacuum Coating Technologies and New Energy Materials, College of Physics and Optical Engineering, Jinan University, Guangzhou, China.

**Correspondence to**

F.Y. flyuan@bnu.edu.cn

L.H. thlt@jnu.edu.cn

C.S. cxshan@zzu.edu.cn

Z.S. shizf@zzu.edu.cn

**Contents**

Supplementary Fig. 1…………………………………………………………….3

Supplementary Fig. 2…………………………………………………………….4

Supplementary Fig. 3…………………………………………………………….5

Supplementary Fig. 4…………………………………………………………….6

Supplementary Fig. 5…………………………………………………………….7

Supplementary Fig. 6…………………………………………………………….8

Supplementary Fig. 7…………………………………………………………….9

Supplementary Fig. 8………………………………………………………….10

Supplementary Fig. 9………………………………………………………….11

Supplementary Fig. 10…………………………………………..………………12

Supplementary Fig. 11……………………………………..……………………13

Supplementary Fig. 12……………………………………..……………………14

Supplementary Fig. 13……………………………………..……………………15

Supplementary Fig. 14……………………………………..……………………16

Supplementary Fig. 15……………………………………..……………………17

Supplementary Fig. 16……………………………………..……………………18

Supplementary Fig. 17……………………………………..……………………19

Supplementary Fig. 18……………………………………..……………………20

Supplementary Fig. 19……………………………………..……………………21

Supplementary Fig. 20…………………………………………..………………22

Supplementary Fig. 21…………………………………………..………………23

Supplementary Fig. 22…………………………………………..………………24

Supplementary Fig. 23…………………………………………..………………25

Supplementary Fig. 24…………………………………………..………………26

Supplementary Fig. 25…………………………………………..………………27

Supplementary Fig. 26…………………………………………..………………28

Supplementary Fig. 27…………………………………………..………………29

Supplementary Fig. 28…………………………………………..………………30

Supplementary Fig. 29…………………………………………..………………31

Supplementary Fig. 30…………………………………………..………………32

Supplementary Fig. 31…………………………………………..………………33

Supplementary Fig. 32…………………………………………..………………34

Supplementary Fig. 33…………………………………………..………………35

Supplementary Fig. 34…………………………………………..………………36

Supplementary Fig. 35…………………………………………..………………37

Supplementary Fig. 36…………………………………………..………………38

Supplementary Table 1.……………………………….………..………………39

Supplementary Table 2………………….…………….………..………………40

Supplementary Note 1…………………………………………..……………41

Supplementary Note 2…………………………………………..……………44

Supplementary Note 3…………………………………………..……………45

Supplementary References……………………………………..……………………48


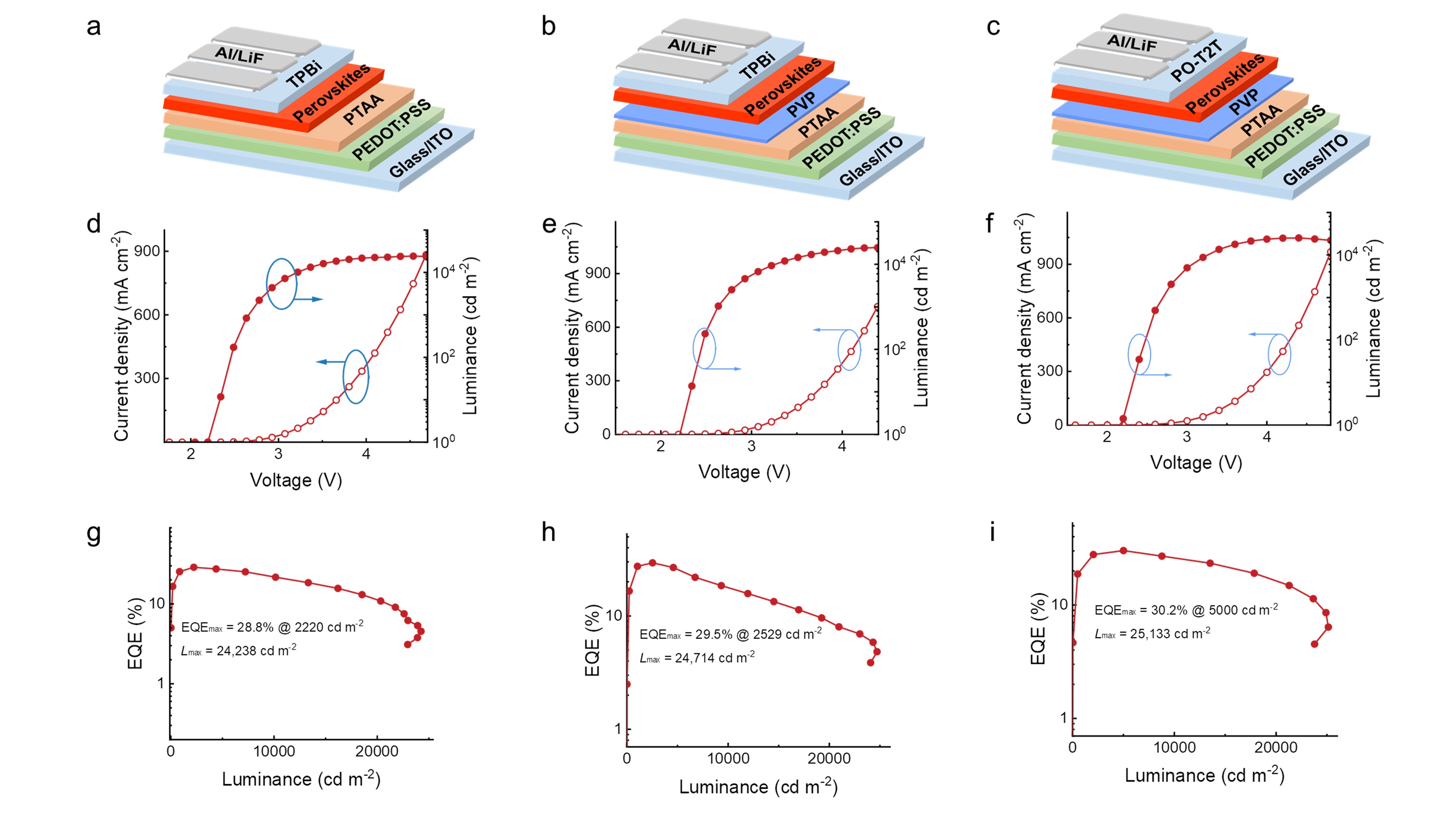


**Supplementary Fig. 1| Performances of the PeLEDs with different structures.**

**a** ITO/PEDOT:PSS/PTAA/Perovskites/TPBi/LiF/Al.

**b** ITO/PEDOT:PSS/PTAA/PVP/Perovskites/TPBi/LiF/Al.

**c** ITO/PEDOT:PSS/PTAA/Perovskites/POT2T/LiF/Al.


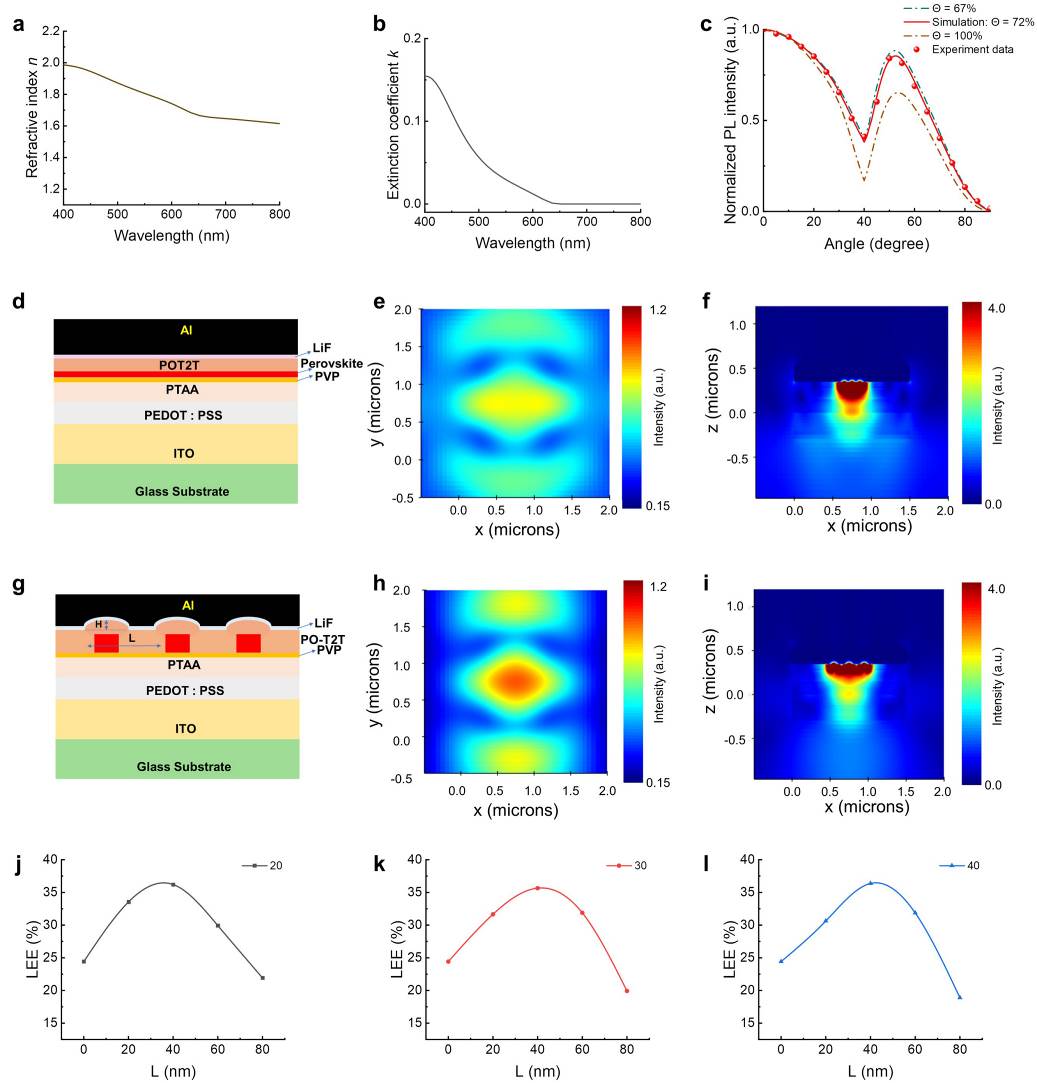


**Supplementary Fig. 2****| Light out-coupling efficiency of the PeLED. a** Refractive index *n* and (**b**) extinction coefficient *k* of the perovskite emissive layer. **c** Angle-dependent PL intensity measurements of perovskite film. Angle-dependent PL data points fitted by classical electromagnetic dipole model show the ratio of horizontal TDMs to vertical TDMs in the perovskite film is 72%, slightly higher than that of conventional isotropic emitters (~67%). **d** Device structure of the continuous perovskite film. **e,f** Stimulated optical distributions from **d**. **g** Device structure of the discontinuous perovskite film. **h,i** Stimulated optical distributions from **g. j-l** Calculated outcoupling efficiency as a function of period *L* with convex height *h* = 20, 30, 40 nm. The reference is a device made from continuous perovskite film. The simulation shows that the outcoupling efficiency can be more than 30% over a wide range of periods from 20 to 60 nm.


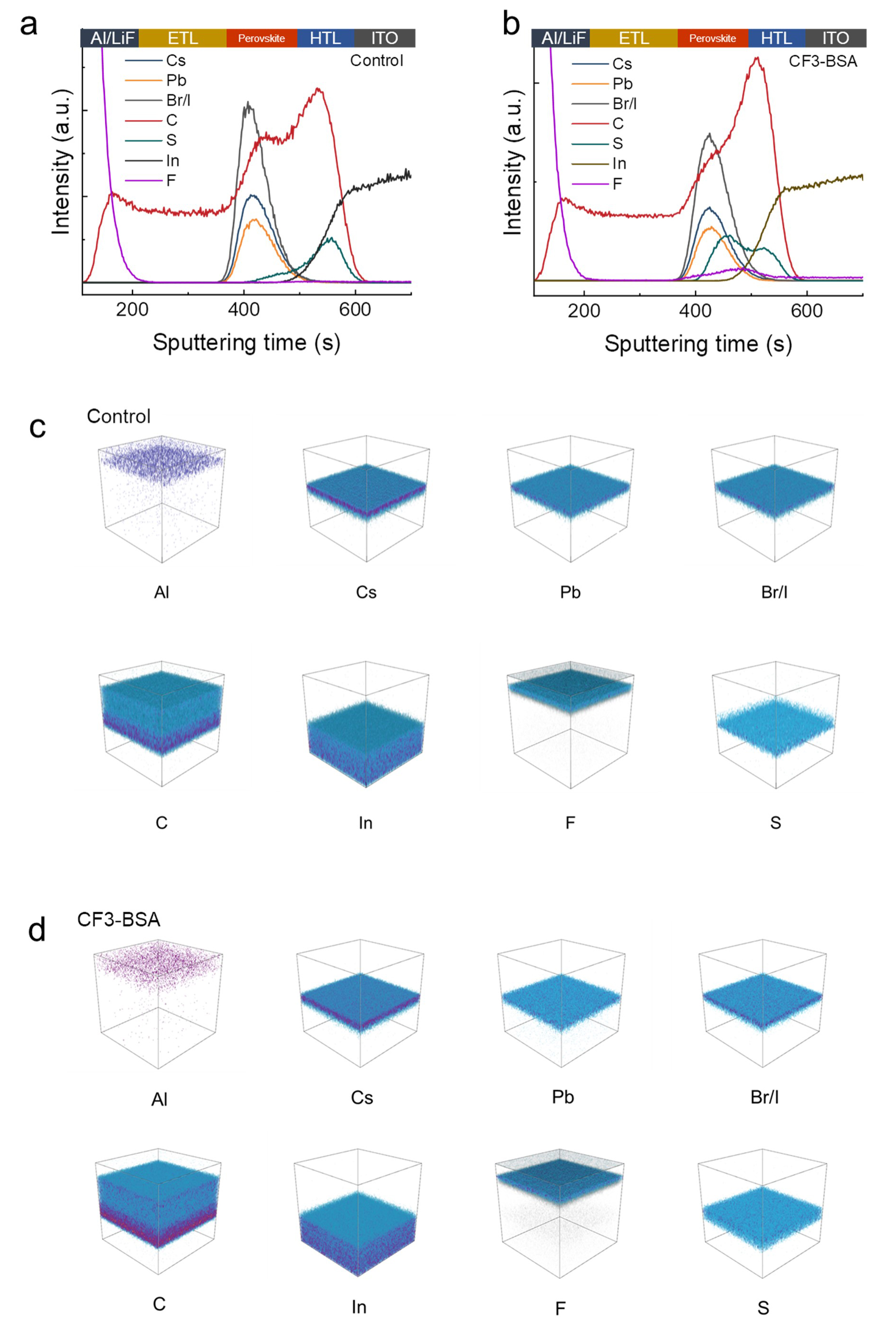


**Supplementary Fig. 3| ToF-SIMS analysis of the devices.** Corresponding 2D and 3D ToF-SIMS depth profiles showing the distribution of elements throughout the (**a,c**) control and (**b,d**) CF3-BSA based PeLEDs.


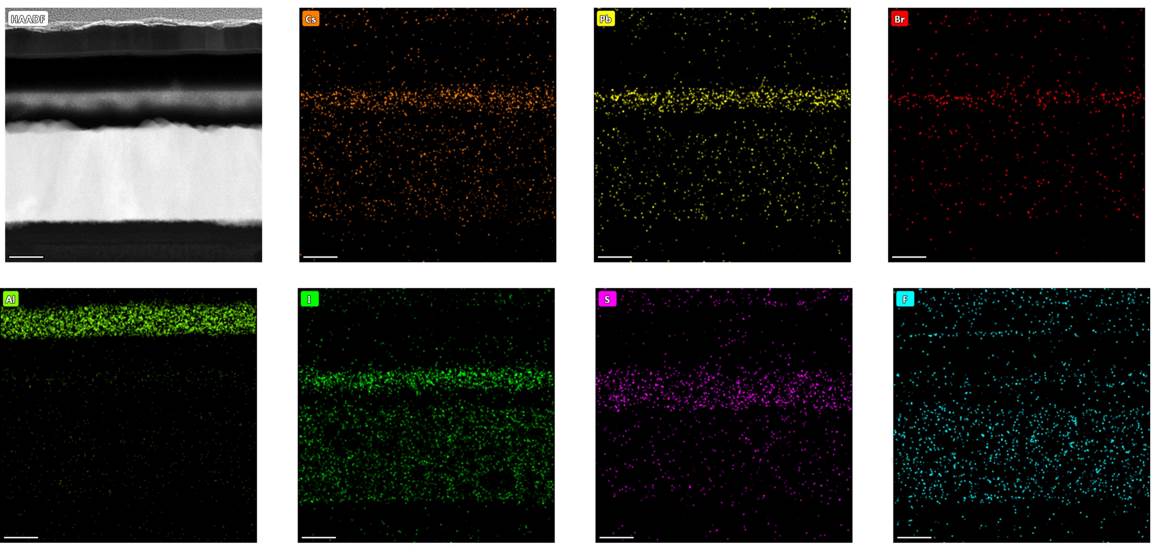


**Supplementary Fig. 4| Elemental distribution in the device.** HADDF-STEM image and the EDS elemental mappings of Cs, Pb, Br, Al, I, S, F in the CF3-BSA based PeLEDs (scale bar: 50 nm)


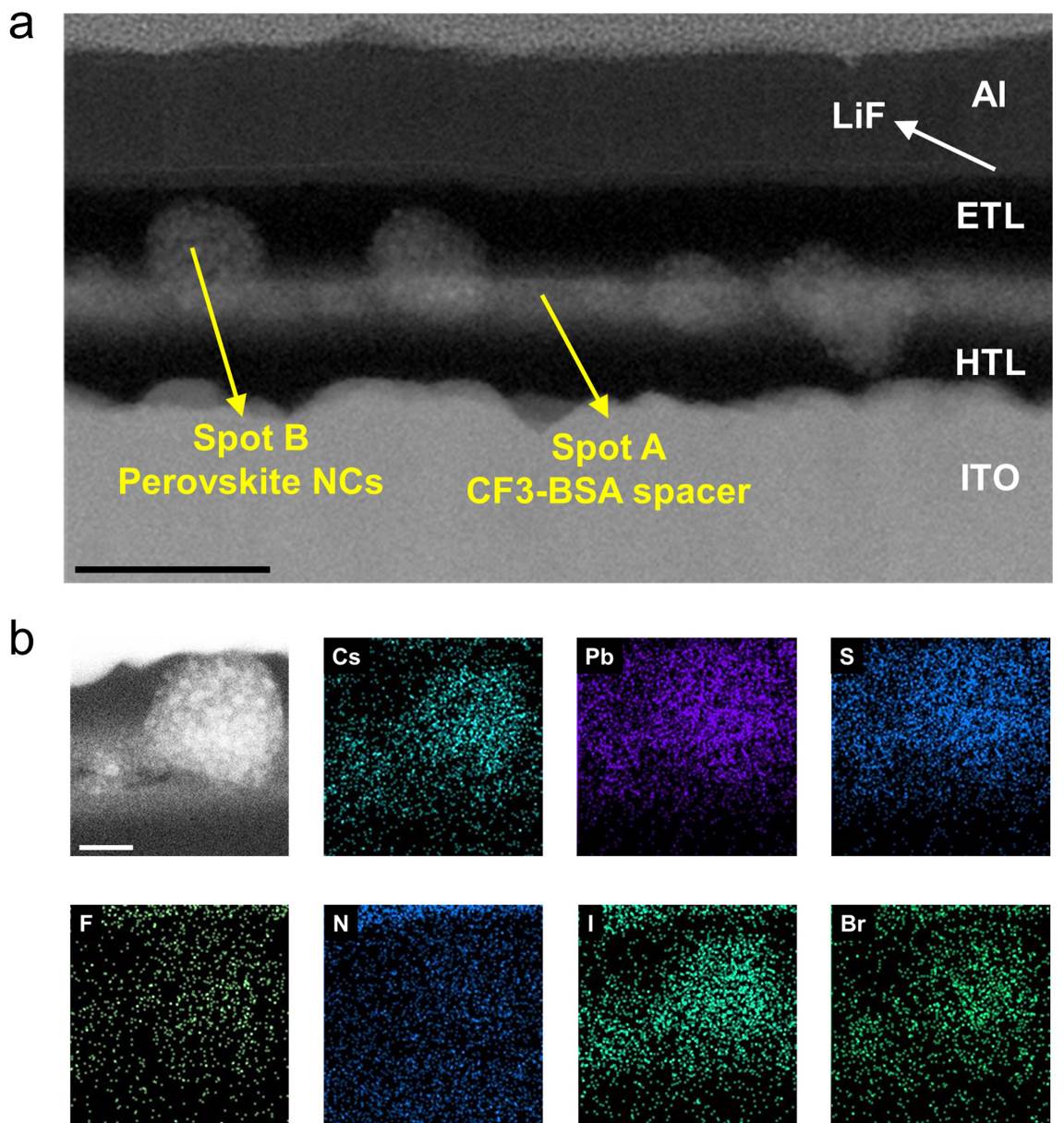


**Supplementary Fig. 5| Elemental distribution in** **one perovskite particle. a** Cross-sectional HRTEM image of the CF3-BSA based PeLEDs (scale bar: 50 nm). **b** Energy-dispersive spectroscopy (EDS) elemental mappings of Cs, Pb, S, F, N, I, Br. (scale bar: 10 nm)


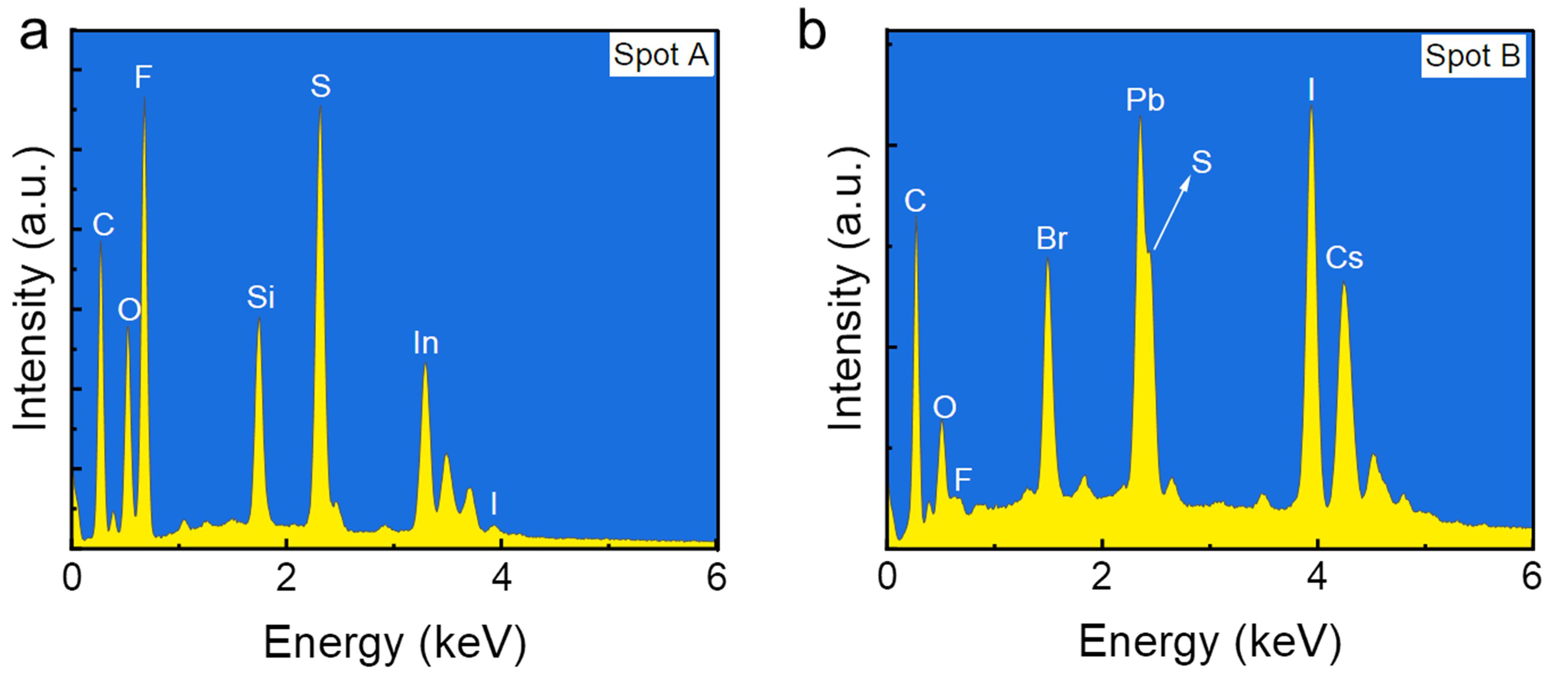


**Supplementary Fig. 6| Comparison elemental distribution.** EDS data captured at two spots as marked in Supplementary Fig. 5a.


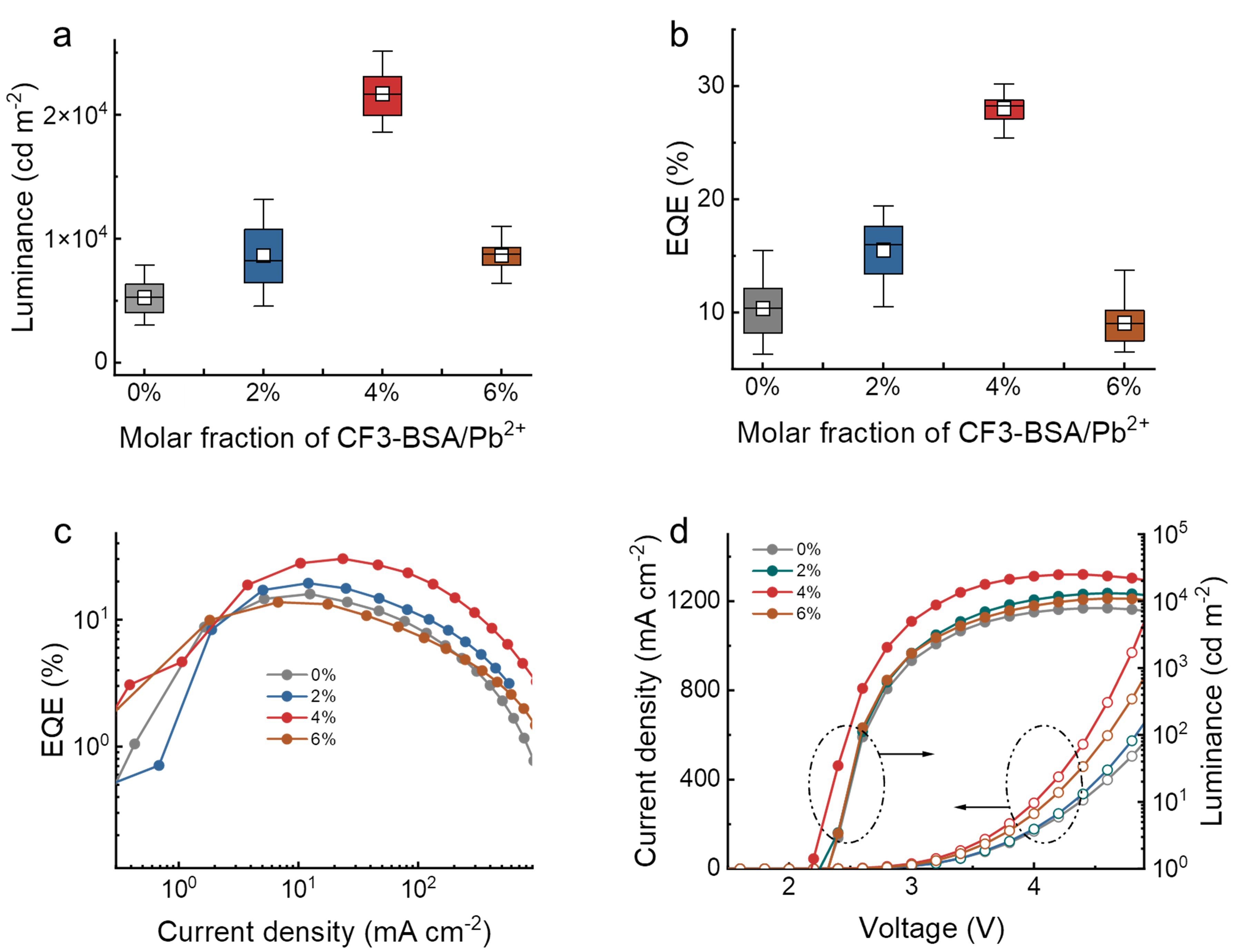


**Supplementary Fig. 7| Performance of PeLEDs with different CF3-BSA.** Statistics of peak (**a**) luminance and (**b**) EQEs measured from 30 individual PeLEDs fabricated with various amounts of CF3-BSA additive. For the box plots, the central line denotes the median, and the square indicates the mean. The lower and upper bounds of the box represent the 25th and 75th percentiles, respectively. The solid lines extending above and below the box indicate the maximum and minimum values, respectively. **c** EQE *versus* current density characteristics of the champion PeLEDs fabricated with various amounts of CF3-BSA additive. **d** Current density *versus* voltage and luminance *versus* voltage characteristics of the champion PeLEDs fabricated with various amounts of CF3-BSA additive.


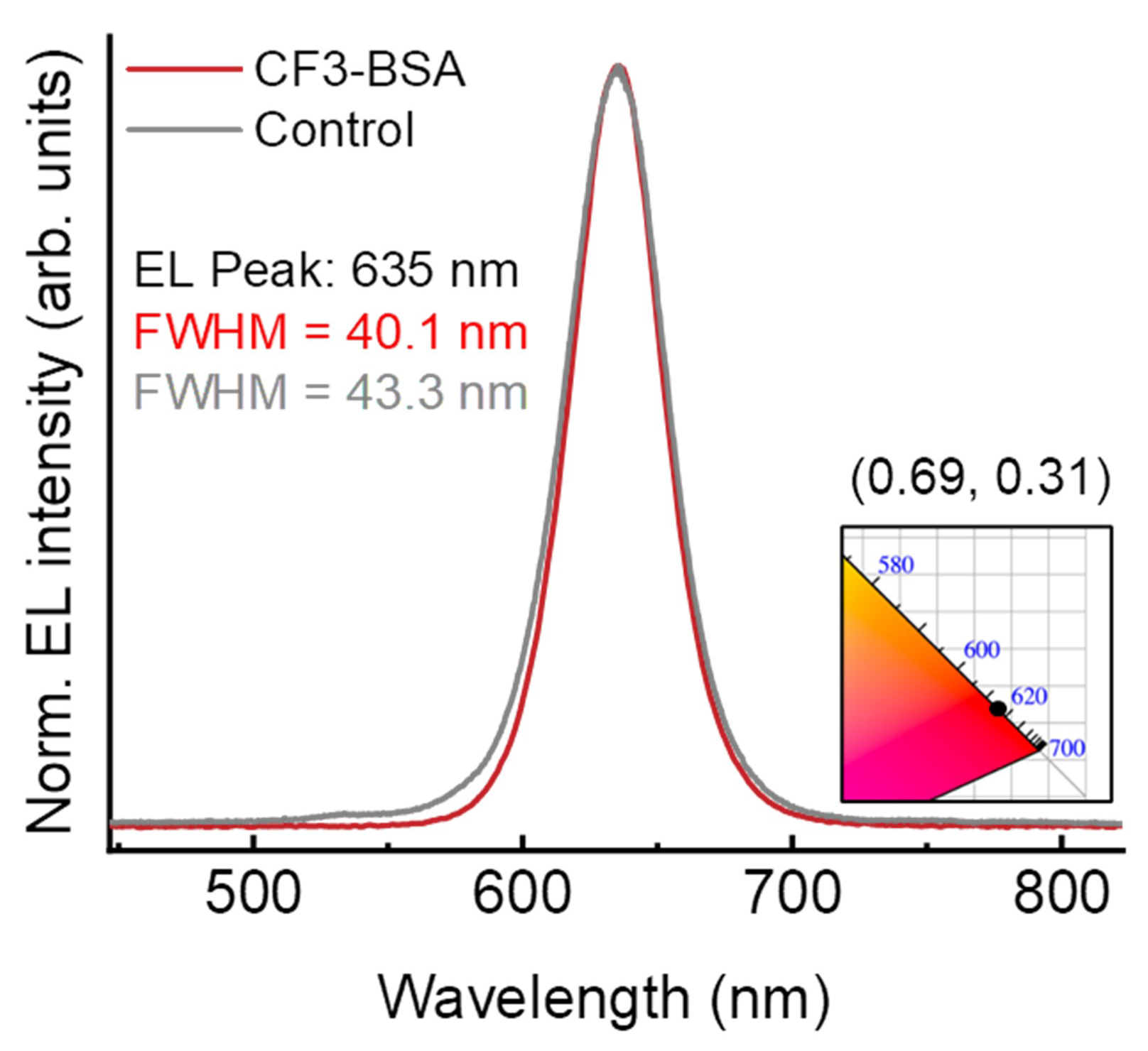


**Supplementary Fig. 8| Spectra character of the device.** EL spectra of control and CF3-BSA based PeLEDs at operating voltage of 2.5 V. Inset show the Commission Internationale de l’Eclairage (CIE) color coordinates of the device.


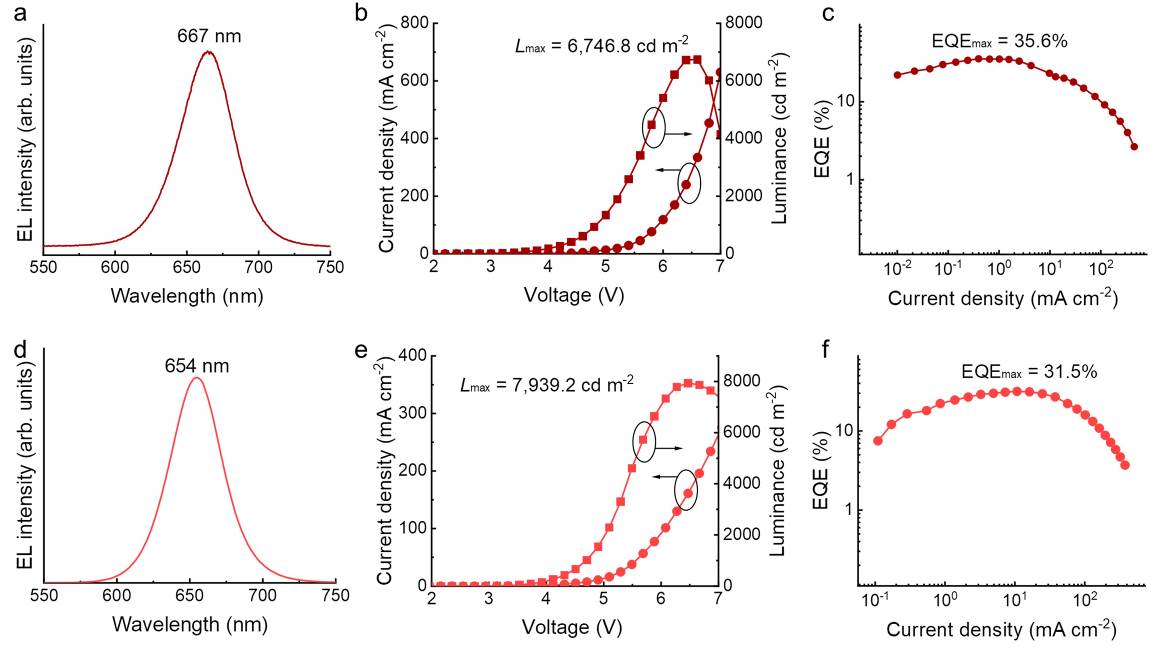


**Supplementary Fig. 9| Performance of the deep-red PeLEDs.** Characteristics of CF3-BSA based deep-red PeLEDs emitting at (**a-c**) 667 nm, (**d-f**) 654 nm. **a,d** EL intensity of the PeLEDs. **b,e** Current density *versus* voltage and luminance *versus* voltage characteristics of the PeLEDs. **c,f** EQE *versus* current density characteristics of the PeLEDs.


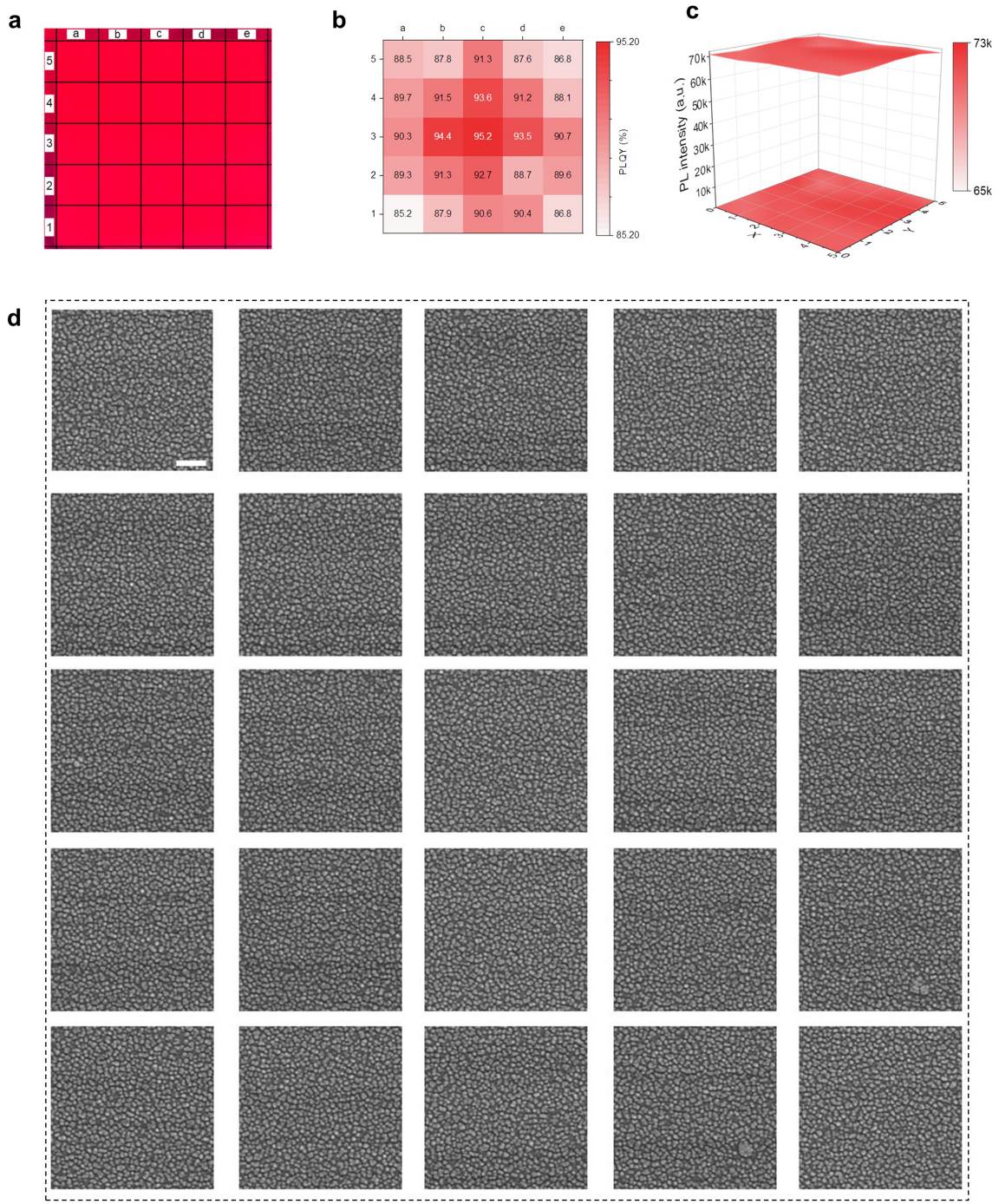


**Supplementary Fig. 10| Uniformity analysis of large-area perovskite film. a** Digital photo of the resulting large-area CF3-BSA film (3.5 cm × 3.5 cm) under 365 nm UV irradiation. Statistical (**b**) PLQE and (**c**) PL intensity in selected regions for the 25 pieces films. **d** SEM image of CF3-BSA based film in selected regions for the 25 pieces films (scale bar: 100 nm).


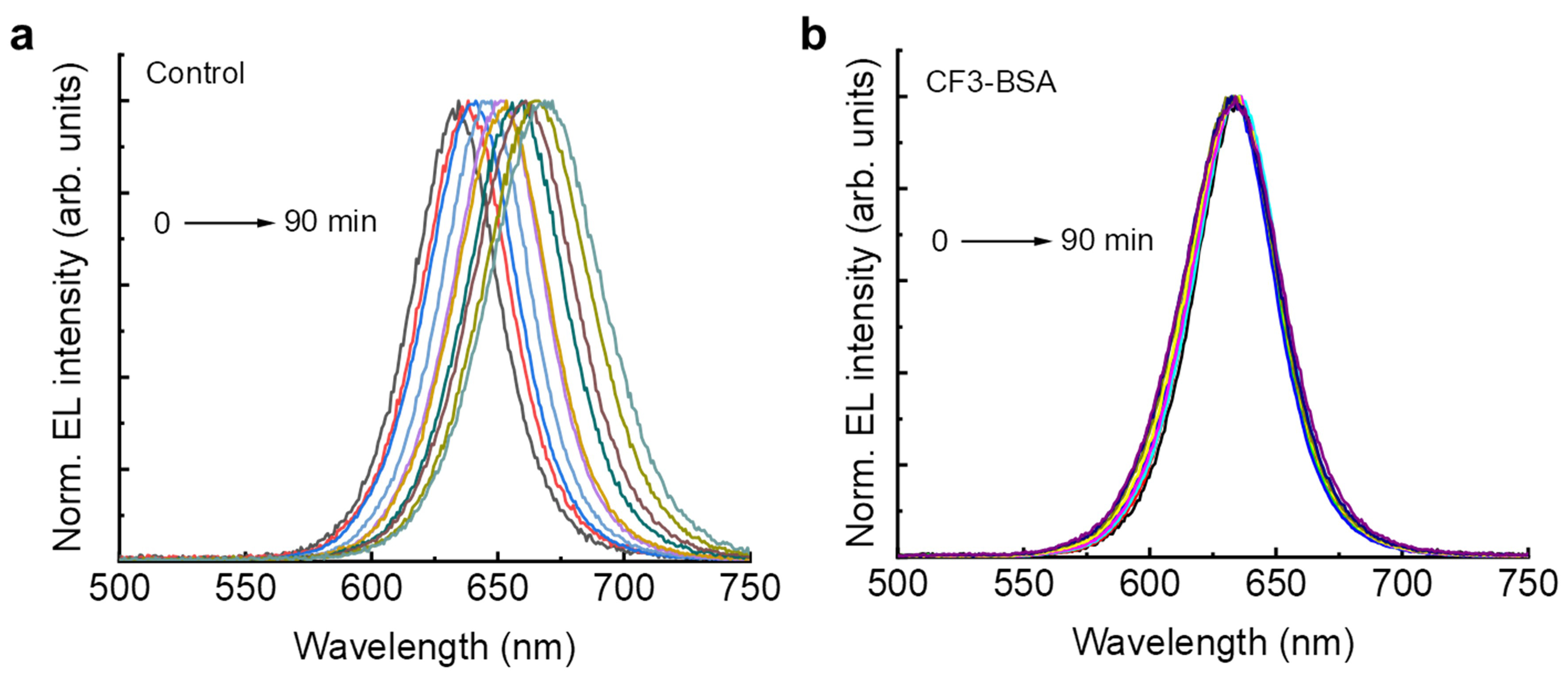


**Supplementary Fig. 11| EL spectra of the devices.** Normalized EL intensity of (**a**) control and (**b**) CF3-BSA based PeLEDs at different operating time.


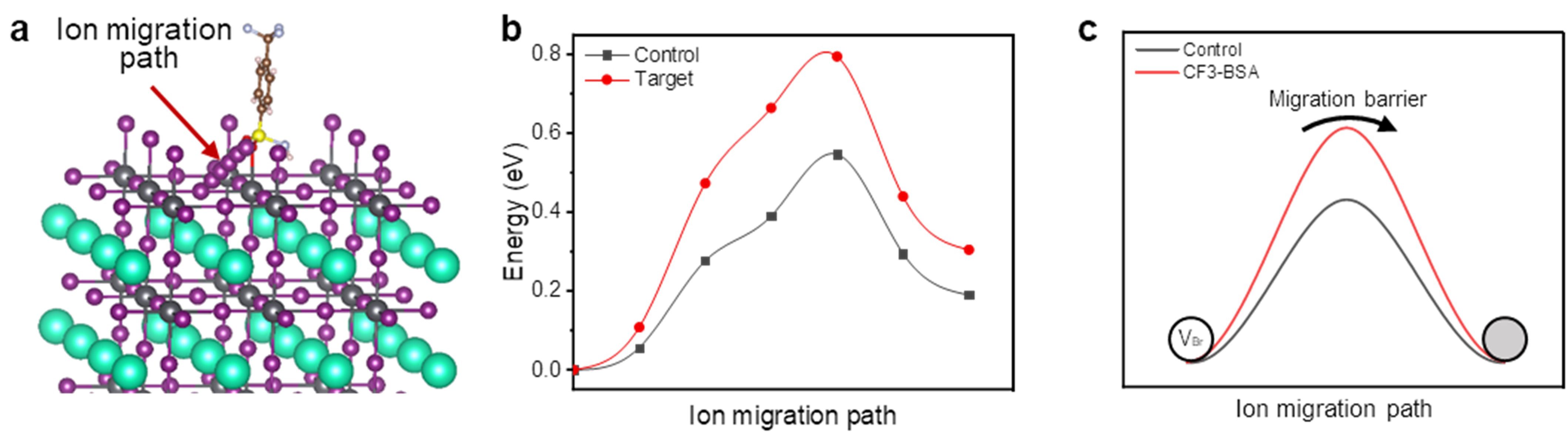


**Supplementary Fig. 12| Ion migration path of the halides. a** Illustration of halide ion vacancy migration pathway on the surface of the perovskites. **b** Calculated energy profile along the corresponding ionic migration pathway and (**c**) schematic of the migration barrier of halide ion vacancies on the surface of perovskites before and after treated by CF3-BSA.


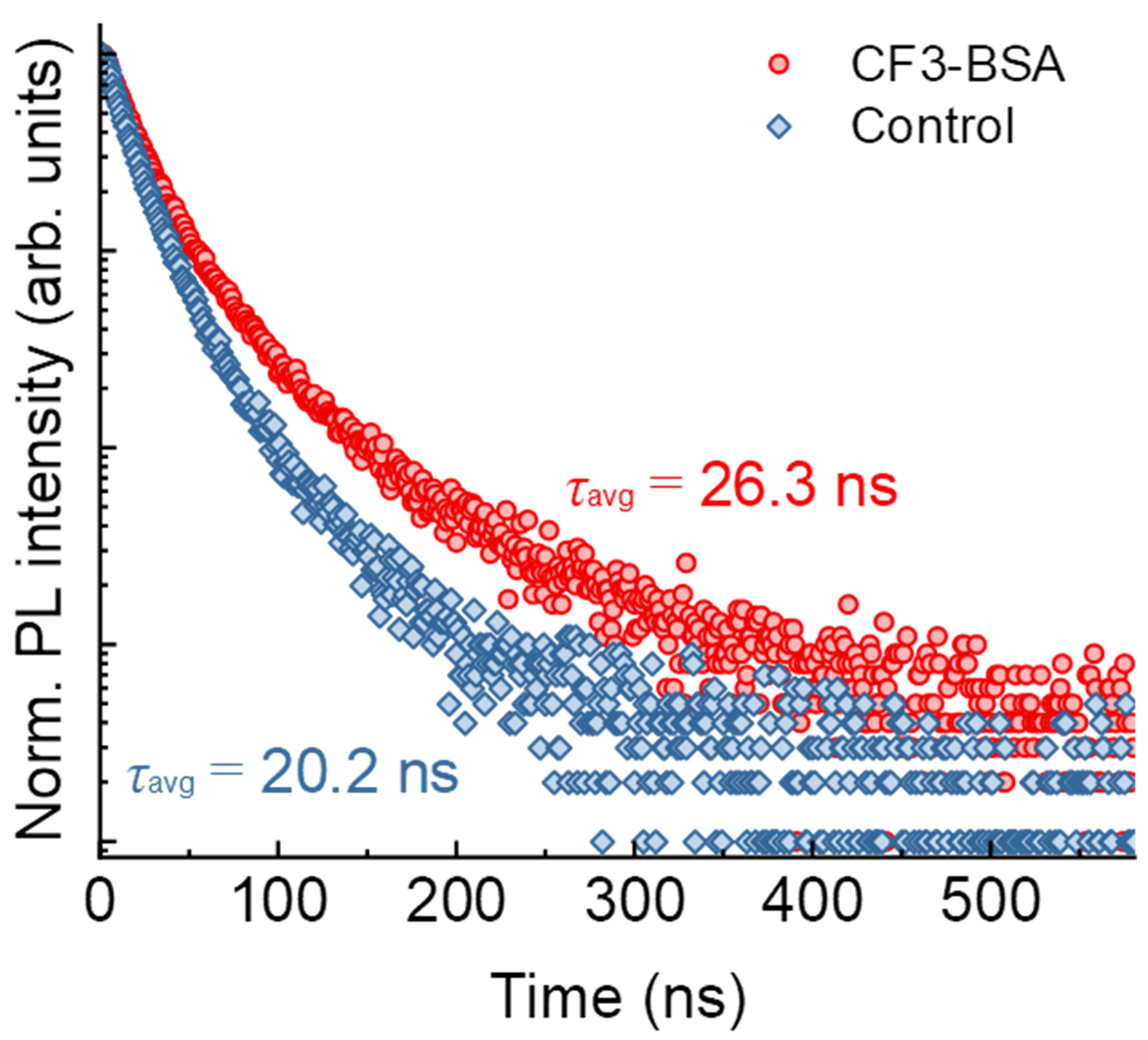


**Supplementary Fig. 13| Average lifetimes of perovskite films.** Time-resolved photoluminescence (TRPL) decay spectra of the control and CF3-BSA based films.


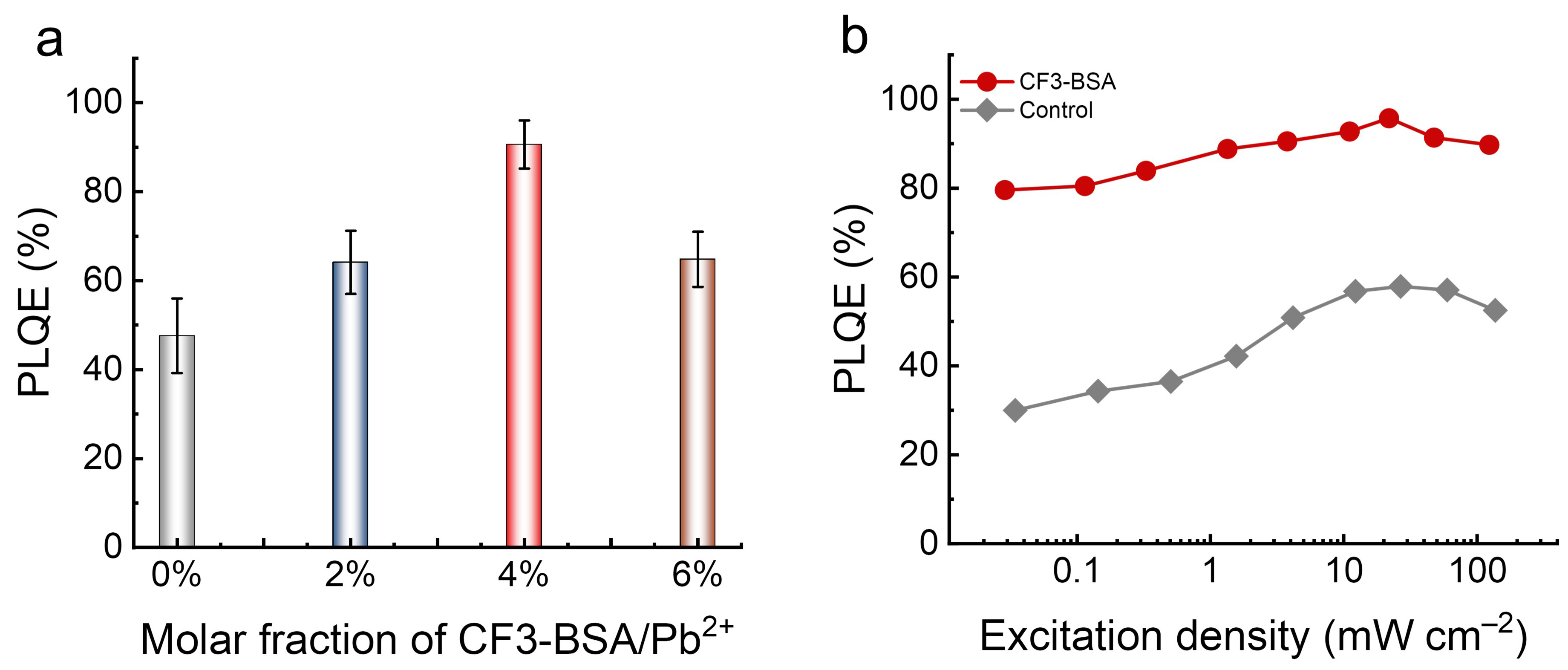


**Supplementary Fig. 14| PLQE of the samples.** **a** PLQE distribution of 15 perovskite films fabricated with various amounts of CF3-BSA additive. The error bars show the highest and lowest EQE values for each molar fraction. The columnar represent the average PLQEs. **b** Excitation-intensity-dependent PLQE of the control and CF3-BSA (4%) based perovskite films.


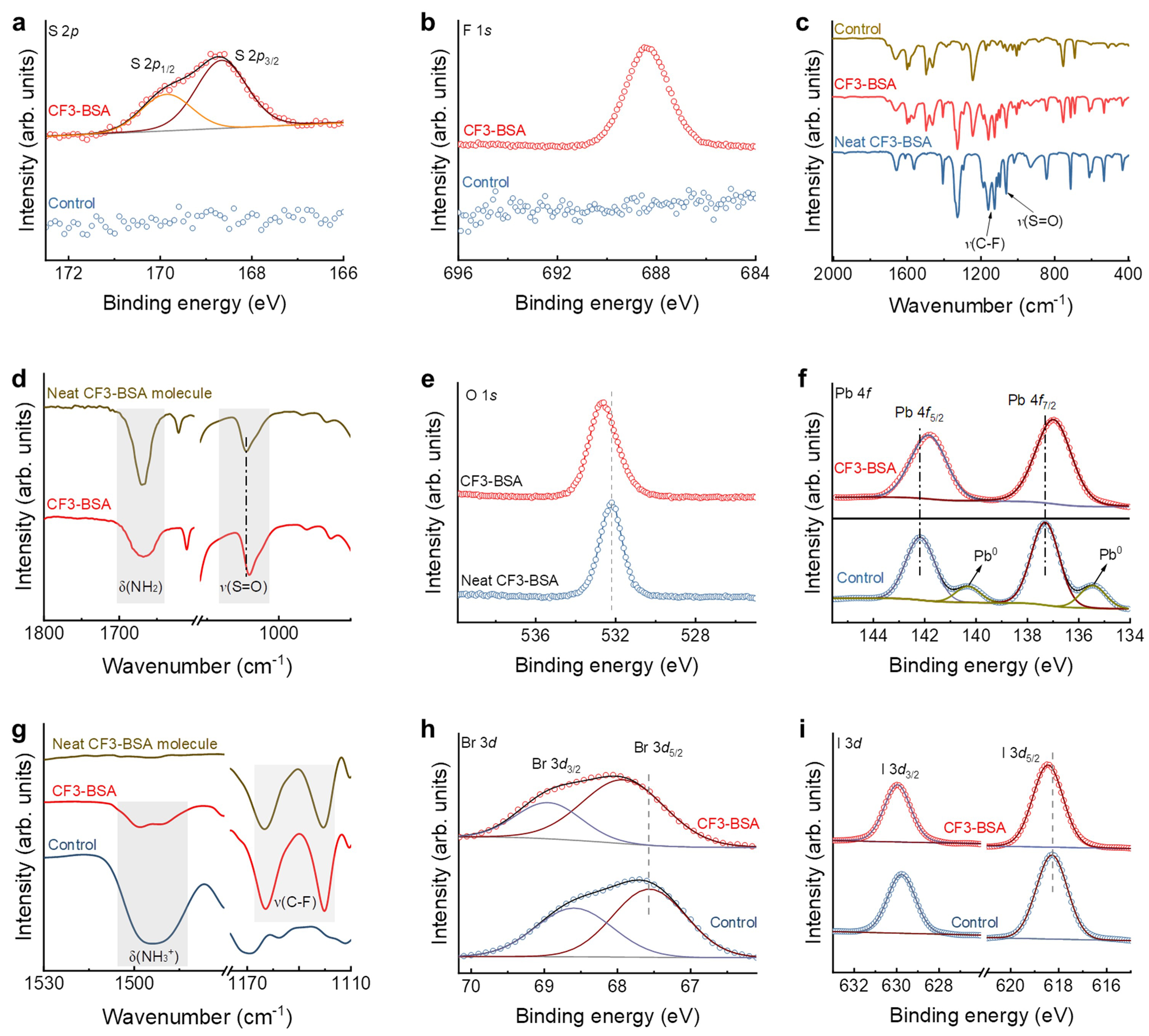


**Supplementary Fig. 15| Interactions between perovskites and CF3-BSA. a** High-resolution XPS spectra of S 2*p* for control and CF3-BSA based films. **b** High-resolution XPS spectra of F 1*s* for control and CF3-BSA based films. **c** FTIR spectra of neat CF3-BSA molecule, control films, and CF3-BSA based films. **d** FTIR spectra of neat CF3-BSA molecule and the CF3-BSA based films. **e** High-resolution XPS spectra of O 1*s* for neat CF3-BSA molecule and CF3-BSA based films. **f** High-resolution XPS of Pb 4*f* for control and CF3-BSA based films. **g** FTIR spectra of neat CF3-BSA molecule, control films, and CF3-BSA based films. **h** High-resolution XPS spectra of Br 3*d* for control and CF3-BSA based films. **i** High-resolution XPS spectra of I 3*d* for control and CF3-BSA based films.


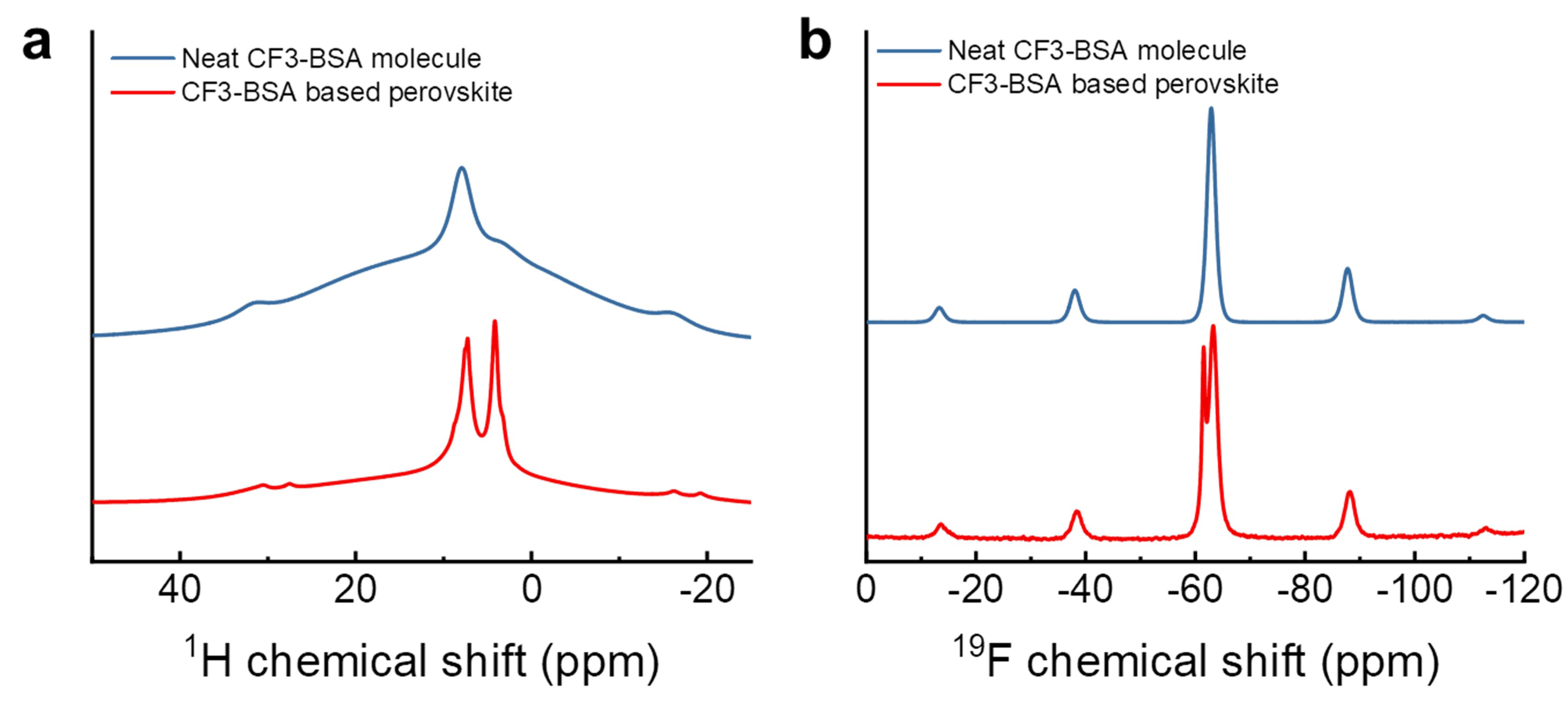


**Supplementary Fig. 16| Solid-state NMR analysis of the films. a** ^1^H and (**b**) ^19^F NMR studies for neat CF3-BSA molecule and CF3-BSA based perovskite films.


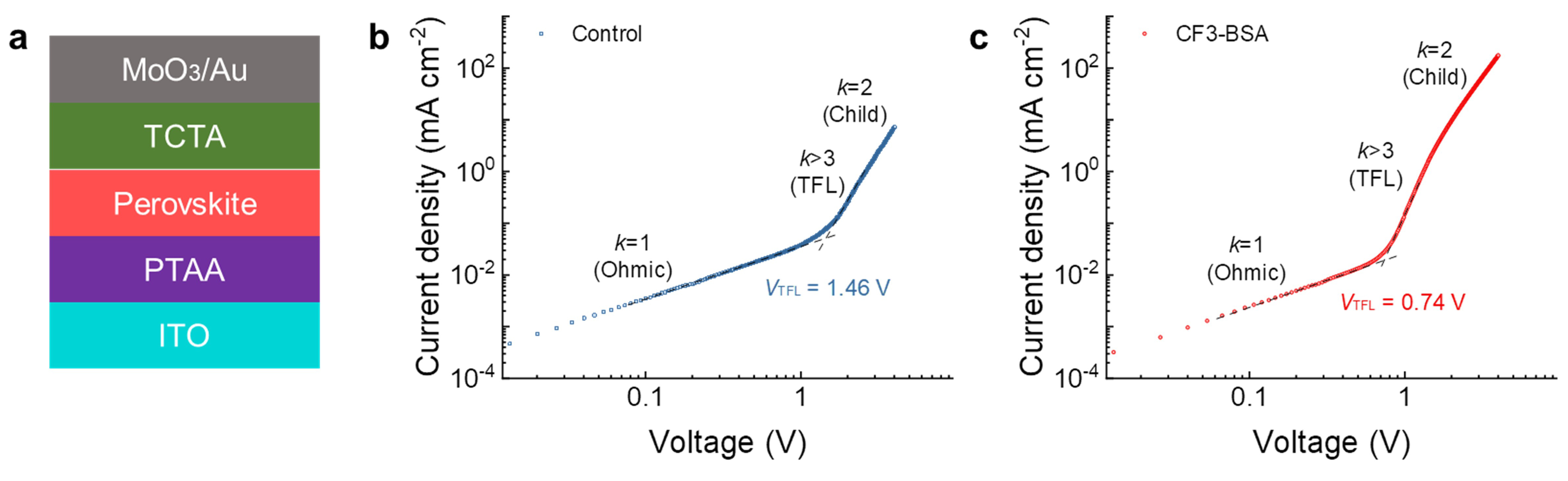


**Supplementary Fig. 17|** **SCLC analysis. a** The structure of hole-only device. Current density *versus* voltage for hole-only devices under dark conditions of control (**b**) and CF3-BSA based films (**c**).


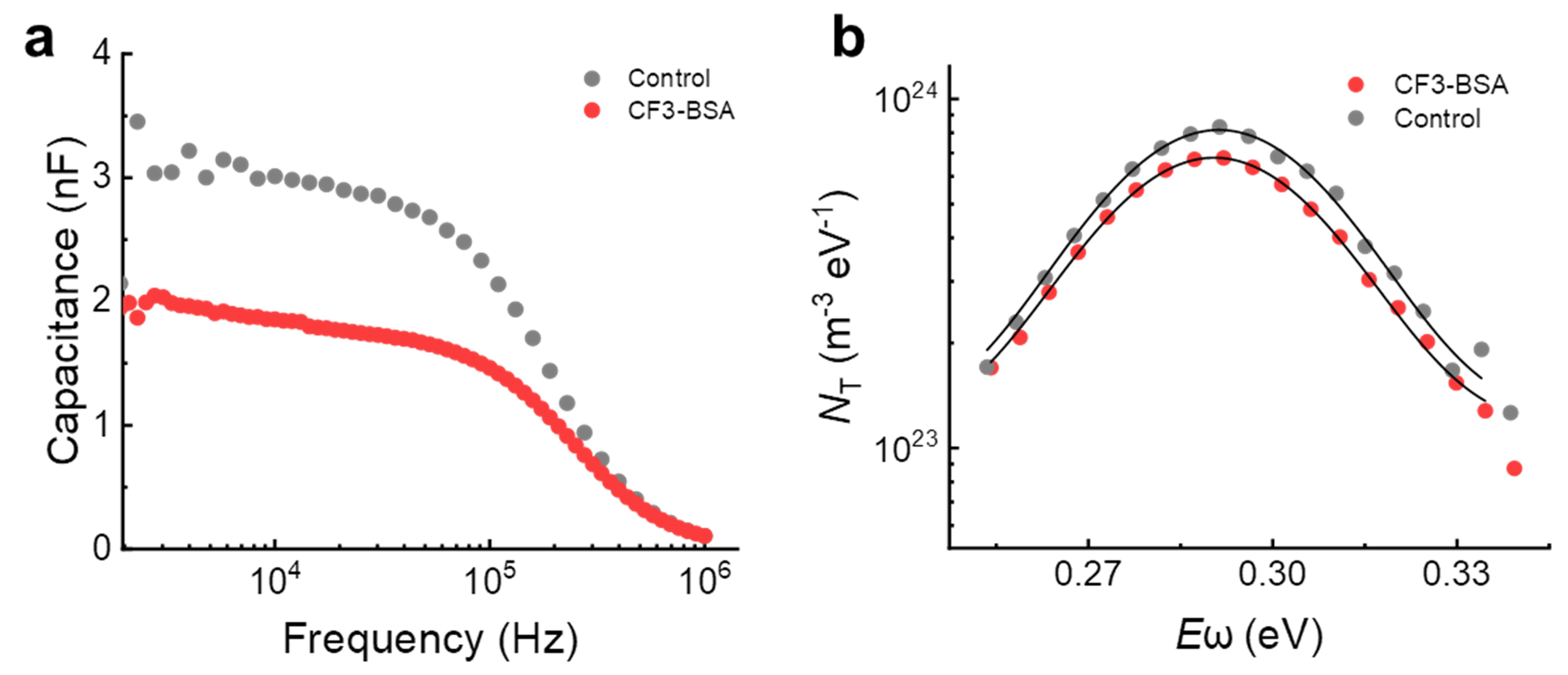


**Supplementary Fig. 18| Trap state distribution of the films. a** Capacitance *versus* frequency characteristics (*c-f*) measured at room temperature. **b** Trap state distributions of perovskites with and without CF3-BSA treatment deduced from the room-temperature *c-f* plots.


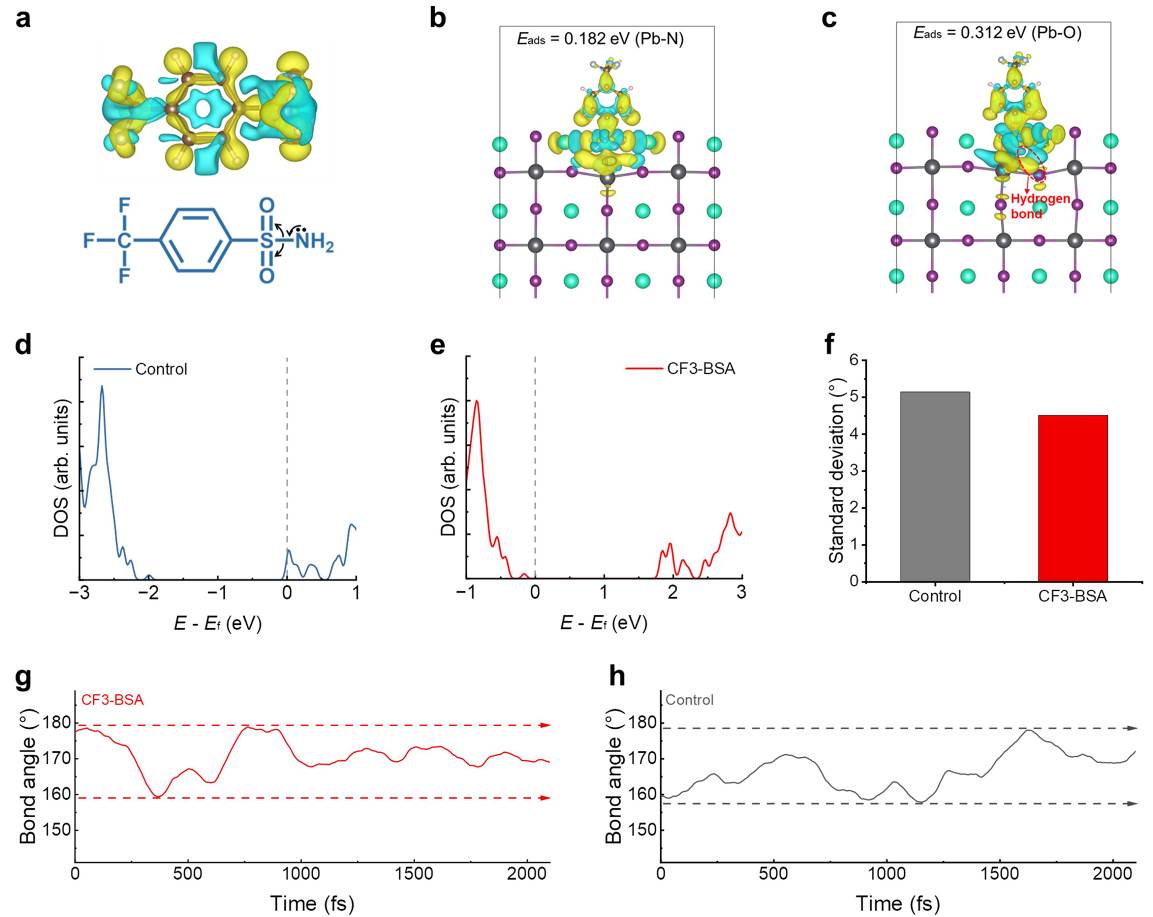


**Supplementary Fig. 19| DFT calculations. a** 3D charge density diﬀerence of CF3-BSA molecule. **b** 3D charge density diﬀerence of uncoordinated Pb^2+^ related defects passivated by -NH_2_ group of the CF3-BSA. **c** 3D charge density diﬀerence of uncoordinated Pb^2+^ related defects passivated by S=O group of the CF3-BSA. **d** DFT calculations reveal trap states of perovskites. **e** DFT calculations reveal trap-free bandgap of perovskites. **f** Standard deviation of I-Pb-I in CsPbI_3_ without and with CF3-BSA passivation. **g,h** Time-dependent bond angles of the samples.


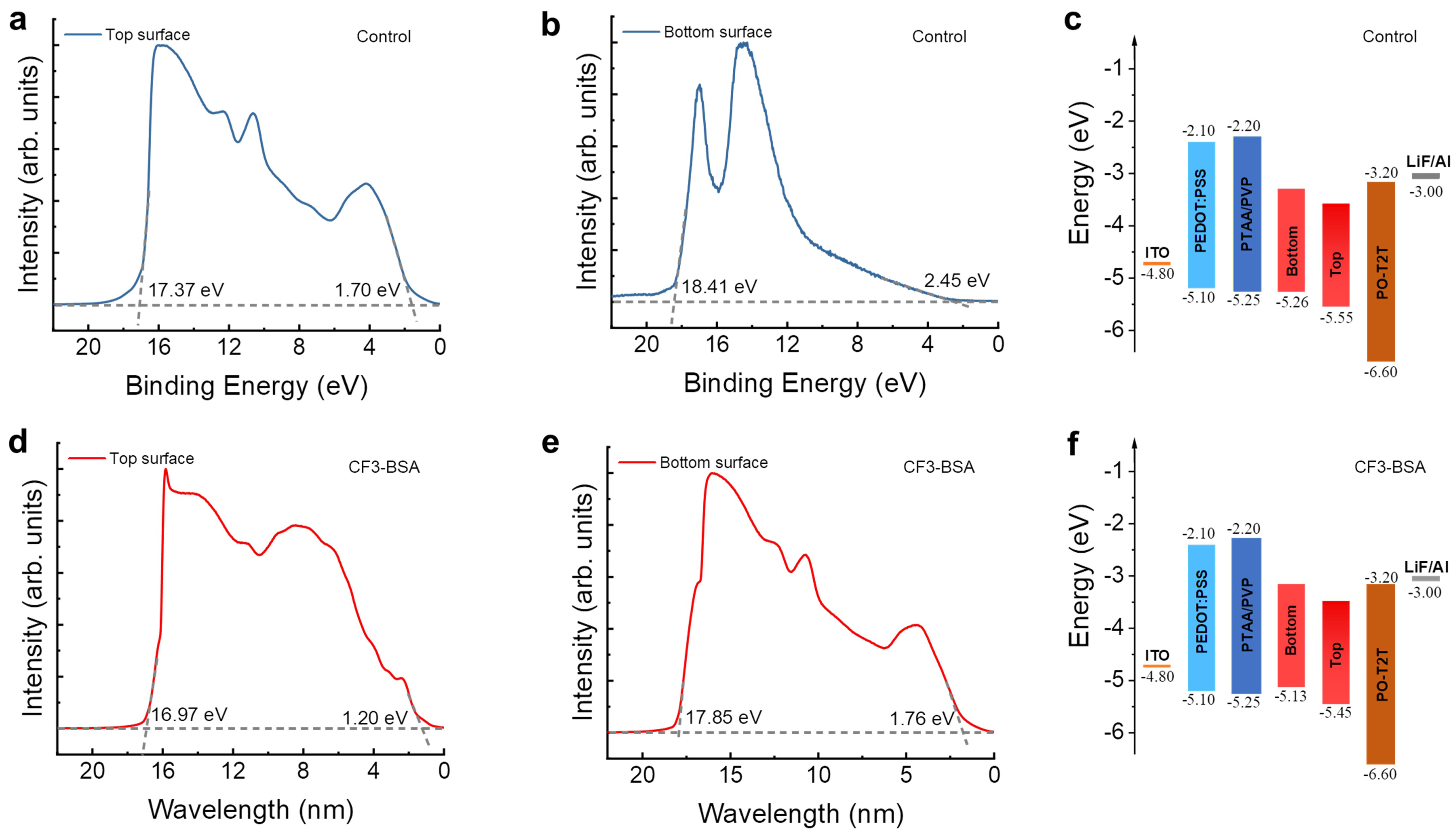


**Supplementary Fig. 20| Energy level analysis of perovskite films.** UPS spectra for top surface and bottom surface of (**a,b**) control and (**d,e**) CF3-BSA based films. Band alignment of each functional layer in (**c**) control and (**f**) CF3-BSA based devices.


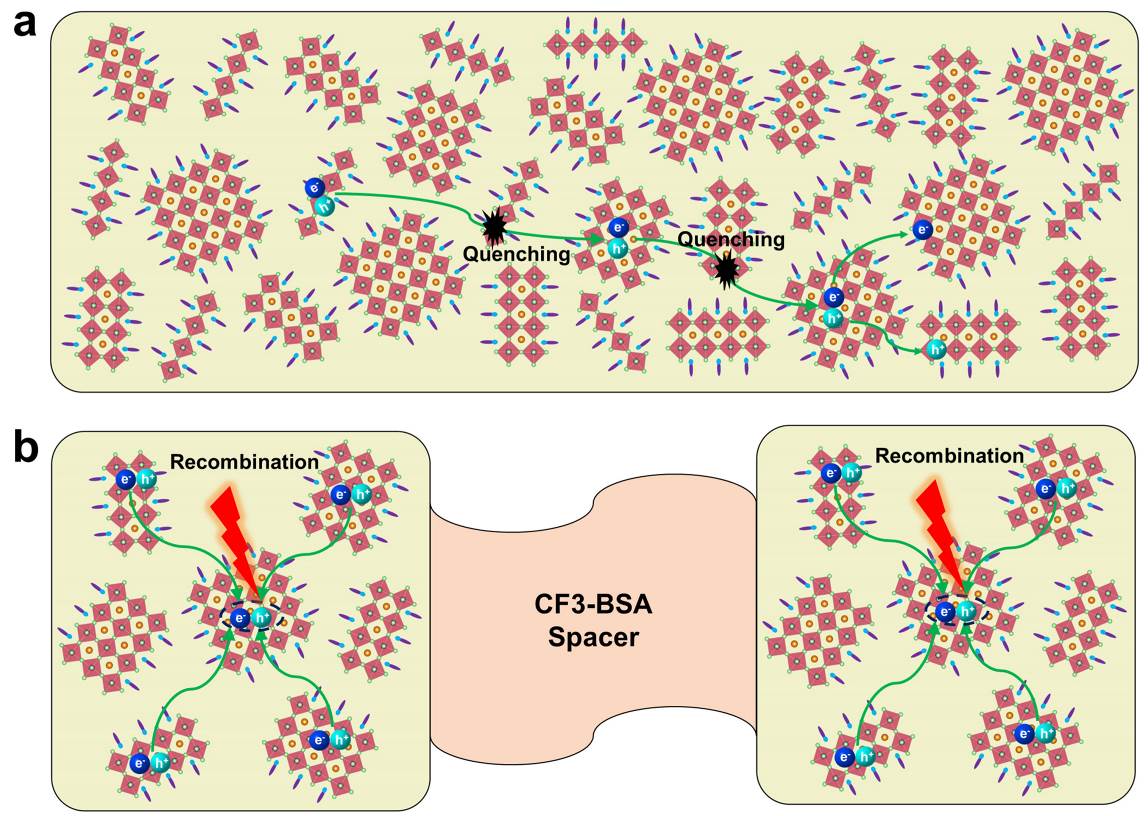


**Supplementary Fig. 21|** **Schematic of CF3-BSA spacer in the films.** Schematic of dimensionality evolutions and corresponding probable exciton quenching and recombination processes for (**a**) control and (**b**) CF3-BSA based films.


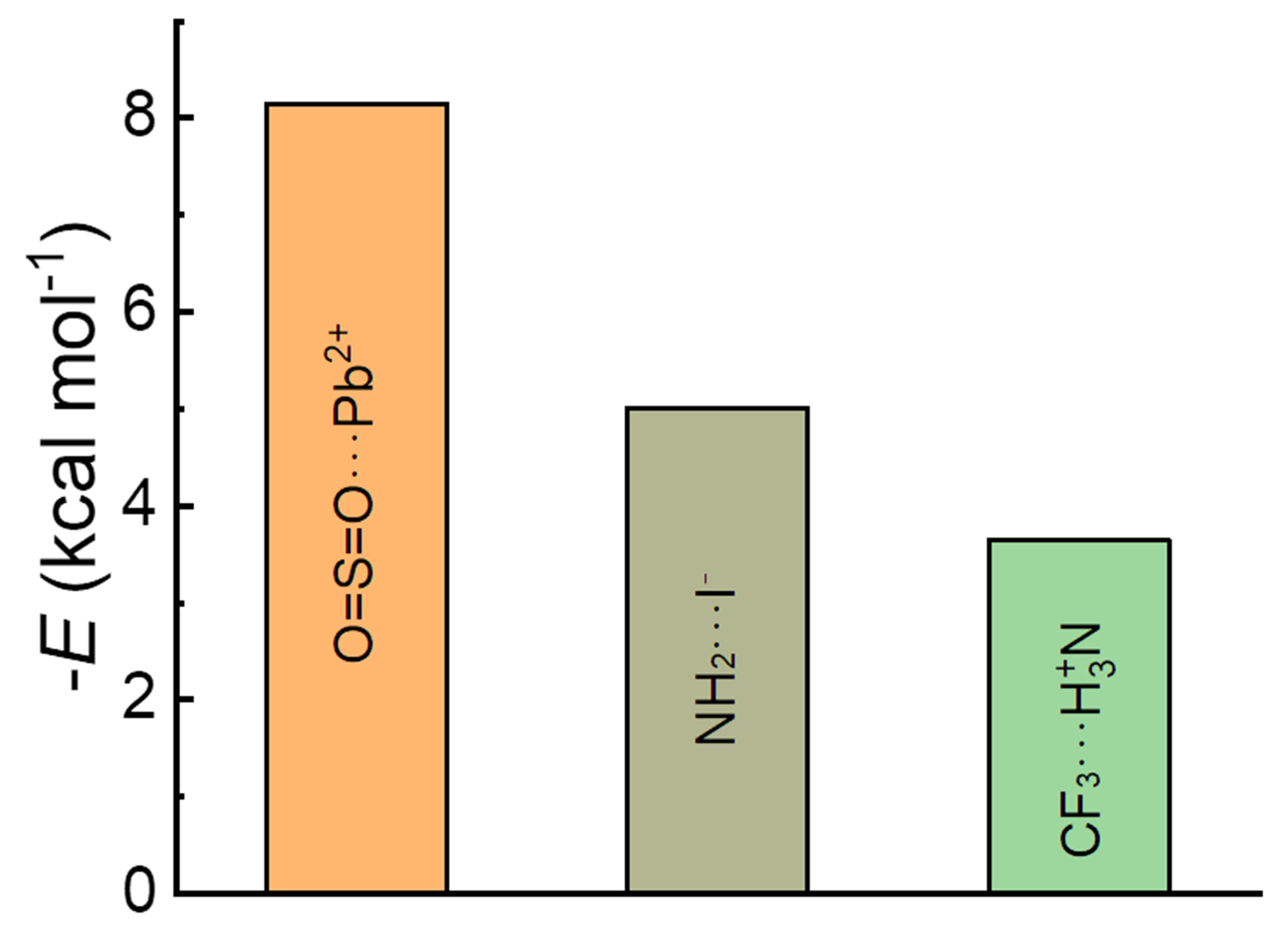


**Supplementary Fig. 22|** **Interaction energies of CF3-BSA and precursors.** The interaction energies of functional groups in CF3-BSA with Pb^2+^, I^-^ and NH_3_^+^.


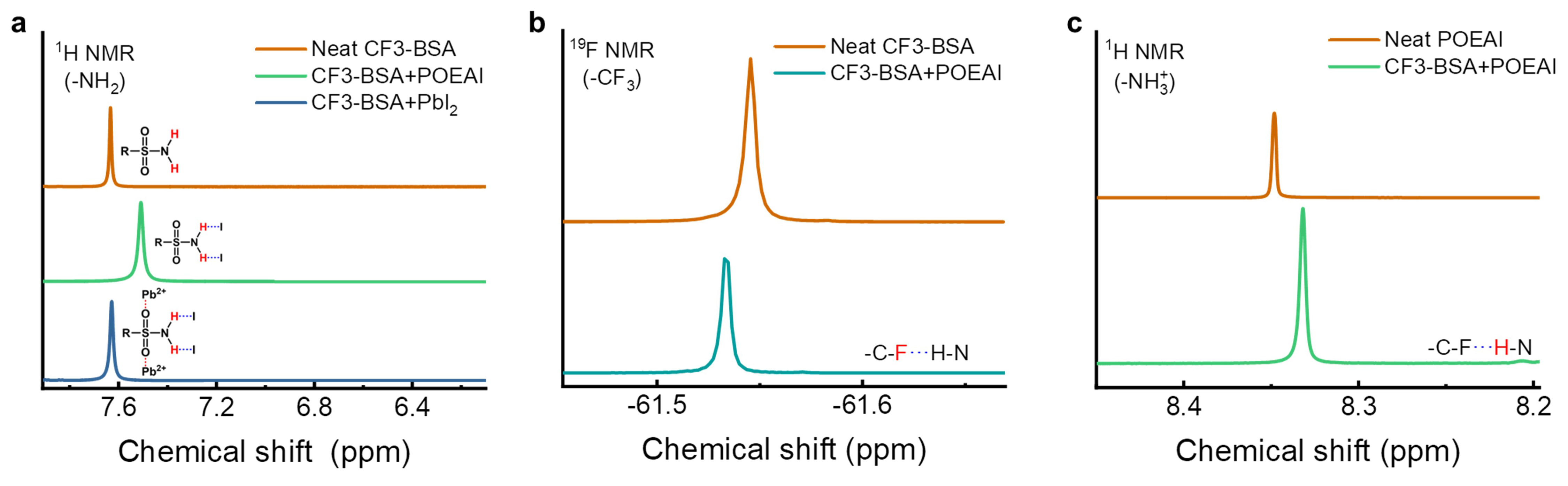


**Supplementary Fig. 23| Interactions between CF3-BSA and perovskite precursors. a** ^1^H NMR of neat CF3-BSA molecule, CF3-BSA+POEAI, and CF3-BSA+PbI_2_. **b** ^19^F NMR of neat CF3-BSA molecule, and CF3-BSA+POEAI. **c** ^1^H NMR of neat POEAI and CF3-BSA+POEAI.


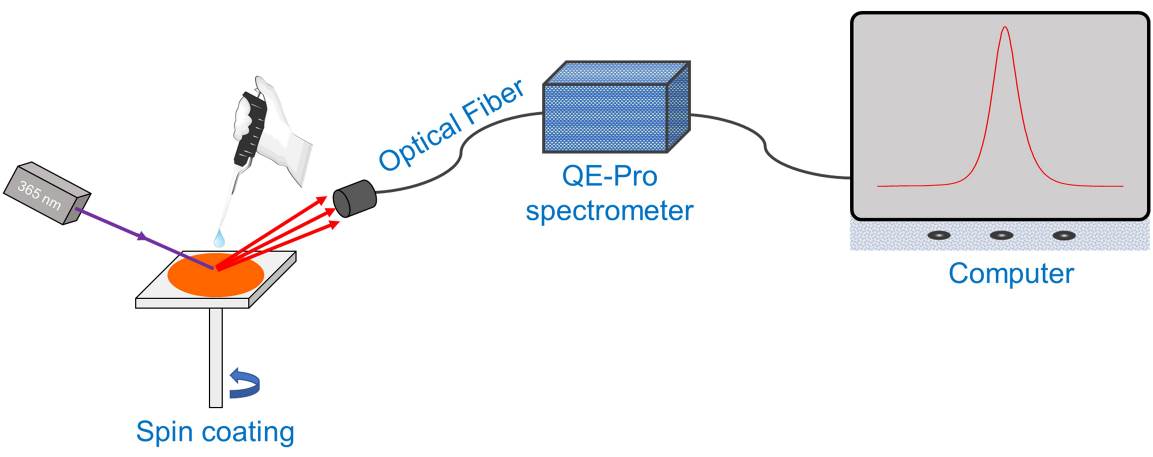


**Supplementary Fig. 24****| Schematic diagram of the in situ spectroscopic methods.**


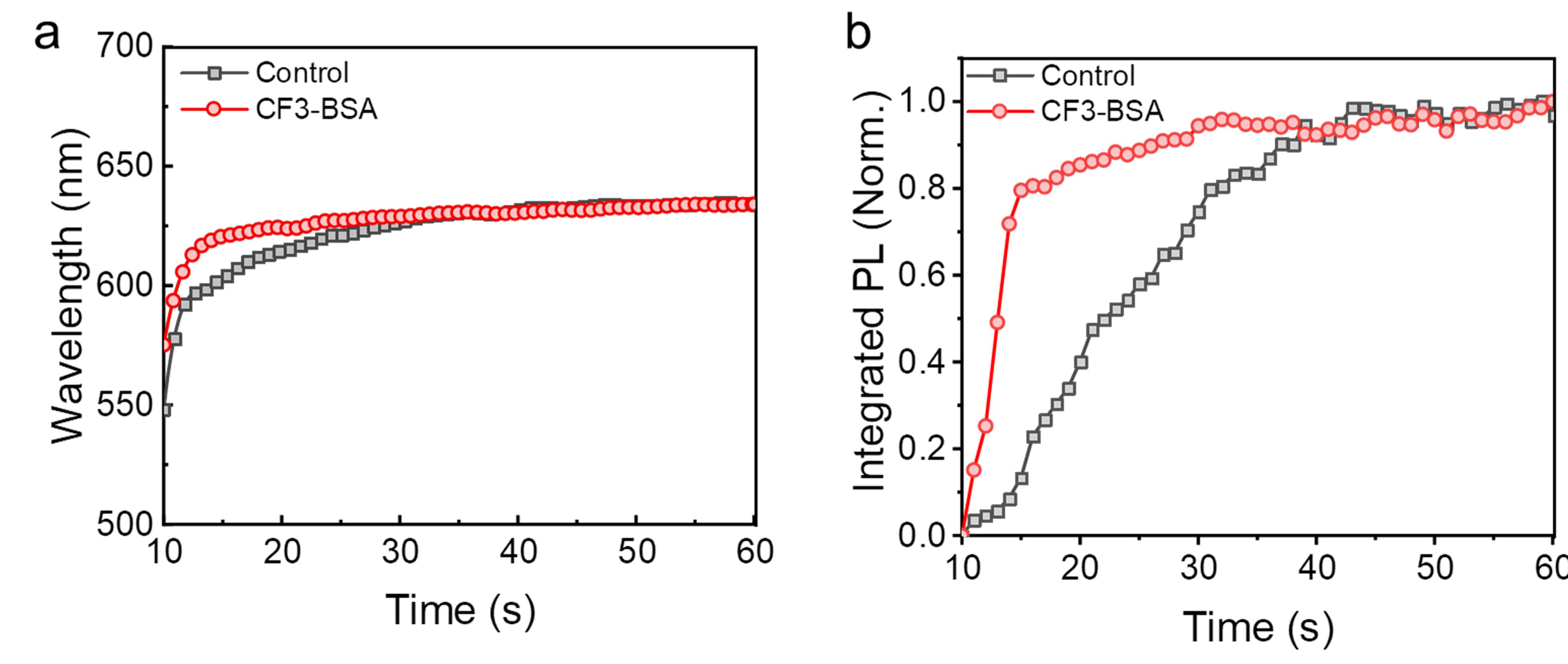


**Supplementary Fig. 25| In situ analysis of perovskite film crystallization.** The evolution of (**a**) PL peak position and (**b**) normalized PL intensity for the control and CF3-BSA modified films.


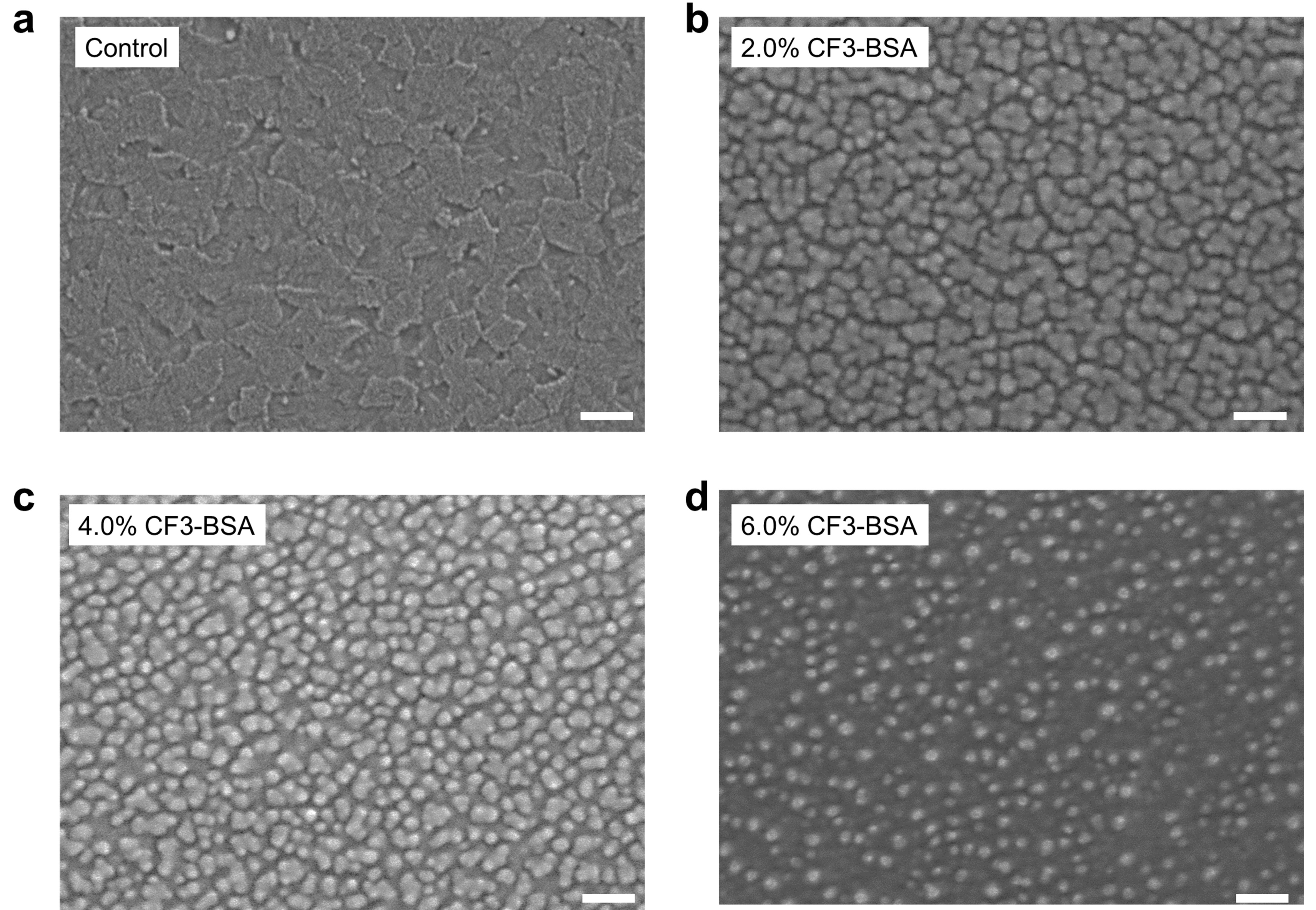


**Supplementary Fig. 26| Morphology characteristic of the provskite films.** SEM images of the perovsktie films with different fraction of CF3-BSA (scale bar: 50 nm).


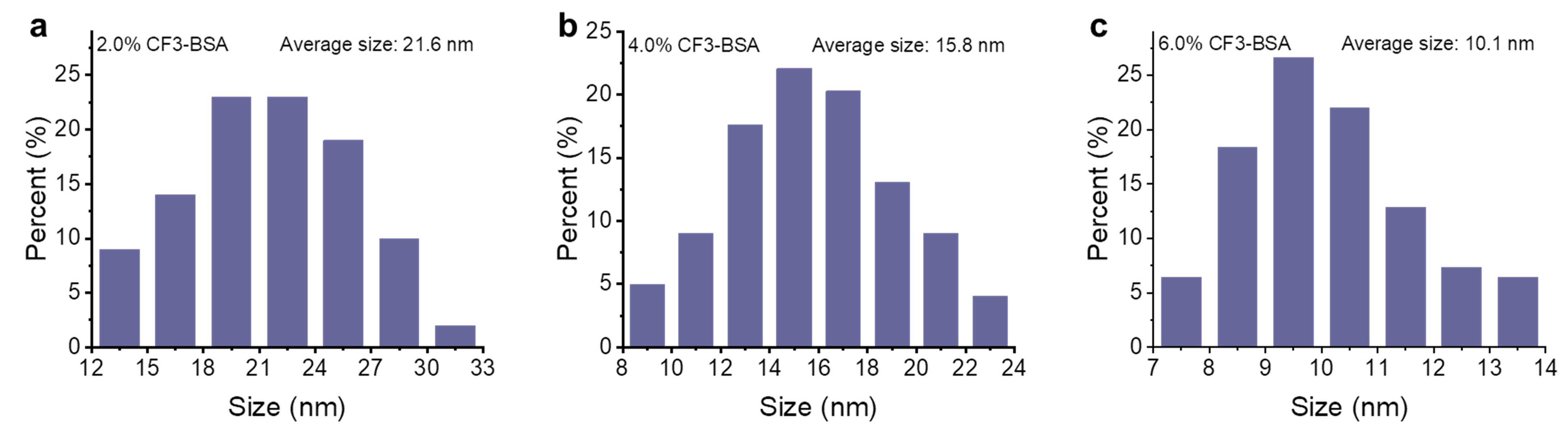


**Supplementary Fig. 27| Size distribution of the films.** Percent from the different size of quasi‑2D perovsktie nanocrystals with different fraction of CF3-BSA to (PbI_2_+PbBr_2_). **a** 2%. **b** 4%. **c** 6.0%.


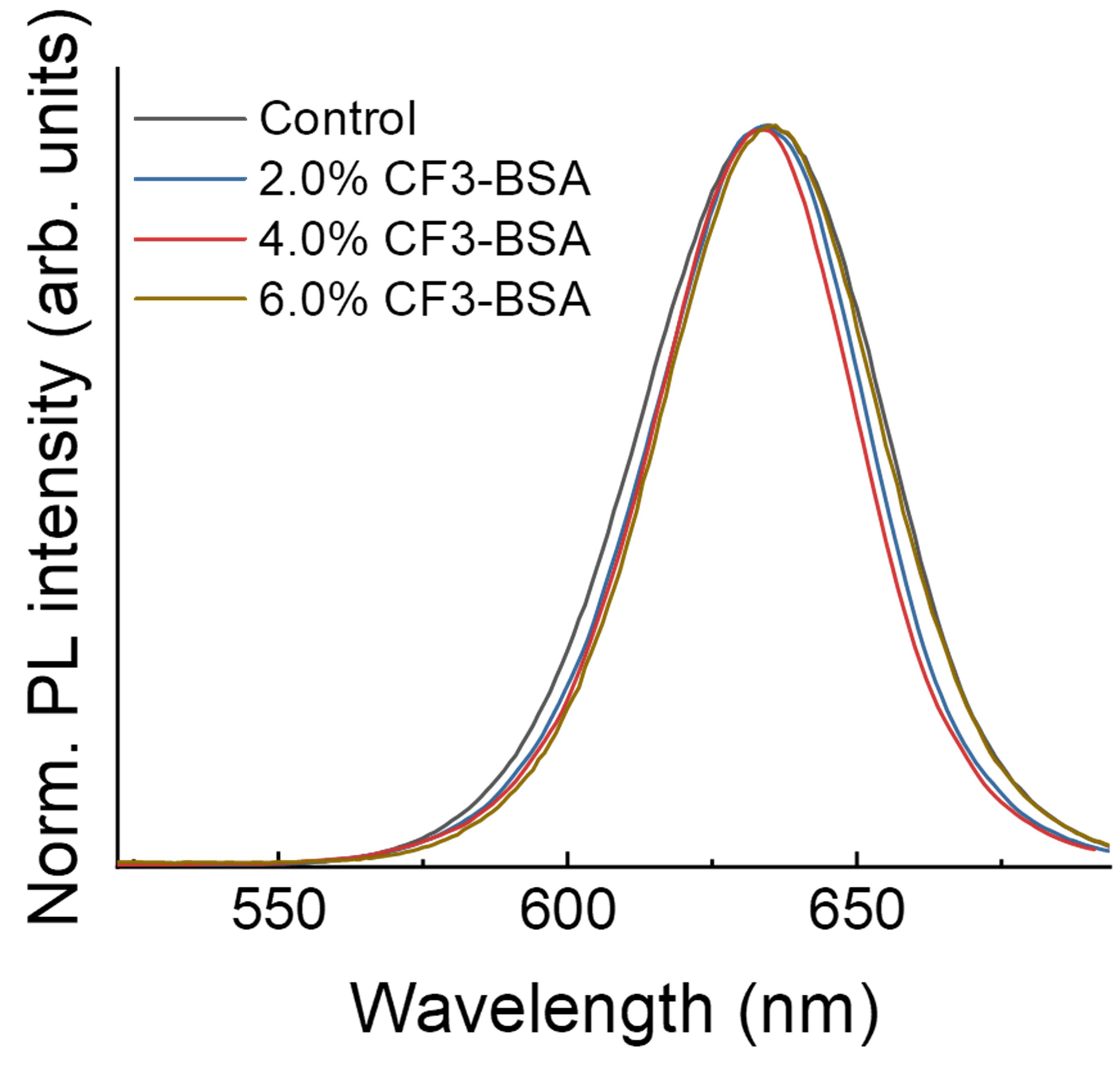


**Supplementary Fig. 28| Normailized PL intensity of the perovskite films.** Luminescent properties of the quasi‑2D perovskties incorporated with different fraction CF3-BSA of 0%, 2.0%, 4.0%, and 6.0%.


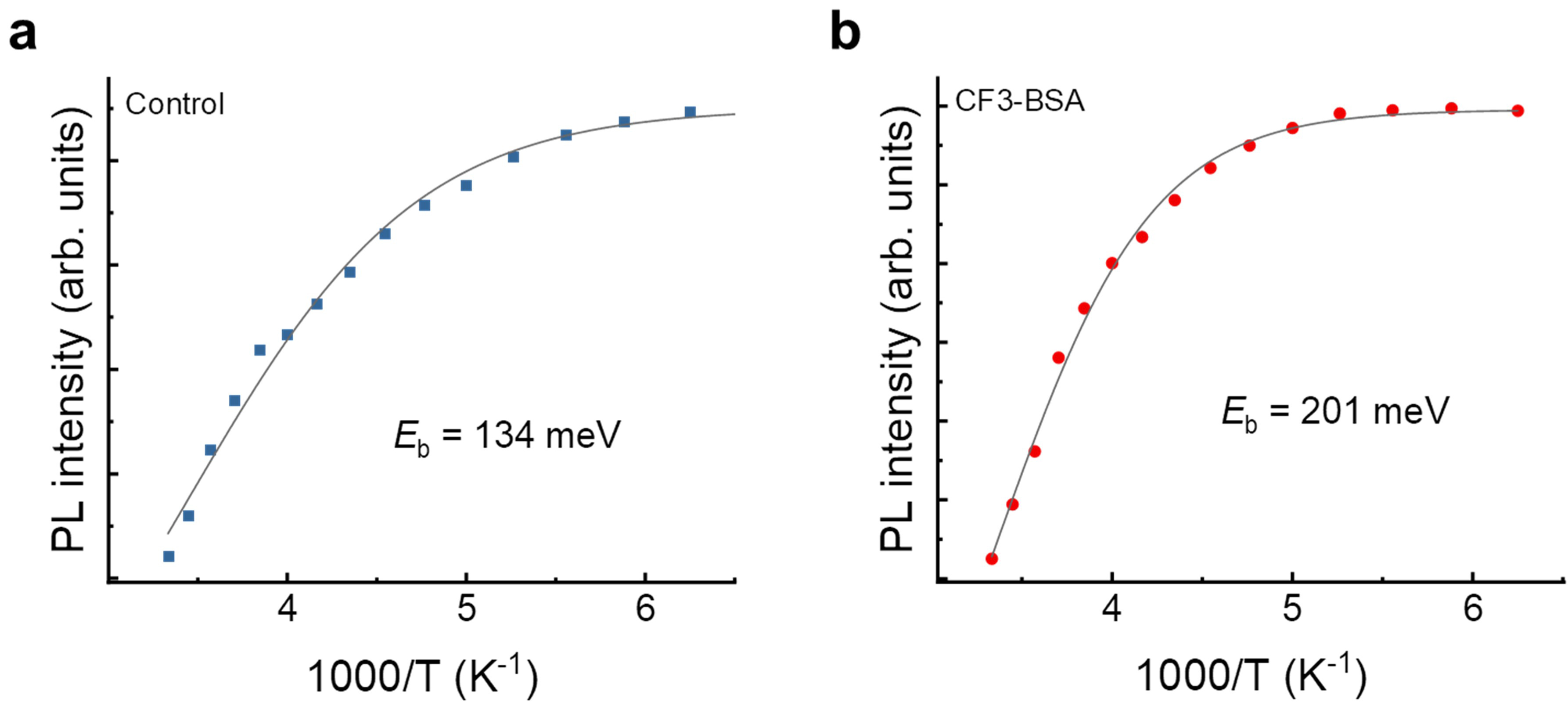


**Supplementary Fig. 29| Exciton binding energy of the perovskites.** Integrated PL intensity of (**a**) control and (**b**) CF3-BSA based perovskites as a function of reciprocal temperature.


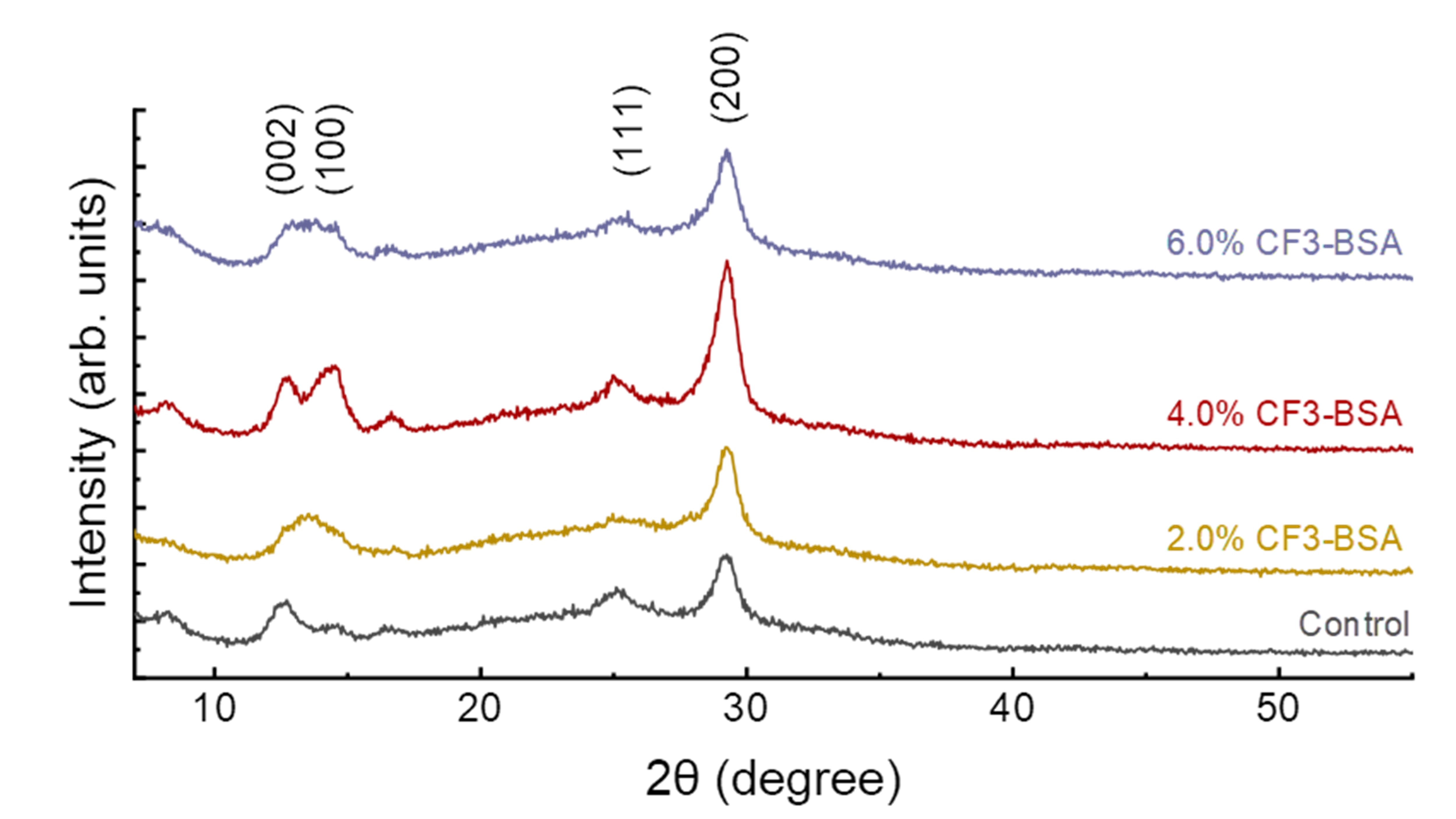


**Supplementary Fig. 30|** **Structural characteristics of the films.** XRD patterns of the perovskite films with different fraction of CF3-BSA.


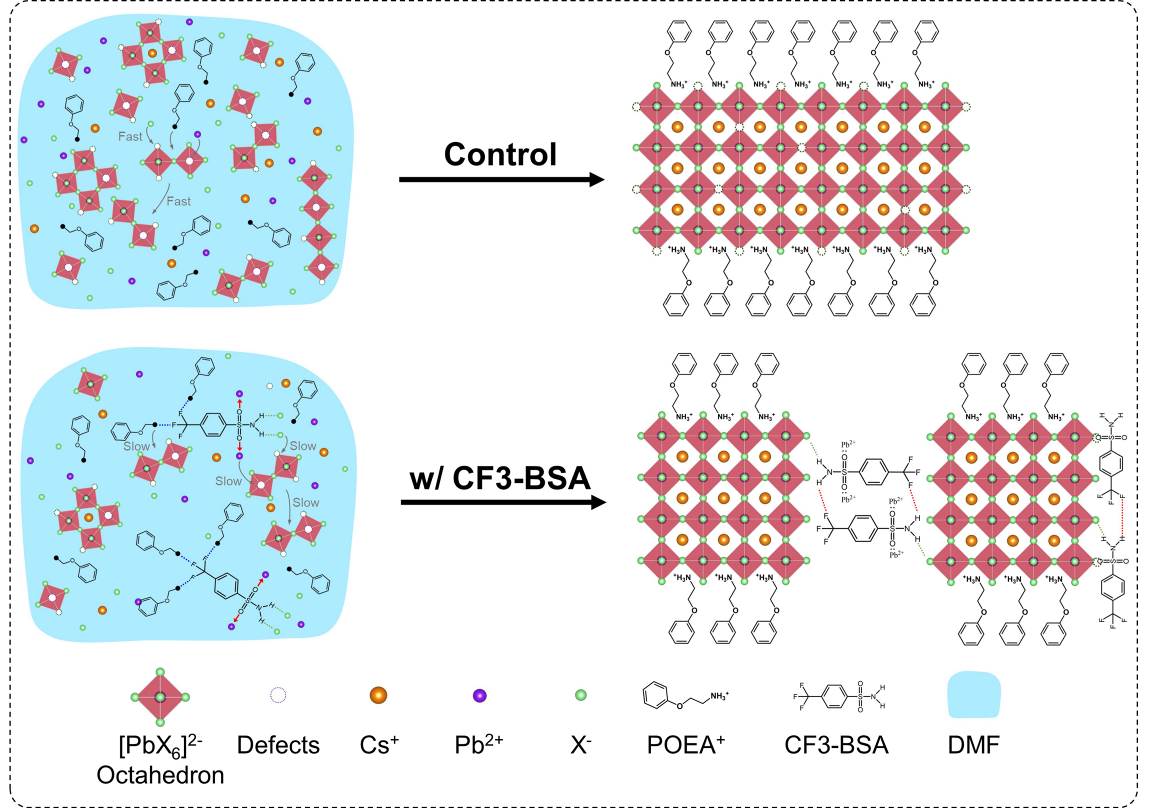


**Supplementary Fig. 31| Perovskites growth control strategy.** The interaction between CF3-BSA and perovsktie precursors during the film growth.


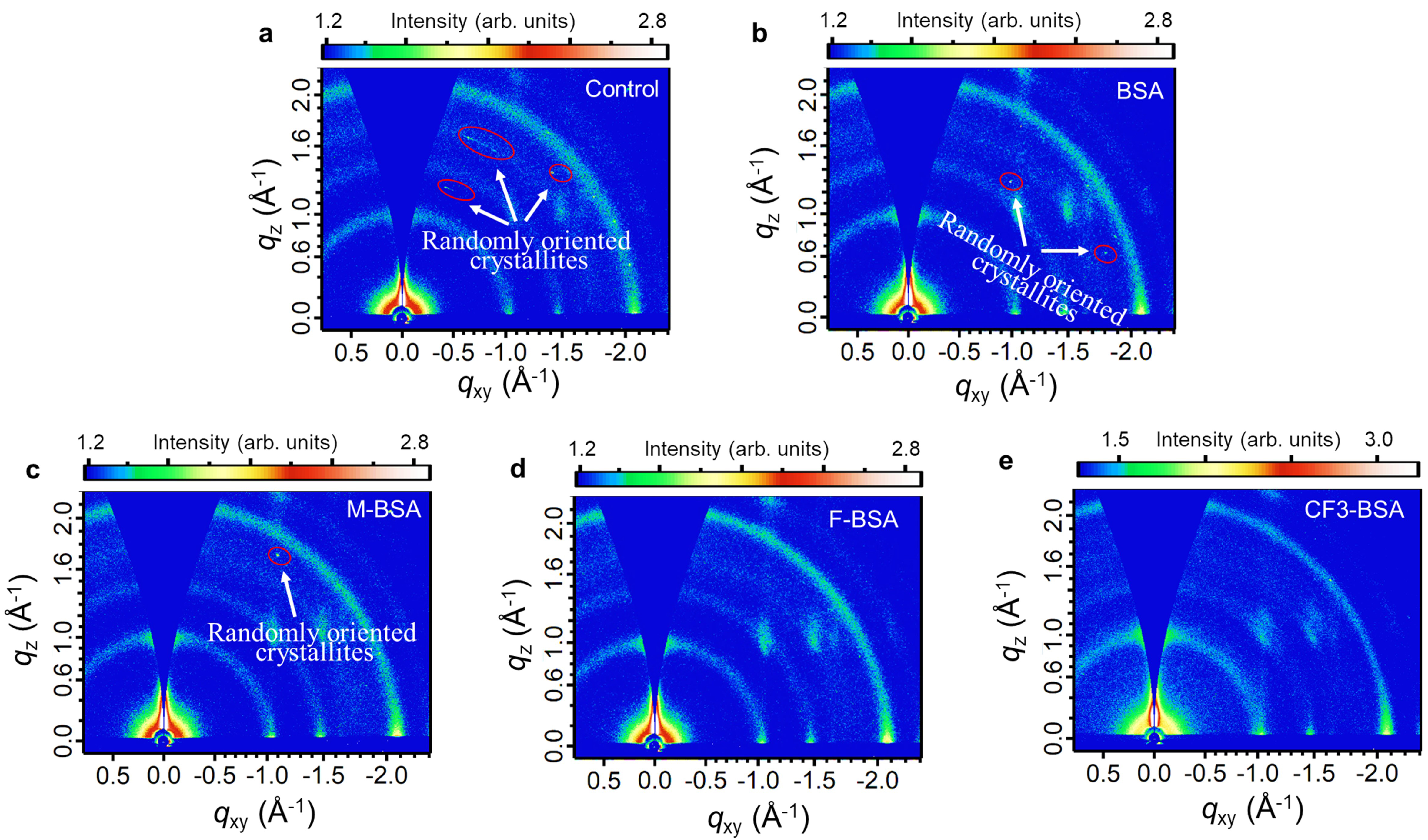


**Supplementary Fig. 32| Analysis of oriented growth in perovskite films.** GIWAXS of the perovskite films incorporated with different additives. **a** Control film. **b** Incorporated with BSA. **c** Incorporated with M-BSA. **d** Incorporated with F-BSA. **e** Incorporated with CF3-BSA.


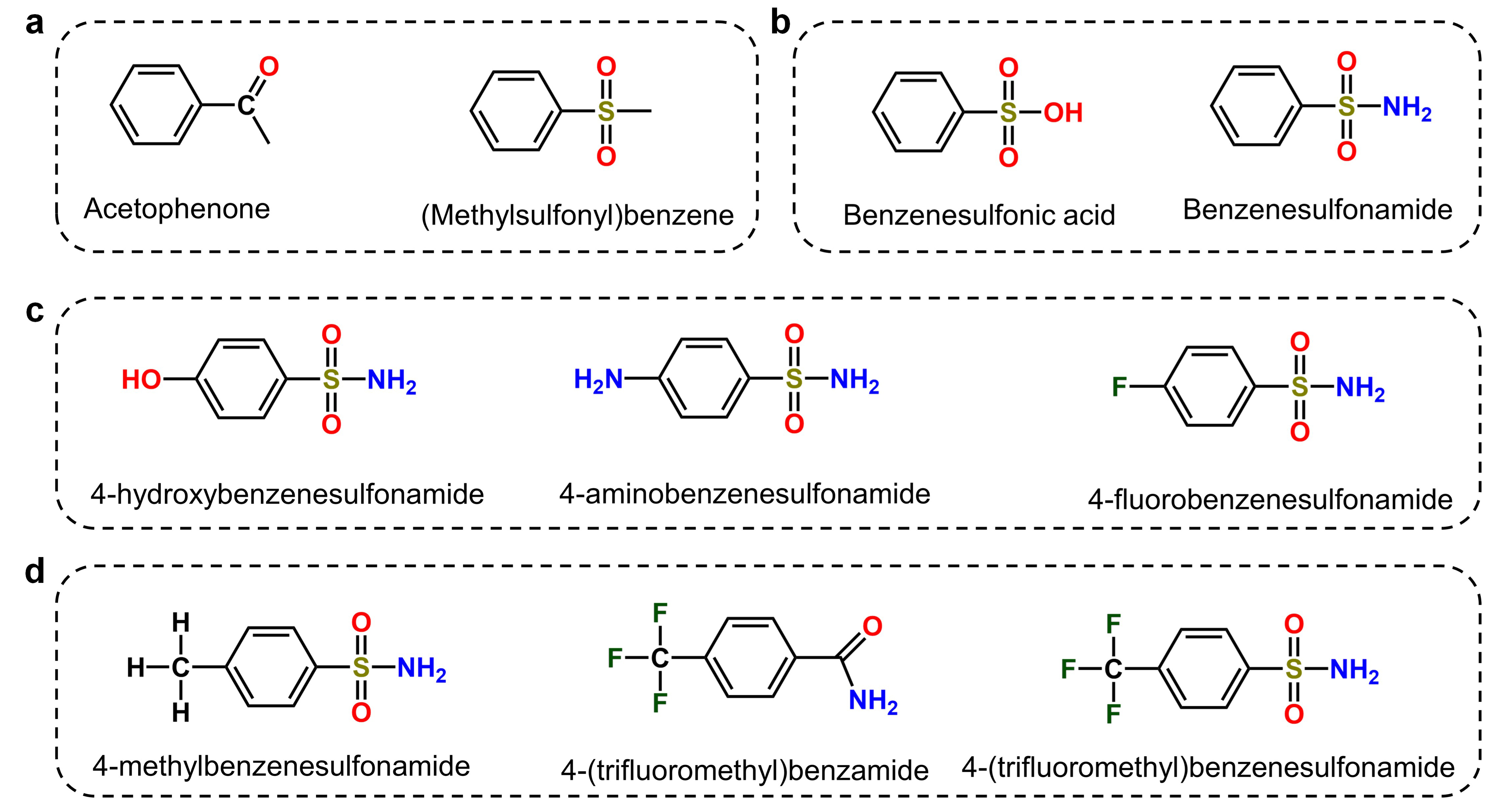


**Supplementary Fig. 33| Structures of the selected additives. a** Molecule with only C=O and S=O passivating groups. **b** Molecule with halide-bonding groups near the passivating groups. **c** Molecule with organic cation binding groups opposite the passivating groups. **d** Bulky sterically hindered molecule used during perovskite film deposition.


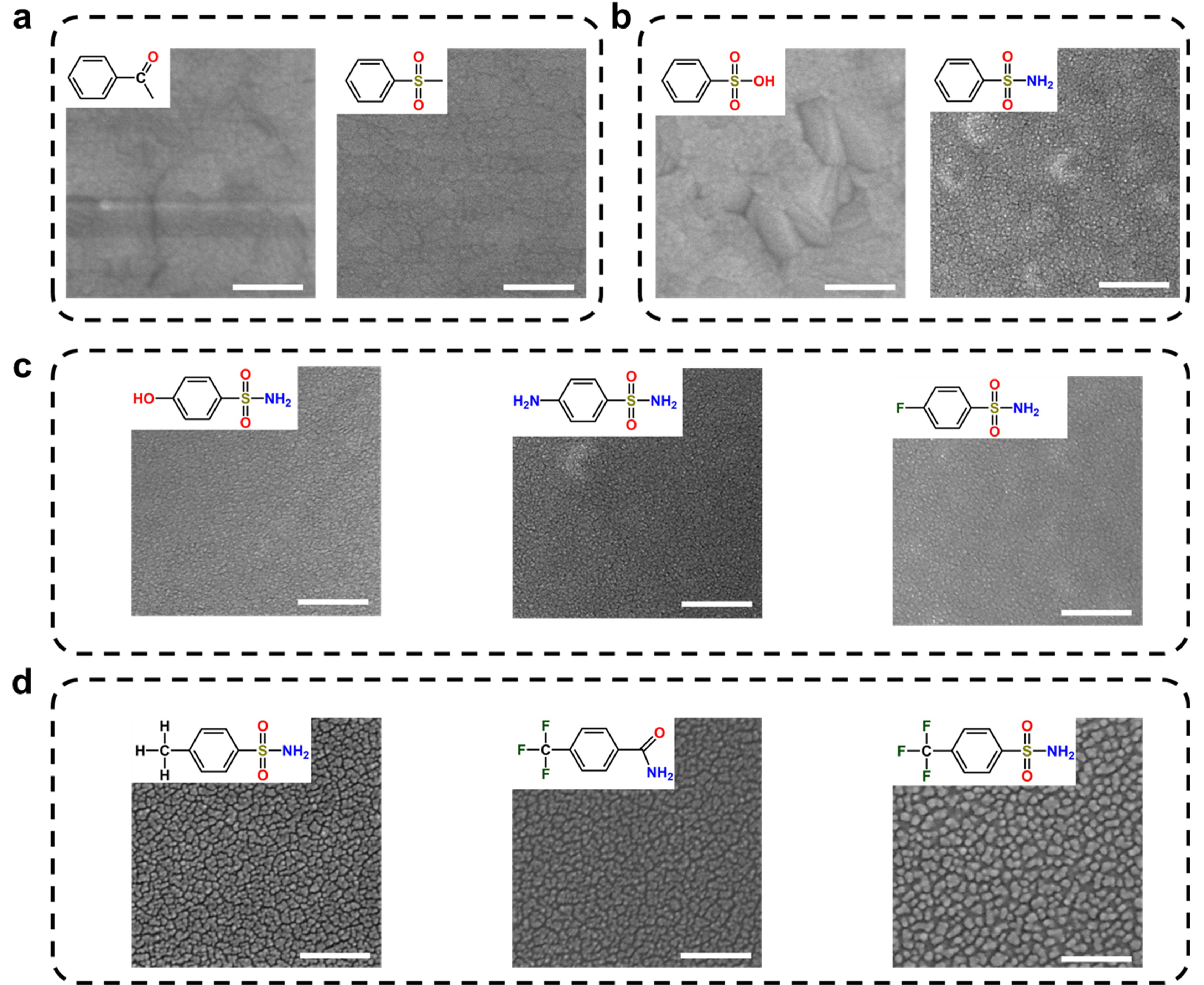


**Supplementary Fig. 34|** **SEM of the perovskite films with different additives. a** Molecule with only C=O and S=O passivating groups. **b** Molecule with halide-bonding groups near the passivating groups. **c** Molecule with organic cation binding groups opposite the passivating groups. **d** Bulky sterically hindered molecule used during perovskite film deposition (scale bar: 100 nm).


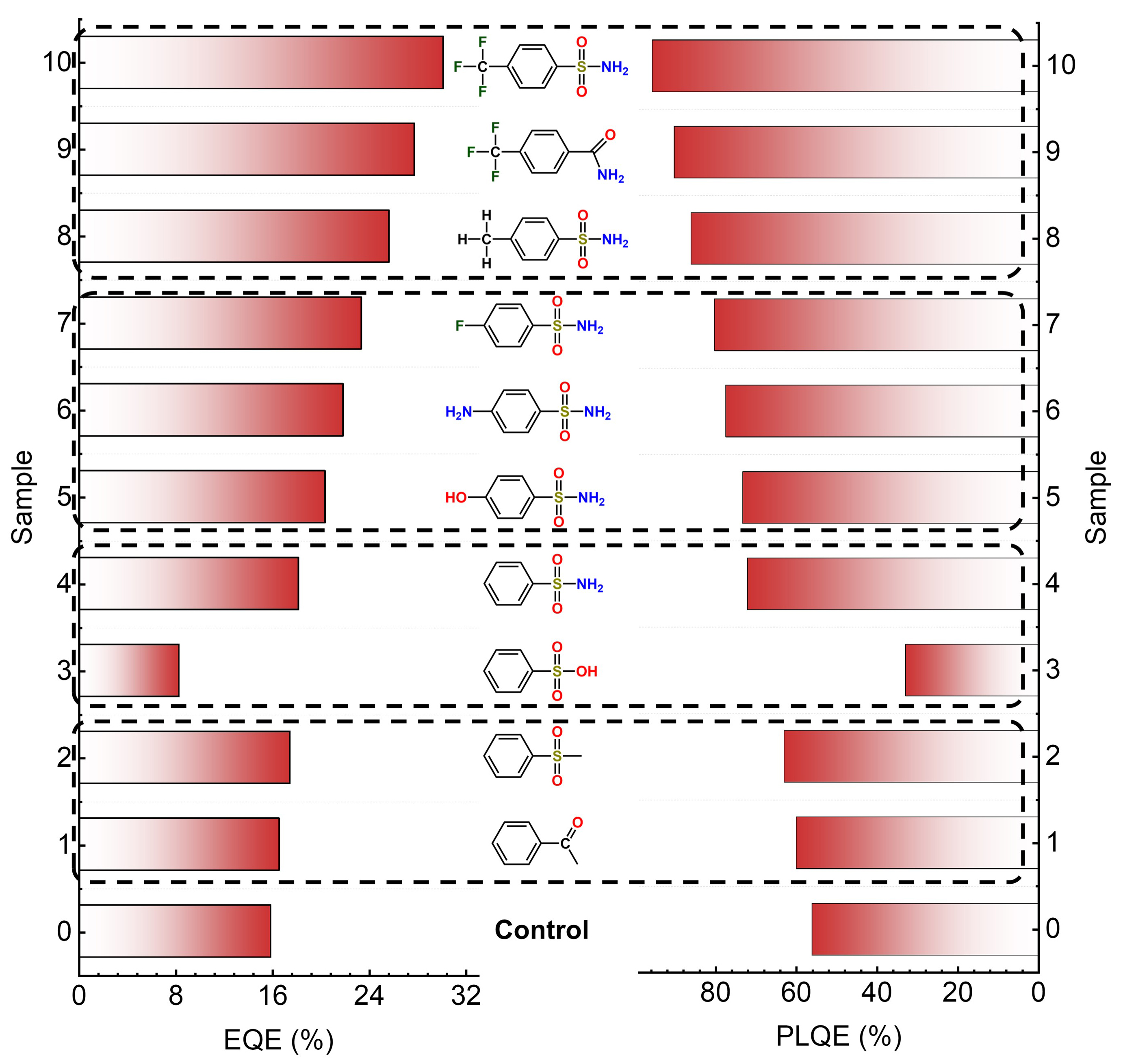


**Supplementary Fig. 35| PL and EL properties of perovskite films.** PLQEs of perovskite films and corresponding EQEs of devices based on different additives.


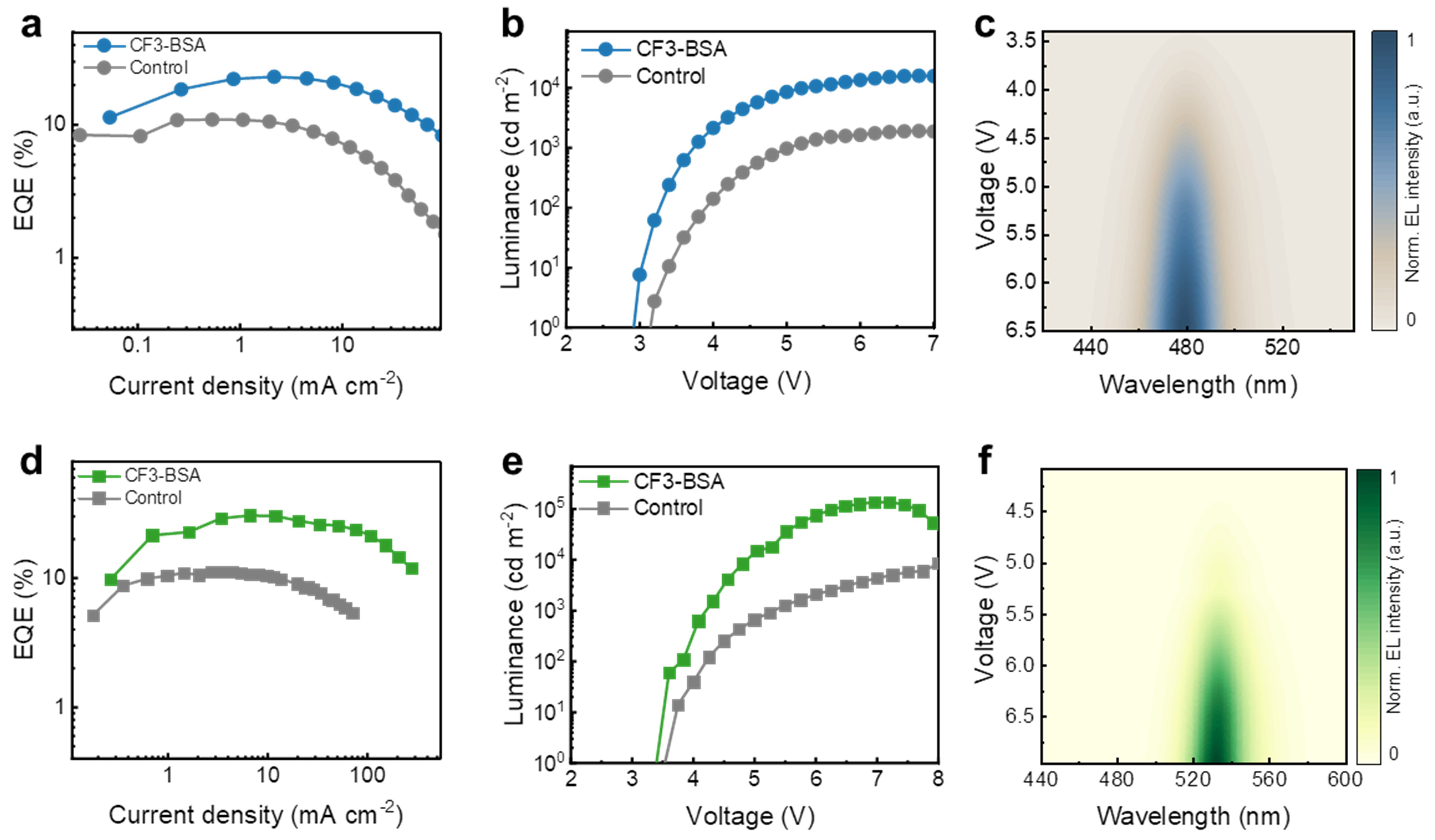


**Supplementary Fig. 36|** **Performance of sky-blue and green PeLEDs. a** Current density-EQE curves and (**b**) voltage-luminance curves of control and CF3-BSA based sky-blue PeLEDs. **c** EL spectra for sky-blue PeLEDs at diﬀerent operating voltages. **d** Current density-EQE curves and (**e**) voltage-luminance curves of control and CF3-BSA based green PeLEDs. **f** EL spectra for green PeLEDs at diﬀerent operating voltages.

**Supplementary Table 1.** Summary of device performances for recently reported pure-red PeLEDs (emitting at 630-640 nm).

| Emitters | EL peak (nm) | EQE  (%) | Max. *L*.  (cd m^-2^) | T_50_  (min) | Ref |
| --- | --- | --- | --- | --- | --- |
| **Quasi-2D Perovskites NCs** | **635** | **30.2** | **25,133** | **8,426** | **This work** |
| Quasi-2D Perovskite films | 638 | 28.7 | 2,800 | 5,730 | 1 |
| Colloidal CsPbI_3_ NCs | 638 | 26.1 | 2,511 | 450 | 2 |
| CsPbI_3_ NCs films | 630 | 24.6 | 11,689 | 6,330 | 3 |
| 3D CsPb(Br/I)_3_ films | 638 | 24.2 | 24,600 | 7,620 | 4 |
| Colloidal CsPb(Br/I)_3_ NCs | 631 | 23.2 | 3,121 | 44.7 | 5 |
| Colloidal CsPb(Br/I)_3_ NCs | 640 | 23.5 | 1,510 | 97 | 6 |
| Colloidal CsPbI_3_ NCs | 640 | 23.0 | 2,000 | 600 | 7 |
| Colloidal CsPb(Br/I)_3_ NCs | 635 | 22.8 | 12,910 | 63 | 8 |
| 3D CsPb(Br/I)_3_ films | 640 | 22.8 | 12,000 | 4,080 | 9 |
| Colloidal CsPbI_3_ NCs | 636 | 22.0 | 7,000 | 46,800 | 10 |
| Colloidal CsPb(Br/I)_3_ NCs | 630 | 21.8 | 21,590 | 702 | 11 |
| Colloidal CsPb(Br/I)_3_ NCs | 637 | 21.8 | 2,653 | 70 | 12 |
| Colloidal CsPb(Br/I)_3_ NCs | 636 | 21.2 | 1,631 | 240 | 13 |
| Colloidal CsPbI_3_ NCs | 636 | 20.8 | 3,775 | 8 | 14 |
| Colloidal CsPbI_3_ NCs | 633 | 20.1 | 4,932 | 145 | 15 |
| Quasi-2D perovskite films | 630 | 18.3% | 2,410 | 540 | 16 |
| 3D CsPb(Br/I)_3_ films | 638 | 17.8% | 9,000 | 40 | 17 |
| Colloidal CsPb(Br/I)_3_ NCs | 648 | 13.2% | 11,233 | NA | 18 |
| Quasi-2D Perovskite films | 635 | 12.4% | 1,453 | 103 | 19 |
| Quasi-2D Perovskite films | 636 | 7.9% | 1,709 | 25 | 20 |
| Colloidal CsPbI_3_ NCs | 634 | 7.1% | 1,391 | 33 | 21 |

**Supplementary Table 2.** Comparison of the average peak EQE from ten devices measured in different laboratories.

| Device | Group | Peak EQE (%)^†^ |
| --- | --- | --- |
| PeLEDs* | Zhengzhou University | 25.8±0.4 |
|  | Cambridge University | 25.2±0.6 |

*The perovskite LEDs (PeLEDs) were fabricated in the same batch at Zhengzhou University. Each set of devices was encapsulated and simultaneously measured until Cambridge group received the samples after 7 days.

^†^The average peak EQE and standard deviation are from ten devices.

**Supplementary Note 1:**

**Surface passivation of CF3-BSA**

We performed Fourier transform infrared spectroscopy (FTIR) and X-ray photoelectron spectroscopy (XPS) to investigate the role of CF3-BSA molecule in the formation of perovskite films. Compared to the control films, XPS spectroscopy of the CF3-BSA based films reveal peaks at 168.6 and 169.8 eV corresponding to S 2*p*_3/2_ and S 2*p*_1/2_, respectively (Supplementary Fig. 15a), and an additional peak at 688.5 eV corresponding to the F 1*s* can be observed, (Supplementary Fig. 15b), confirming the presence of CF3-BSA molecule in the resulting CF3-BSA modified perovskite films. The FTIR spectroscopy results also illustrate above conclusion because of the additional stretching vibration peaks of S=O and C-F in the CF3-BSA based films (Supplementary Fig. 15c). Moreover, in the high-resolution FTIR spectra, we observed that the stretching vibration peak of the S=O group of the CF3-BSA molecule shifted from 1,022.8 cm^-1^ to 1,020.2 cm^-1^ in the CF3-BSA based films (Supplementary Fig. 15d). XPS results further unveil that the O 1*s* signal of CF3-BSA molecule shifts to higher binding energy in the CF3-BSA based films (Supplementary Fig. 15e), whereas the Pb 4*f* signals shift to lower binding energies compared to the control sample (Supplementary Fig. 15f). These results indicate that the oxygen atoms in the S=O can donate their lone electron pairs to the empty 6*p* orbital of Pb^2+^ cations^22^. In addition, two satellite peaks assigned to metallic Pb^0^ near the Pb 4*f* signal at 140.2 and 135.4 eV in the control films can be observed, which is caused by the unsaturated Pb^2+^ at the surface showing metallic characteristics^23^. By contrast, no metallic Pb^0^ signals appear in the CF3-BSA based films, suggesting that the unsaturated Pb^2+^ related defects were effectively passivated by the CF3-BSA molecule.

We then compared the FTIR spectroscopy of neat CF3-BSA molecule, control and CF3-BSA modified perovskite films to verify the interaction between CF3-BSA and POEA^+^ in the perovskite films (Supplementary Fig. 15g). Compared to the control films, the scissoring vibration of ammonium δ(NH_3_^+^) became weakened and shifted to lower wavenumber in the CF3-BSA based films. Meanwhile, compared to the neat CF3-BSA molecule, the peaks corresponding to stretching vibration (C-F) shifted to lower wavenumber in the CF3-BSA based films. These results suggest that the fluorine-derived hydrogen bonds (C-F···H-N) persisted in the CF3-BSA based films. Moreover, the scissoring vibration of amino group δ(NH_2_) was significantly broadened in the CF3-BSA based films (Supplementary Fig. 15d). Meanwhile, the high-resolution XPS spectra of Br 3*d* in CF3-BSA based films show that the core-level spectrum of Br^-^ is slightly shifted to the higher binding energy, and the same trend is observed in core-level spectrum of I^-^ (Supplementary Figs. 15h,i). The shifting trend demonstrates a charge transfer between halides and the active amide hydrogen, indicating the formation of hydrogen bond (N-H···I) between amide hydrogen and iodine. Immobilizing halide ions by hydrogen bonding interactions in perovskite contributes to suppressing halide separation and then stabilizing electroluminescence (EL) color of the mixed-halide PeLEDs^16,24^. The formation of hydrogen bonds between CF3-BSA and perovskite can also be confirmed by the solid-state ^1^H and ^19^F NMR studies. Compared with the neat CF3-BSA molecule, the NMR peaks of ^1^H and ^19^F were split and shifted, suggesting a variation in the chemical environment due to the formation of hydrogen bonds, as demonstrated in Supplementary Fig. 16.

To conduct a quantitative analysis of trap state densities in the perovskite films, we fabricated hole-only devices based on control and CF3-BSA films. These devices were utilized to validate the passivation impact of CF3-BSA on the perovskite and assess the film's transport characteristics through the space-charge-limited current (SCLC) method^25,26^. According to the slope (*k*) of the current-voltage (*I*-*V*) characteristics of the single-carrier devices on a logarithmic scale, three regions can be classified: low-injection regime is the Ohmic region with *k* = 1; a trap-filled limit (TFL) region with *k* > 3, and an SCLC region with *k* = 2 (Supplementary Fig. 17). The hole-only *I*-*V* characteristics show that CF3-BSA based films exhibit lower TFL voltage compared with the control films. We compared the trap state densities of two samples with the equation below:

$n_{\text{t}}\text{ = }\frac{2\varepsilon\varepsilon_{0}V_{\text{TFL}}}{eL^{2}}$ (1)

where *n*_t_ is the trap state density, *V*_TFL_ is the trap-filled limit voltage of hole-only devices, *L* is the thickness of the perovskite, *e* is the elementary charge, and *ε* and *ε*_0_ are relative permittivity and vacuum permittivity, respectively. The trap state density of CF3-BSA based device is calculated to be 4.53×10^17^ cm^-3^, which is half of that (8.95×10^17^ cm^-3^) for the control device. This result provides evidence that the passivation effect of CF3-BSA molecules on nanocrystals introduced in quasi-2D perovskite films contributes to the enhanced luminescence efficiency.

We also carried out thermal admittance spectroscopy to compare the trap-state profiles of the perovskite films. In comparison with the control perovskite, the perovskite film treated with CF3-BSA shows a less deep distribution of defects and a lower defect density (Supplementary Fig. 18). Moreover, density-functional theory (DFT) calculations show that the adsorption energy (*E*_ads_) of S=O group with perovskites is much higher than that of -NH_2_ groups, further confirming that the S=O group of acylamino can coordinate with the uncoordinated Pb^2+^ ions of perovskites preferentially. In addition, DFT calculations show a trap-free bandgap in perovskites with CF3-BSA passivation and hydrogen bonding (N-H···I) between the amide hydrogen and iodine (Supplementary Figs. 19a-e). Furthermore, the time-dependent fluctuations in the I-Pb-I bond angles of the control sample were significantly larger than those of the CF3-BSA based sample, indicating a more stable inorganic octahedral framework in the presence of CF3-BSA (Supplementary Fig. 19f-h).

The change in electrostatic interaction of [PbX_6_]^4-^ can also modify the electronic properties of the perovskite films. We then performed ultraviolet photoelectron spectroscopy (UPS) to analyze the structures and electronic properties of perovskites at the bottom surface of the perovskite films (Supplementary Fig. 20). The results indicate that CF3-BSA based perovskite films exhibit a smaller energy barrier with the hole transport layer, facilitating hole carrier transport. This leads to balanced charge injection in the PeLEDs, which is advantageous for fabricating high-performance devices that achieve both high luminance and high EQE^27^.

**Supplementary Note 2:**

We sought to experimentally identify the interaction between CF3-BSA and perovskite precursors through solution nuclear magnetic resonance (NMR). We first dissolved CF3-BSA in deuterated *N*,*N*-Dimethylformamide (DMF-*d*_7_), then added each perovskite precursors (POEAI or PbI_2_). We observed a resonance signal with a chemical shift at ~7.63 parts per million (ppm), corresponding to the active amide hydrogen. We then added POEAI to the CF3-BSA solution and noted that the signal was broadened and shifted to higher field, indicating that a N-H···I hydrogen bond was formed (Supplementary Fig. 23a). This is because of the electro-withdraw character of iodide that leads to the electron cloud moving to amide hydrogen, then increasing the electron shielding effect. We added PbI_2_ to the CF3-BSA solution and noted that the resonance signal of amide hydrogen shifted to lower field compared with POEAI added into the solution, suggesting that the electron shielding effect becomes weaker. This indicates that in addition to the hydrogen bonding between CF3-BSA and I^-^, there is also an interaction between CF3-BSA and Pb^2+^ in the form of S=O···Pb^2+^ coordination bond (Supplementary Fig. 23a). The addition of POEAI to neat CF3-BSA solution led to downfield-shifted ^19^F NMR signal for fluorine of -CF_3_ in CF3-BSA (Supplementary Fig. 23b), and ^1^H NMR signal for hydrogen of NH_3_^+^ in POEA^+^ was also downfield-shifted, consistent with the fluorine atoms forming hydrogen bonds with POEA^+^ (F···H-N) (Supplementary Fig. 23c).

**Supplementary Note 3:**

**A general design principle for selecting CF3-BSA molecule in this work**

Lead halide perovskites, as ionic crystal materials, are highly sensitive to moisture and oxygen in the air, often leading to defects and degradation, which severely impact the performance and long-term stability of PeLEDs. To address these challenges, carbon oxide, and sulfur oxide groups have been widely used to bond with exposed Pb^2+^ ions, reducing defect states in perovskites and thereby suppressing non-radiative recombination pathways in optoelectronic devices. The solubility in precursors and charge carrier mobility of new molecules should be prioritized, as good solubility enhances doping concentration and high charge carrier mobility helps control the electrical properties of perovskites. Additionally, introducing other functional groups into the molecules to achieve dual or multiple passivation effects will be a valuable strategy. To further understand the additives effect on the growth process and optoelectronic properties of quasi-2D perovskites, we measured the morphologies, PLQEs, and EQEs of the perovskites (Supplementary Figs. 33-35).

We initially introduced two additives, acetophenone, which contains a carbonyl (C=O) group, and (methylsulfonyl)benzene, which features a sulfonyl (S=O) group, to passivate defects in perovskites (Supplementary Fig. 33a). These additives were selected due to their potential to interact with the surface defects in the perovskite lattice and improve the overall optoelectronic properties of the material. However, despite their promising chemical structures and potential for defect passivation, the observed increase in the optoelectronic properties was relatively small. This suggests that while these additives may have a modest impact on surface defect passivation, further optimization of the additive concentration or molecular design may be necessary to achieve a more significant improvement in performance.

To enhance the interaction between additives and perovskites, thereby improving passivation and inhibiting halide ion migration, functional groups capable of forming hydrogen bonds with halides, such as hydroxyl and amine groups, were strategically introduced near the passivating groups (Supplementary Fig. 33b). This modification was designed to strengthen the interaction between the additives and the perovskite surface, promoting a more effective passivation process. Notably, the optoelectronic properties of benzenesulfonamide-based perovskites were found to be significantly superior to those of benzenesulfonic acid-based perovskites. This enhancement is likely attributed to the less acidic nature of benzenesulfonamide compared to benzenesulfonic acid, as the latter's strong acidity can disrupt the delicate surface chemistry of perovskite grains. Such disruption may lead to an increase in grain boundary defects (Supplementary Fig. 33b), which can compromise the material's performance and stability. Therefore, selecting additives with a milder acidity appears to be critical for maintaining the integrity of perovskite films while improving their optoelectronic properties.

To control the diffusion of organic cations during perovskite film deposition, novel functional groups capable of forming hydrogen bonds with organic cations were introduced opposite the passivating groups (Supplementary Fig. 33c). These functional groups were carefully selected to regulate the movement of organic cations within the film, ensuring a more controlled deposition process. As a result, the perovskite films exhibited improved structural integrity, showing compact, smooth, and uniform surfaces (Supplementary Fig. 34c). This led to enhanced PLQEs of the perovskite films and EQEs for the PeLEDs (Supplementary Fig. 35). Among the various functional groups tested, the fluorine group demonstrated the most significant enhancement in optoelectronic properties. This is likely due to fluorine's ability to form stronger hydrogen bonds with organic cations, effectively hindering their uncontrolled diffusion and ensuring more consistent film formation. The strong interaction between fluorine and the organic cations plays a crucial role in optimizing the performance of the PeLEDs, showcasing the importance of precise chemical design in improving device efficiency.

To facilitate the formation of perovskite nanocrystals, a bulky steric hindrance molecule was introduced during the perovskite film deposition process (Supplementary Fig. 33d). The presence of this bulky molecule effectively slowed down the growth of the perovskite crystals, preventing excessive aggregation and promoting the formation of discrete nanocrystals. This resulted in a well-defined nano-island structure (Supplementary Fig. 34d), where the nanocrystals were separated and evenly distributed across the film surface. The nano-island morphology not only improved the film's structural uniformity but also contributed to enhanced optoelectronic properties by minimizing defects and improving charge transport within the perovskite film. The careful control of crystal growth through the introduction of steric hindrance molecules represents a promising strategy for optimizing perovskite film performance in optoelectronic devices (Supplementary Fig. 35). We also fabricated sky-blue emitting (~478 nm) and green emitting (~518 nm) PeLEDs with EQEs of 23.1% and 30.6%, respectively (Supplementary Fig. 36), both of which exceed those of their control devices. These results highlight the promising potential of the multifunctional CF3-BSA molecule in the fabrication of high-performance PeLEDs.

Based on these principles, CF3-BSA molecules incorporate three functional groups that control the diffusion of organic cations, passivate defects, and suppress halide ion migration, large steric hindrance, and ordered molecular assembly, all of which positively interact with quasi-2D perovskites. As a result, CF3-BSA exhibits the most significant enhancement of optoelectronic properties and stability for quasi-2D perovskites based PeLEDs.

**Supplementary References**

1. Kong, L. *et al.* Fabrication of red-emitting perovskite LEDs by stabilizing their octahedral structure. *Nature* **631**, 73–79 (2024).
2. Feng, Y. *et al.* Nucleophilic reaction‐enabled chloride modification on CsPbI_3_ quantum dots for pure red light‐emitting diodes with efficiency exceeding 26 %. *Angew. Chem. Int. Ed.* **63**, e202318777 (2024).
3. Wei, K. et al. Perovskite heteroepitaxy for high-efficiency and stable pure-red LEDs. *Nature* **638**, 949–956 (2025).
4. Song, Y.-H. et al. Intragrain 3D perovskite heterostructure for high-performance pure-red perovskite LEDs. *Nature* **641**, 352-357 (2025).
5. Zhou, X. *et al.* Synchronously polishing the lead-rich surface and passivating surface defects of CsPb(Br/I)_3_ quantum dots for high-performance pure-red PeLEDs. *Nano Lett.* **24**, 3719–3726 (2024).
6. Zhang, J. *et al.* A multifunctional “halide-equivalent” anion enabling efficient CsPb(Br/I)_3_ nanocrystals pure-red light-emitting diodes with external quantum efficiency exceeding 23%. *Adv. Mater.* **35**, 2209002 (2023).
7. Wang, Y.-K. *et al.* In-situ inorganic ligand replenishment enables bandgap stability in mixed-halide perovskite quantum dot solids. *Adv. Mater.* **34**, 2200854 (2022).
8. Zhang, J. *et al.* Ligand-induced cation-π interactions enable high-efficiency, bright and spectrally stable Rec. 2020 pure-red perovskite light-emitting diodes. *Adv. Mater.* **35**, 2303938 (2023).
9. Song, Y. H. *et al.* Hetero-nucleation induced [111]-oriented mixed halide perovskite for stable pure red light-emitting diodes. *Adv. Mater.* **36**, e2411012 (2024).
10. Wang, Y.-K. *et al.* Long-range order enabled stability in quantum dot light-emitting diodes. *Nature* **629**, 586–591 (2024).
11. Feng, Y. *et al.* p-π conjugated L-type ligand for pure-red perovskite light-emitting diodes. *ACS Energy Lett.* **9**, 1125-1132 (2024).
12. Xie, M. *et al.* Suppressing ion migration of mixed‐halide perovskite quantum dots for high efficiency pure‐red light‐emitting diodes. *Adv. Funct. Mater.* **33**, 2300116 (2023).
13. Duan, H. W. *et al.* Bi‐ligand synergy enables threshold low voltage and bandgap stable pure‐red mix‐halide perovskite LEDs. *Adv. Funct. Mater.* **34**, 2310697 (2024).
14. Xie, M. *et al.* High-efficiency pure-red perovskite quantum-dot light-emitting diodes. *Nano Lett.* **22**, 8266–8273 (2022).
15. Guo, J. *et al.* Entropy-driven strongly confined low-toxicity pure-red perovskite quantum dots for spectrally stable light-emitting diodes. *Nano Lett.* **24**, 417–423 (2024).
16. Fu, X. *et al.* Mixed-halide perovskites with halogen bond induced interlayer locking structure for stable pure-red PeLEDs. *Nano Lett.* **23**, 6465-6473 (2023).
17. Song, Y.-H. *et al.* Planar defect–free pure red perovskite light-emitting diodes via metastable phase crystallization. *Sci. Adv.* **8**, eabq2321 (2022).
18. Shen, X. *et al.* Bright and efficient pure red perovskite nanocrystals light‐emitting devices via in situ modification. *Adv. Funct. Mater.* **32**, 2110048 (2022).
19. Yang, L. *et al.* Pure red light-emitting diodes based on quantum confined quasi-two-dimensional perovskites with cospacer cations. *ACS Energy Lett.* **6**, 2386-2394 (2021).
20. Qing, J. *et al.* Spacer cation alloying in ruddlesden-popper perovskites for efficient red light-emitting diodes with precisely tunable wavelengths. *Adv. Mater.* **33**, e2104381 (2021).
21. Mir, W. J. *et al.* Lecithin capping ligands enable ultrastable perovskite-phase cspbi_3_ quantum dots for Rec. 2020 bright-red light-emitting diodes. *J. Am. Chem. Soc.* **144**, 13302-13310 (2022).
22. Ma, D. *et al.* Distribution control enables efficient reduced-dimensional perovskite LEDs. *Nature* **599**, 594-598 (2021).
23. Zhang, W. *et al.* Enhanced optoelectronic quality of perovskite thin films with hypophosphorous acid for planar heterojunction solar cells. *Nat. Commun.* **6**, 10030 (2015).
24. Datta, K. *et al.* Light-induced halide segregation in 2D and quasi-2D mixed-halide perovskites. *ACS Energy Lett* **8**, 1662-1670 (2023).
25. Jeong, J. *et al.* Pseudo-halide anion engineering for α-FAPbI_3_ perovskite solar cells. *Nature* **592**, 381-385 (2021).
26. Ren, H. *et al.* Efficient and stable Ruddlesden–Popper perovskite solar cell with tailored interlayer molecular interaction. *Nat. Photonics* **14**, 154-163 (2020).
27. Kim, J. S. *et al.* Ultra-bright, efficient and stable perovskite light-emitting diodes. *Nature* **611**, 688–694 (2022).
