# Supplementary material for: High-performance red light-emitting diodes from quasi-two-dimensional perovskite nanocrystals
Source: Nat Commun. 2026 May 7;17:6175. doi: 10.1038/s41467-026-72733-w (PMC13365440; doi:10.1038/s41467-026-72733-w)
Supplement: Supplementary file 2 — Description of Additional Supplementary Files [file 41467_2026_72733_MOESM2_ESM.pdf]

Description of Additional Supplementary Files

Supplementary Data 1 | Atomic coordinates of molecular interaction models.
Supplementary Data 1 contains the atomic coordinates of the optimized molecular interaction models used in the theoretical calculations. This dataset includes the optimized geometries of different molecular pairs and precursor complexes, including BSA&BSA, CF3-BSA&CF3-BSA, CF3-BSA&I^-, CF3-BSA&PbI_2, CF3-BSA&POEA, F-BSA&F-BSA and M-BSA&M-BSA. These structural models were used to evaluate the intermolecular interactions and coordination configurations between the sulfonamide based molecules and relevant species involved in the perovskite precursor system. The coordinates provide the optimized atomic positions for each interaction model and support the theoretical analysis of molecular self-assembly, ion binding, and additive component interactions discussed in the main text (Fig. 4a and Fig. 5a).
