# Supplementary material for: High-performance red light-emitting diodes from quasi-two-dimensional perovskite nanocrystals
Source: Nat Commun. 2026 May 7;17:6175. doi: 10.1038/s41467-026-72733-w (PMC13365440; doi:10.1038/s41467-026-72733-w)
Supplement: Supplementary file 4 — Transparent Peer Review file [file 41467_2026_72733_MOESM4_ESM.pdf]

nature portfolio

Peer Review File

High-performance red light-emitting diodes from quasi-two-dimensional perovskite nanocrystals

Corresponding Author: Professor Zhifeng Shi

Parts of this Peer Review File have been redacted as indicated to remove third-party material.

This file contains all editorial decision letters in order by version, followed by all author rebuttals in order by version.

Version 0:

Reviewer comments:

Reviewer #1

(Remarks to the Author)
This manuscript presents a molecular ligand, 4-(trifluoromethyl)benzenesulfonamide (CF3-BSA), as a strategy to regulate the growth of quasi-2D perovskite nanocrystals. Through multifunctional interactions with various components in the perovskite precursor solution, this molecule modulates the optoelectronic properties, crystallization processes, and morphological characteristics of the resulting films. The CF3-BSA treatment demonstrates effectiveness in phase distribution regulation and defect passivation, leading to comprehensive improvements in device performance, including luminance, external quantum efficiency (EQE), and operational stability. However, this study fails to propose innovative mechanisms or fundamental principles, and both its originality and novelty appear insufficient to meet the publication standards of Nature Communications. Therefore, I do not recommend this manuscript for publication in Nature Communications. Specific comments are as follows:

1.The molecule employed in this study, along with its structural analogs, has been extensively utilized and investigated in the performance optimization of perovskite solar cells and PeLEDs (see, for example: Nature, 2023, 615, 830; Adv. Mater., 2024, 36, 2400347; ACS Energy Lett., 2024, 9, 1125; ACS Appl. Mater. Interfaces, 2024, 16, 9012; Angew. Chem. Int. Ed., 2025, 64, e202415669; Adv. Mater., 2024, 36, 2410464). The ability of specific functional groups, most notably sulfonamide, to passivate Pb^{2+} defects has been thoroughly demonstrated, and the associated interaction mechanisms are well understood. Furthermore, ligand engineering strategies based on hydrogen bonding interactions have also been widely reported (Nano Lett., 2023, 23, 11082; Angew. Chem. Int. Ed., 2025, 64, e202419746; Adv. Mater., 2023, 2301624). Within this context, the mechanistic interpretation centered on defect passivation and phase distribution regulation presented in this work does not introduce a new conceptual framework or strategy but essentially applies existing principles. To highlight the innovativeness and significance of this research, the authors should more clearly articulate their unique contributions and provide more systematic characterization data to substantiate the substantial advancement beyond the current state-of-the-art.

2.A noticeable and questionable intensity signal is observed in the 550-600 nm spectral range in Supplementary Figure 9d. The authors should clarify the origin of this anomalous signal and provide evidence demonstrating that it does not affect the accuracy of the reported EQE values.

3.The authors used NMR spectroscopy to verify hydrogen bonding interactions between the CF3-BSA molecule and precursor components (Supplementary Figure 22). However, hydrogen bond formation typically causes a downfield chemical shift (increase in δ value). The observed shift trend in this study contradicts this established rule, and the authors must explain and account for this discrepancy.

4.The manuscript states that device performance data were cross-verified at Zhengzhou University and Cambridge University to ensure reliability (referring to Supplementary Table S2), but this table is missing from the Supplementary Information and should be provided.

5.The operational lifetime of the devices was tested at different luminance levels. While the observed luminance difference spans several orders of magnitude, the corresponding change in lifetime is only minimal. The authors should discuss and

explain the physical mechanism behind this phenomenon.

6.UPS measurements were performed on both the bottom and top surfaces of the perovskite film (Supplementary Figure 19). For ultrathin films, isolating and detecting the bottom interface is particularly challenging. The authors should describe in detail the specific operational procedures used to achieve this measurement in the Experimental Section.

7.Based on Supplementary Figure 21, the authors calculated interaction energies but only listed the final results in the text. To enhance research transparency and facilitate verification, it is recommended to provide relevant computational data (e.g., single-point energies of optimized structures, complex energies) in tabular form within the Supplementary Information.

8.The analysis of in-situ spectroscopic monitoring during film crystallization is insufficient. Although the authors note that the PL peak redshift to ~635 nm is prolonged in CF3-BSA-modified films, this kinetic delay is not intuitively captured in the provided spectra. It is necessary to provide explicit curves quantifying the temporal evolution of the peak position. Furthermore, presenting the corresponding evolution of the integrated PL intensity would further substantiate the effectiveness of this strategy in synergistically achieving passivation and phase distribution regulation.

9.In addition to small-area devices, the authors successfully fabricated large-area devices (1 cm2 and 12.25 cm2) but only provided preliminary film uniformity characterization. A comparative statistical analysis of device performance uniformity across different active areas (e.g., distributions of key parameters like EQE, turn-on voltage, luminance) is currently lacking. Moreover, in-depth discussion on potential performance variations upon scaling and their underlying mechanisms should be included to robustly demonstrate the scalability of this technical approach.

10.Line 154 contains a redundant phrase "the small small-area", which should be corrected to "the small-area".

11.The authors attribute the improved device operational stability to the suppression of ion migration by CF3-BSA, providing the increased activation energy as evidence. However, the fundamental physical origin of this elevated energy barrier remains unelucidated. To strengthen this argument, a more profound analysis of the underlying mechanism is necessary, specifically explaining how the incorporation of CF3-BSA physically raises the energy barrier that ion migration must overcome.

Reviewer #2

(Remarks to the Author)
After reading the article and the authors' response to the other reviewers, I find myself initially aligned with the points raised by Reviewer 1: the central limitation of this manuscript is the absence of new fundamental physical or chemical mechanistic insight. In essence, the work falls into a common category within the perovskite optoelectronics literature, demonstrating a reasonable cause-optimization-effect relationship, but offering limited advance in underlying technical approaches or new materials/device understanding.

That said, I also must acknowledge that the study itself is very systematic, and the authors have responded to the reviewer's comments well. The measurements, analyses, and data interpretation involved also contain no technical issues. Regarding the importance of the study, the major advance is the device performance (rather than conceptual novelty), which is generally considered as a secondary criterion for publication in high-profile journals.

Ultimately, I chose to give credit to the substantial effort behind the meticulous engineering that enabled the record device performance demonstrated in this work, and therefore I recommend the manuscript for publications in Nat Commun. As a peer in the field, I am more looking forward to seeing future studies that advances both the scientific understanding and the engineering aspects of this research area.

Reviewer #3

(Remarks to the Author)
In this work, the authors propose a rational and generalizable molecular design strategy for multifunctional additives, addressing long-standing challenges in defect suppression and ionic regulation within quasi-2D perovskites. The introduction of CF3-BSA, which simultaneously controls organic-cation diffusion, passivates defects, and suppresses halide migration, represents a significant step forward in molecular engineering for perovskite emitters. Moreover, the demonstration of quasi-2D perovskite nanocrystals provides a new materials platform that combines quantum-well energy funneling with nanocrystal-enabled carrier confinement and enhanced light out-coupling, offering fundamentally new opportunities for optoelectronic device design. Building upon these advances, the authors achieve record-high efficiencies in both pure-red and deep-red PeLEDs, including high-performance large-area devices, demonstrating the robustness and broad applicability of their strategy. The depth of mechanistic understanding, supported by extensive experimental evidences, together with the clear technological impact of the device results, positions this study as a strong contribution of broad interest to the materials and optoelectronics communities, fully aligned with the standards of Nature Communications.

Version 1:

Reviewer comments:

Reviewer #3

(Remarks to the Author)
I have carefully checked the response letter and revised manuscript. The comments and points raised by the reviewers have been addressed. The intention of this study is not merely to introduce another passivating molecule, but to provide a deeper understanding of how molecular characteristics influence the crystallization of quasi-2D perovskites from continuous polycrystalline films to nanocrystals. The originality and novelty have been clearly illustrated. I am satisfied with their response and revision. Therefore, I would recommend publication of this manuscript at current version.

Open Access This Peer Review File is licensed under a Creative Commons Attribution 4.0 International License, which permits use, sharing, adaptation, distribution and reproduction in any medium or format, as long as you give appropriate credit to the original author(s) and the source, provide a link to the Creative Commons license, and indicate if changes were made.
In cases where reviewers are anonymous, credit should be given to 'Anonymous Referee' and the source.
The images or other third party material in this Peer Review File are included in the article's Creative Commons license, unless indicated otherwise in a credit line to the material. If material is not included in the article's Creative Commons license and your intended use is not permitted by statutory regulation or exceeds the permitted use, you will need to obtain permission directly from the copyright holder.
To view a copy of this license, visit https://creativecommons.org/licenses/by/4.0/

Reviewer #1 (Remarks to the Author):

This manuscript presents a molecular ligand, 4-(trifluoromethyl)benzenesulfonamide (CF3-BSA), as a strategy to regulate the growth of quasi-2D perovskite nanocrystals. Through multifunctional interactions with various components in the perovskite precursor solution, this molecule modulates the optoelectronic properties, crystallization processes, and morphological characteristics of the resulting films. The CF3-BSA treatment demonstrates effectiveness in phase distribution regulation and defect passivation, leading to comprehensive improvements in device performance, including luminance, external quantum efficiency (EQE), and operational stability. However, this study fails to propose innovative mechanisms or fundamental principles, and both its originality and novelty appear insufficient to meet the publication standards of Nature Communications. Therefore, I do not recommend this manuscript for publication in Nature Communications. Specific comments are as follows:

Our response to Reviewer#1: We thank the reviewer for the thoughtful evaluation of the originality and conceptual significance of this work. We fully agree that sulfonamide based molecules and hydrogen bond mediated ligand engineering have been widely explored for defect passivation in perovskite optoelectronics.

However, the intention of this study is not merely to introduce another passivating molecule, but to provide a deeper understanding of how molecular characteristics influence the crystallization of quasi-2D perovskites from continuous polycrystalline films to nanocrystals. Through systematic comparison of structurally analogous additives, our results suggest that the **combination of large steric hindrance and ordered molecular assembly** plays an important role in regulating crystallization pathways of quasi-2D perovskites. Rather than acting solely as defect passivators, such additives can influence nucleation dynamics, phase distribution, and mesoscale morphology, thereby linking molecular structure to device relevant photophysical processes.

We appreciate the reviewer’s comment and have revised the manuscript to more clearly articulate this perspective and to better position our contribution within the broader context of additive engineering strategies.

R1Q1. The molecule employed in this study, along with its structural analogs, has been extensively utilized and investigated in the performance optimization of perovskite solar cells and PeLEDs (see, for example: Nature, 2023, 615, 830; Adv. Mater., 2024, 36, 2400347; ACS Energy Lett., 2024, 9, 1125; ACS Appl. Mater. Interfaces, 2024, 16, 9012; Angew. Chem. Int. Ed., 2025, 64, e202415669; Adv. Mater., 2024, 36, 2410464). The ability of specific functional groups, most notably sulfonamide, to passivate Pb^{2+} defects has been thoroughly demonstrated, and the associated interaction mechanisms are well understood. Furthermore, ligand engineering strategies based on hydrogen bonding interactions have also been widely reported (Nano Lett., 2023, 23, 11082; Angew. Chem. Int. Ed., 2025, 64, e202419746; Adv. Mater., 2023, 2301624). Within this context, the mechanistic interpretation centered on defect passivation and phase distribution regulation presented in this work does not introduce a new conceptual framework or strategy but essentially applies existing principles. To highlight the innovativeness and significance of this research, the authors should more clearly articulate their unique contributions and provide more systematic characterization data to substantiate the substantial advancement beyond the current state-of-the-art.

Reply to R1Q1: We thank the reviewer for the thoughtful assessment and for highlighting the extensive prior work on sulfonamide based molecules and hydrogen-bond mediated ligand engineering in perovskite optoelectronics. We fully agree that these functional groups have been widely explored for defect passivation, and that their interaction mechanisms are well established.

However, we respectfully clarify that the conceptual advance of this work does not lie in introducing another sulfonamide-based passivating molecule. Rather, our central contribution is the identification of a previously underrecognized molecular design principle governing quasi-2D perovskite nanocrystals. Notably, quasi-2D perovskite nanocrystals remain rarely reported, as most perovskite nanocrystals predominantly adopt a three-dimensional crystal structure.

Specifically, we demonstrate that **the cooperative interplay between large steric hindrance and ordered molecular assembly** of additives plays a decisive role in

regulating crystallization of quasi-2D perovskites from continuous polycrystalline films to nanocrystals. While prior studies have largely focused on isolated chemical interactions (e.g., coordination or hydrogen bonding), our results reveal that supramolecular organization of additives can propagate across length scales and fundamentally reshape the growth and energetic landscape of quasi-2D perovskites.

To substantiate this principle, we systematically investigated a series of structurally analogous sulfonamide-based additives with deliberately tuned dipole moments and steric profiles. Among them, CF3-BSA uniquely integrates pronounced steric bulk with a strong propensity for ordered intermolecular assembly mediated by hydrogen bonding and π–π interactions. Interaction-region indicator (IRI) analysis confirms the formation of well-defined supramolecular networks, whereas additives lacking either sufficient steric volume or assembly capability exhibit disordered interactions (Fig. R1a).

We also quantified the interactions between the additives and the organic spacer cation ($POEA^+$). CF3-BSA exhibits the weakest interaction energy between the sulfonamide group and $POEA^+$ (Fig. R1b), indicating that the sulfonamide moiety remains more accessible for defect passivation through coordination with Pb2+ ions. In contrast, stronger sulfonamide-cation interactions in BSA and M-BSA diminish their passivation efficiency. Meanwhile, CF3-BSA shows the strongest hydrogen-bonding interactions through its fluorine groups with organic cations, which effectively regulate spacer-cation diffusion and suppress the formation of low-n phases (Fig. R1c), as evidenced by the reduced n = 2 domain emission (~540 nm).

Importantly, this molecular ordering is not merely structural but functionally consequential. GIWAXS measurements reveal improved crystallographic orientation (Fig. R2), and SEM imaging confirms a transition from continuous polycrystalline films to nanocrystal architectures (Fig. R1d). These findings indicate that ordered additive assembly can actively modulate nucleation and growth kinetics rather than simply passivating static defects.

From a device-physics perspective, such nanocrystal morphologies suppress interfacial quenching, promote balanced carrier injection, and enhance exciton radiative

recombination. Thus, CF3-BSA acts as a nanoscale structural regulator that simultaneously governs crystallization thermodynamics, phase distribution, and recombination pathways (Fig. R1e).

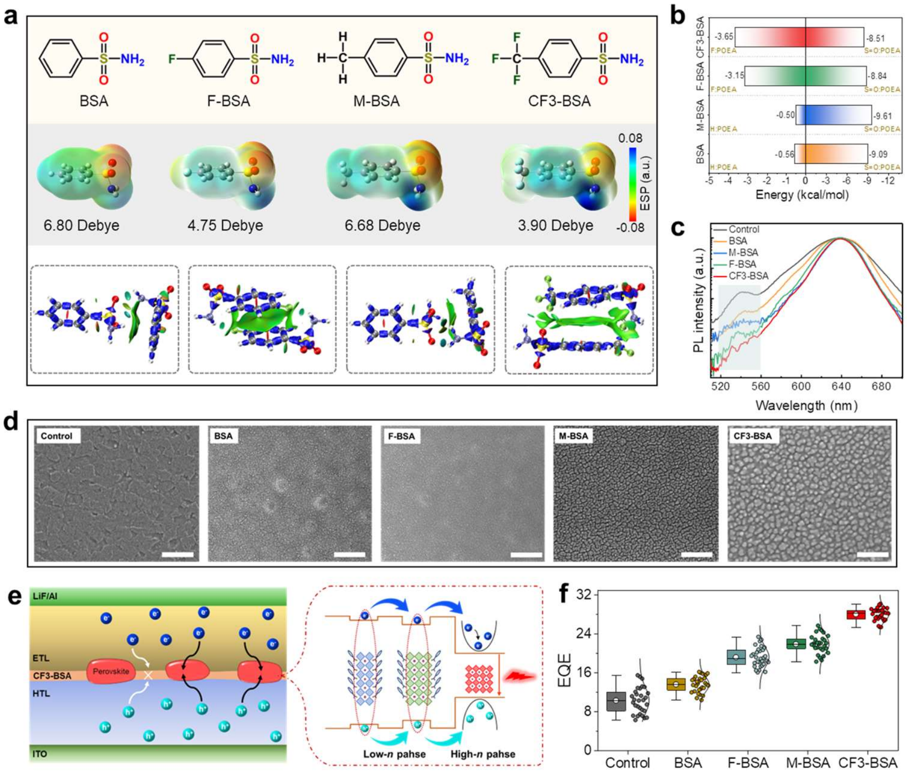

Fig. R1. Cooperative interactions analysis between analogous sulfonamide based additives and quasi-2D perovskites. a, Chemical structures, electrostatic potential (ESP) distributions, and IRI analyses of BSA, F-BSA, M-BSA, and CF3-BSA. **b,** Calculated interaction energies between the additives and the spacer cation $POEA^+$. **c,** PL spectra of the perovskite films incorporated with different additives. **d,** SEM images of the corresponding films; scale bars: 100 nm. **e,** Schematic of the device architecture (left) and cascade energy transfer among different n-phases in quasi-2D perovskites (right). **f,** Statistics of EQEs of the PeLEDs based on different additives.

To further demonstrate the generality of this mechanism, nine additional structurally analogous molecules were evaluated, consistently confirming that only additives possessing both large steric hindrance and ordered assembly capability deliver optimal crystallization behavior and device performance (Fig. R3). We also fabricated sky-blue emitting (~478 nm) and green emitting (~ 518 nm) PeLEDs with EQEs of 23.1% and 30.6%, respectively (Fig. R4), both of which exceed those of their control devices. These results highlight the promising potential of the multifunctional CF3-BSA molecule in the fabrication of high-performance PeLEDs.

Collectively, these findings establish steric–assembly cooperativity as a critical and previously overlooked parameter in additive engineering. This shifts the paradigm from empirical defect passivation toward principle-guided molecular design for controlling crystallization physics in soft ionic semiconductors. This work moves additive engineering beyond conventional defect passivation by identifying steric–assembly cooperativity as a governing molecular parameter for directing crystallization and recombination in quasi-2D perovskites.

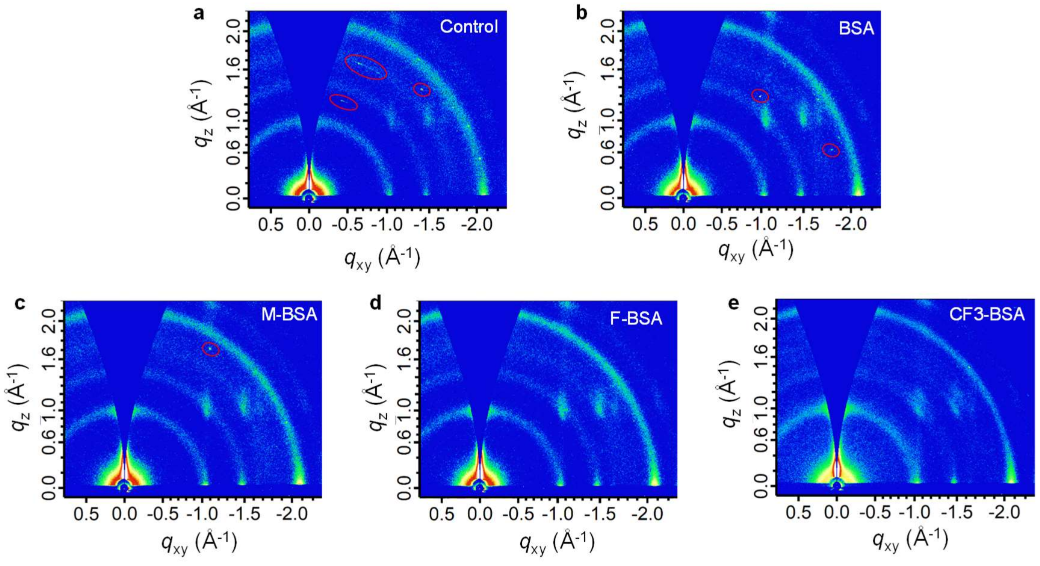

Fig. R2. GIWAX of the perovskite films incorporated with different additives.

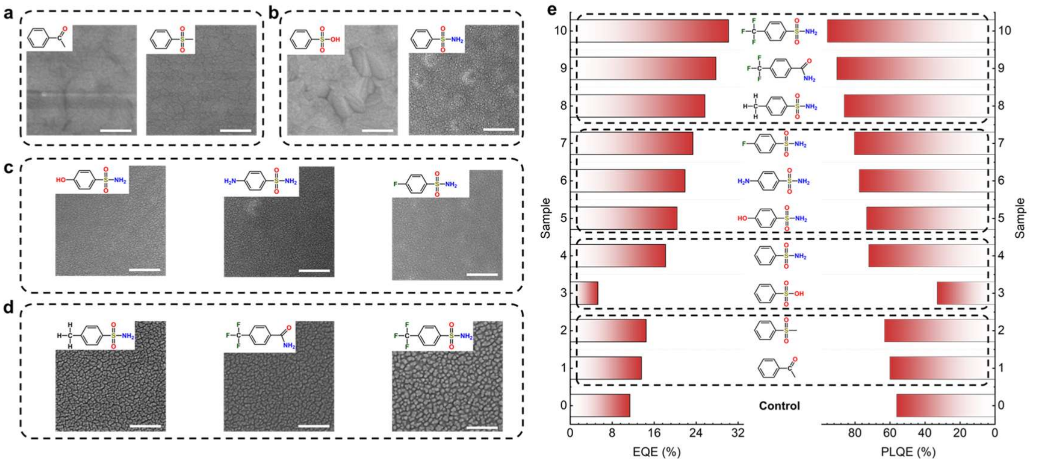

Fig. R3. (a-d) SEM images of the perovskite films based on different additives. (e) PLQEs of perovskite films and corresponding EQEs of devices based on different additives.

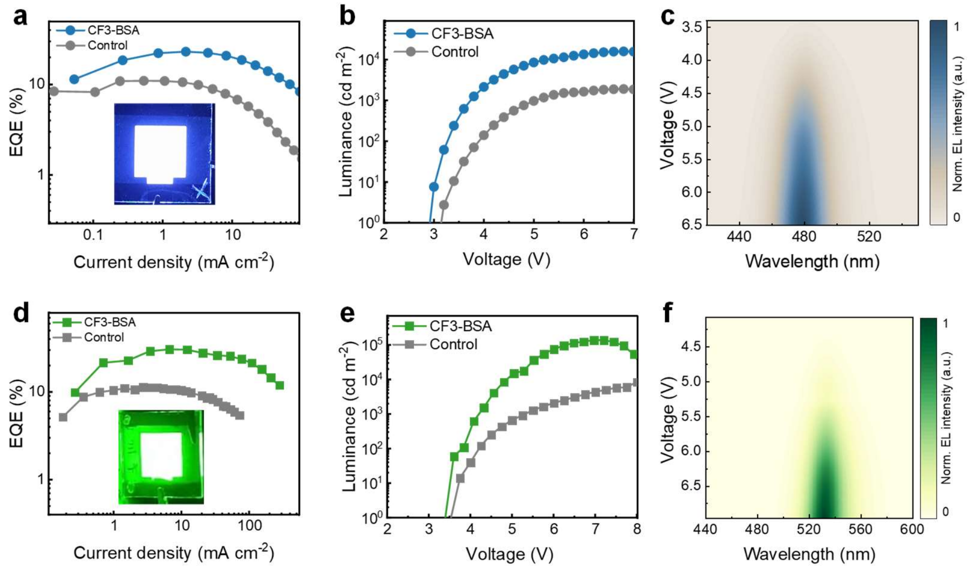

Fig. R4. (a) Current density-EQE curves and (b) voltage-luminance curves of control and CF3-BSA based sky-blue PeLEDs. (c) EL spectra for sky-blue PeLEDs at different operating voltages. (d) Current density-EQE curves and (e) voltage-luminance curves of control and CF3-BSA based green PeLEDs. (f) EL spectra for green PeLEDs at different operating voltages.

Our revision: We have revised the manuscript to more explicitly articulate this conceptual framework and to highlight the structure assembly function relationship uncovered in this study. (Marked red in the revised manuscript: page 15-17)

R1Q2. A noticeable and questionable intensity signal is observed in the 550-600 nm spectral range in Supplementary Figure 9d. The authors should clarify the origin of this anomalous signal and provide evidence demonstrating that it does not affect the accuracy of the reported EQE values.

Reply to R1Q2:

We thank the reviewer for raising this concern regarding the weak intensity signal in the 550–600 nm range. To further clarify its origin and relevance, we have analyzed the EL spectra recorded under a wide range of current densities, as shown in Fig. R5.

As clearly evidenced by the current-density-dependent EL spectra, the weak

feature in the 550–600 nm region is only observable at very low current densities, where the absolute EL intensity of the device is extremely low. With increasing current density, the perovskite emission centered at ~650 nm rapidly intensifies and fully dominates the spectrum, while the 550 – 600 nm signal progressively diminishes and becomes indistinguishable from the baseline at moderate and high current densities, which correspond to the operational regime used for EQE evaluation.

This behavior is inconsistent with intrinsic radiative recombination from perovskite emissive states. Genuine emissive transitions would scale monotonically with injected carrier density and persist across the full current range. Instead, the disappearance of this feature at higher current densities strongly suggests that it originates from measurement-related background contributions, such as weak stray light, detector dark noise, or parasitic optical signals in the EL setup, which become detectable only when the signal-to-noise ratio is low.

From a quantitative perspective, spectral integration analysis shows that the 550–600 nm region contributes only a very small fraction of the total EL intensity (<1%) at low current densities, and its contribution becomes effectively zero at higher current densities where the EL output is dominated by the perovskite emission peak. Importantly, the EQE values reported in this work are extracted from EL spectra measured under steady-state conditions at moderate-to-high current densities, using calibrated detector responsivity and fixed integration boundaries. Therefore, the reported EQE is governed almost entirely by the main emission band and is not influenced by this weak, low-current artifact.

Overall, the current density dependent evolution of the EL spectra provides direct evidence that the 550–600 nm signal is non-intrinsic and does not affect the accuracy or reliability of the reported EQE values.

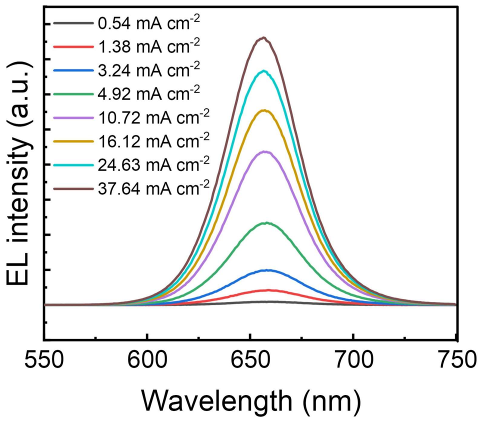

Fig. R5. EL intensity of the device under different current densities.

Our revision: We have replaced Supplementary Fig. 9d with the corrected spectrum and added a clarifying note in the revised manuscript to explicitly address this issue.

R1Q3. The authors used NMR spectroscopy to verify hydrogen bonding interactions between the CF3-BSA molecule and precursor components (Supplementary Figure 22). However, hydrogen bond formation typically causes a downfield chemical shift (increase in δ value). The observed shift trend in this study contradicts this established rule, and the authors must explain and account for this discrepancy.

Reply to R1Q3: We thank the reviewer for this insightful comment. We fully agree that, in most conventional cases, hydrogen-bond formation leads to a downfield shift (increase in δ value) of proton resonances due to deshielding effects, and hydrogen bonding between N–H protons and halide anions typically induces such downfield shifts, as illustrated in Fig. R6a.

However, in the present system, the observed upfield chemical shift can be rationalized by considering the specific electronic environment and competing interactions introduced by the CF3-BSA molecule. CF3-BSA contains a strongly electron-withdrawing sulfonyl group (O=S=O) (Supplementary Fig. 19a), which significantly redistributes the local electron density around the sulfonamide moiety. When the $–NH_2$ group participates in hydrogen bonding with iodide, this interaction occurs simultaneously with pronounced inductive and resonance effects imposed by the

sulfonyl substituent. As a result, hydrogen bonding is accompanied by charge redistribution and enhanced electron density shielding around the N–H protons, rather than simple proton deshielding. The net NMR response therefore reflects a balance between hydrogen-bond induced deshielding and substituent induced shielding effects. In this case, the latter dominates, leading to an overall upfield shift that outweighs the conventional hydrogen-bond downfield trend.

Notably, similar anomalous upfield shifts associated with sulfonamide–halide interactions have been reported previously. Our observations are fully consistent with those reported in [*Adv. Mater.* 2024, 36, 2400347], as shown in Fig. R6b, where comparable NMR shift behavior was observed and attributed to competing electronic effects.

Therefore, the observed chemical shift trend in our NMR data does not contradict hydrogen-bond formation, but rather reflects the complex electronic coupling between hydrogen bonding and strong electron-withdrawing functional groups, providing further evidence for the multifaceted interaction between CF3-BSA and the perovskite precursor components.

[FIGURE REDACTED]

Fig. R6. ^{1}H NMR spectra reproduced from Fig. 2d,e of *Advanced Materials* 2024, 36, 2400347.

R1Q4. The manuscript states that device performance data were cross-verified at Zhengzhou University and Cambridge University to ensure reliability (referring to Supplementary Table S2), but this table is missing from the Supplementary Information

and should be provided.

Reply to R1Q4: Thank you for pointing out this issue. We apologize for the oversight. The referenced Supplementary Table S2, which contains the cross-verified device performance data measured independently at Zhengzhou University and the University of Cambridge, has now been added to the Supplementary Information.

Table R1. Comparison of the average peak EQE from ten devices measured in different laboratories.

Device	Group	Peak EQE (%)†
PeLEDs*	Zhengzhou University	25.8±0.4
	Cambridge University	25.2±0.6

*The perovskite LEDs (PeLEDs) were fabricated in the same batch at Zhengzhou University. Each set of devices was encapsulated and simultaneously measured until Cambridge group received the samples. Which is similar with the method of previous report [Nature 2018, 562, 249-253].

†The average peak EQE and standard deviation are from ten devices.

R1Q5. The operational lifetime of the devices was tested at different luminance levels. While the observed luminance difference spans several orders of magnitude, the corresponding change in lifetime is only minimal. The authors should discuss and explain the physical mechanism behind this phenomenon.

Reply to R1Q5: We thank the reviewer for this insightful comment regarding the weak dependence of device lifetime on luminance over several orders of magnitude.

Although the tested luminance spans multiple orders of magnitude, the corresponding device lifetime exhibits only a modest variation. This behavior can be rationalized by the combined effects of suppressed thermal accumulation and robust carrier injection balance in the CF3-BSA based devices.

Firstly, owing to the high EQE and low operating voltage enabled by CF3-BSA

incorporation, a smaller fraction of the injected electrical power is converted into Joule heating. As shown in Fig. R7, after 50 s of operation at 4 V, the temperature of the control device increases from 23 °C to 45.7 °C, whereas the CF3-BSA based PeLED exhibits a significantly reduced temperature rise, from 23 °C to 31.9 °C. These results indicate that the CF3-BSA based devices operate with improved thermal efficiency. Consequently, the device temperature does not scale linearly with luminance, and thermal accumulation remains moderate even at elevated current densities. Previous studies on high-efficiency PeLEDs have reported similar temperature saturation behavior, attributed to efficient heat dissipation through the substrate and charge-transport layers, resulting in a sublinear relationship between luminance and thermal stress [*Nat. Commun.* 2024, 15, 6561].

Secondly, CF3-BSA effectively suppresses defect-assisted recombination and ion migration, thereby maintaining balanced electron and hole injection over a wide current-density range. This balanced carrier injection minimizes local carrier accumulation and mitigates current-induced degradation pathways, such as Joule-heating induced hotspot formation, which are typically exacerbated at high luminance.

Therefore, the operational stability of the devices is primarily governed by intrinsic ionic and interfacial stability rather than luminance-induced thermal effects, resulting in only minor lifetime variations across different luminance levels.

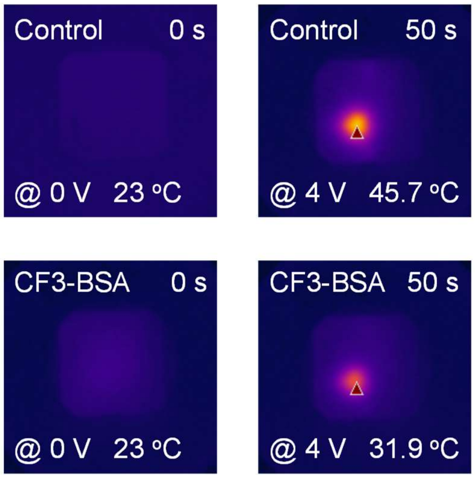

Fig. R7. Infrared-thermal-imaging pictures of the PeLEDs before and after operating at 4 V for 50 s.

R1Q6. UPS measurements were performed on both the bottom and top surfaces of the perovskite film (Supplementary Figure 19). For ultrathin films, isolating and detecting the bottom interface is particularly challenging. The authors should describe in detail the specific operational procedures used to achieve this measurement in the Experimental Section.

Reply to R1Q6: Thank you for this valuable comment. We agree that obtaining UPS spectra from the bottom interface of an ultrathin perovskite film is technically challenging, and we have now included a detailed description of the procedure in the Experimental Section.

In brief, the perovskite film was first spin-coated onto an ITO substrate, and the UPS spectrum of the top surface was collected directly from the as-prepared film. To probe the bottom interface, we then removed approximately 90 s of the perovskite layer using a controlled Ar^+ ion–etching process prior to performing the second UPS measurement. This mild etching exposes the bottom perovskites interface while minimizing potential damage to the electronic structure, enabling reliable characterization of the bottom interface.

Our revision: The perovskite film was first spin-coated onto an ITO substrate, and the UPS spectrum of the top surface was collected directly from the as-prepared film. To probe the bottom interface, we then controlled Ar^+ ion etching process for 90s prior to performing the second UPS measurement. (Revised manuscript, Page 24, Lines 13-17)

R1Q7. Based on Supplementary Figure 21, the authors calculated interaction energies but only listed the final results in the text. To enhance research transparency and facilitate verification, it is recommended to provide relevant computational data (e.g., single-point energies of optimized structures, complex energies) in tabular form within the Supplementary Information.

Reply to R1Q7: Thank you for this constructive suggestion. In the revised Supplementary Information, we have now provided the detailed computational data associated with the interaction-energy calculations shown in Supplementary Figure 21.

Specifically, we include the single-point energies of all optimized molecular structures, the energies of the corresponding molecular complexes (Fig. R8), and the calculated interaction energies in a tabulated format (Tabel R2). These additions enhance the transparency and reproducibility of our computational analysis and allow readers to independently verify the reported interaction-energy values.

Table R2. Single-point energies of the systems between CF3-BSA and perovskite precursors.

	System	*E* / hartree	hartree#	kcal·mol^{-1}
Monomer 1	I^-	-297.9757438		
Monomer 2	CF3-BSA	-1172.57868		
Dimer	CF3-BSA+I^-	-1470.562413		
Interaction Energy (ΔE)			-0.008	-5.013
Monomer 1	PbI_2	-788.6886465		
Monomer 2	CF3-BSA	-1172.57868		
Dimer	CF3-BSA+PbI_2	-1961.296289		
Interaction Energy (ΔE)			-0.029	-18.174
Interaction Energy (ΔE)	CF3-BSA+Pb^{2+}		-0.013	-8.148*
Monomer 1	POEA	-402.338624		
Monomer 2	CF3-BSA	-1172.57868		
Dimer	CF3-BSA+POEA	-1574.92312		
Interaction Energy (ΔE)			-0.006	-3.649

Interaction Energy (ΔE) = $E_{\text{Dimer}} - E_{\text{Monomer 1}} - E_{\text{Monomer 2}}$

* The interaction energy of the CF3-BSA+Pb^{2+} dimer was estimated by subtracting twice the interaction energy of the CF3-BSA+I^- dimer from that of the CF3-BSA+PbI_2 dimer.

\# 1 hartree = 627.51 kcal·mol^{-1}

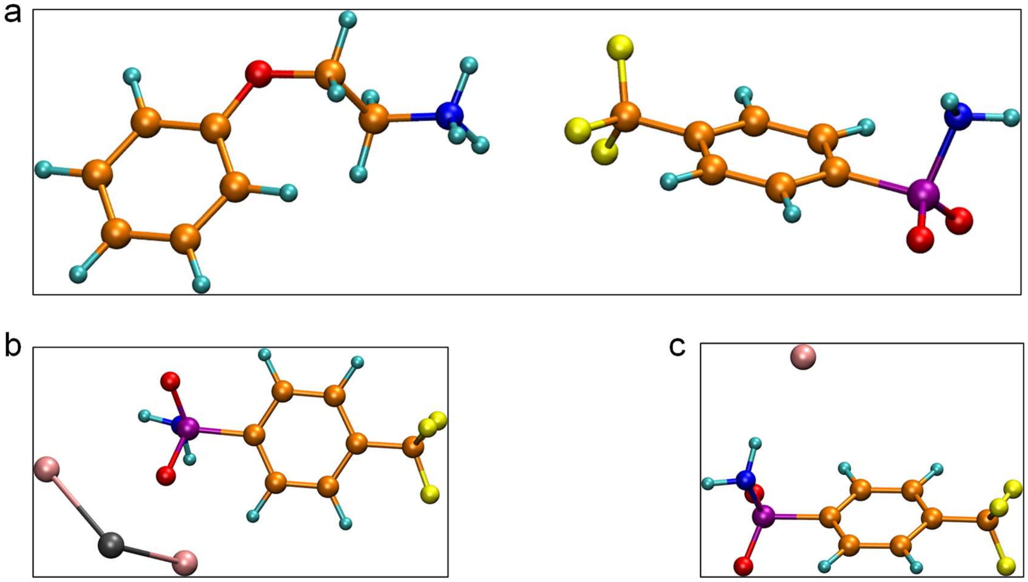

Fig. R8. Optimized structures of the Dimer of (a) CF3-BSA+POEA, (b) CF3-BSA+PbI_2, (c) CF3-BSA+I^-.

R1Q8. The analysis of in-situ spectroscopic monitoring during film crystallization is insufficient. Although the authors note that the PL peak redshift to ~635 nm is prolonged in CF3-BSA-modified films, this kinetic delay is not intuitively captured in the provided spectra. It is necessary to provide explicit curves quantifying the temporal evolution of the peak position. Furthermore, presenting the corresponding evolution of the integrated PL intensity would further substantiate the effectiveness of this strategy in synergistically achieving passivation and phase distribution regulation.

Reply to R1Q9: We thank the reviewer for pointing out the insufficient analysis of the in situ spectroscopic monitoring during film crystallization and apologize for the inaccurate description in the original manuscript.

Based on the in situ PL measurements, the CF3-BSA modified perovskite films reach a stable PL peak at an earlier stage of crystallization than the control sample (Fig. R9a), rather than exhibiting a delayed redshift as previously stated. This correction has been implemented consistently throughout the revised manuscript. The accelerated redshift indicates that CF3-BSA promotes the early formation of larger-*n* quasi-2D phases during the initial crystallization stage. We attribute this behavior to the multivalent coordination of CF3-BSA with perovskite precursors, which regulates

lead–halide octahedra assembly, suppresses disordered nucleation, and facilitates rapid phase evolution toward emissive large-*n* domains.

We further analyzed the time-dependent PL intensity, which provides a direct metric of phase evolution and energetic disorder during film formation. As shown in Fig. R9b, the CF3-BSA modified films exhibit a faster increase in PL intensity, indicative of suppressed phase heterogeneity and more coherent energy funneling during crystallization.

Together, the PL peak kinetics and PL intensity evolution demonstrate that CF3-BSA plays a dual role in accelerating large-*n* phase formation and reducing crystallization disorder, thereby enabling effective defect passivation and optimized phase distribution. These clarifications have been incorporated into the revised manuscript to improve clarity and physical rigor.

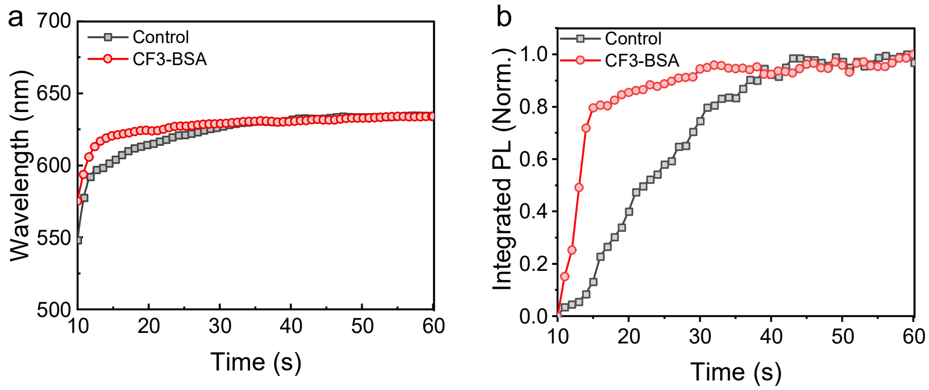

Fig. R9. In situ spectroscopic tracking of perovskite film crystallization. The evolution of (a) PL peak position and (b) normalized PL intensity for the control and CF3-BSA modified films. (Supplementary Fig. 25 in the revised supporting information)

Our revision: However, the CF3-BSA modified perovskite films reach a stable PL peak at an earlier stage of crystallization than the control sample (Supplementary Fig. 25a). Moreover, the PL intensity of the CF3-BSA based films increases more rapidly during crystallization (Supplementary Fig. 25b). The accelerated PL redshift, together with the faster intensity buildup, indicates that CF3-BSA promotes the early formation of larger-*n* quasi-2D phases at the initial crystallization stage. This behavior reflects a more homogeneous phase evolution and demonstrates effective regulation of crystallization

kinetics by CF3-BSA. (Revised manuscript, Page 13 line 7-14)

R1Q9. In addition to small-area devices, the authors successfully fabricated large-area devices (1 cm^2 and 12.25 cm^2) but only provided preliminary film uniformity characterization. A comparative statistical analysis of device performance uniformity across different active areas (e.g., distributions of key parameters like EQE, turn-on voltage, luminance) is currently lacking. Moreover, in-depth discussion on potential performance variations upon scaling and their underlying mechanisms should be included to robustly demonstrate the scalability of this technical approach.

Reply to R1Q9: We thank the reviewer for raising this important point regarding device scaling. Compared with small-area PeLEDs, large-area devices exhibit broader performance distributions and increased nonuniformity (Fig. R10). This behavior primarily arises from intrinsic limitations of the spin-coating process, including spatially nonuniform solvent evaporation, local supersaturation fluctuations, and inhomogeneous nucleation density across extended substrates. These effects lead to spatial variations in the emissive layer, where locally degraded interfacial quality results in shortened PL lifetimes (Fig. R11) and elevated non-radiative recombination rates (Fig. R12), particularly near the edges of the large-area films. Such microscopic inhomogeneities ultimately manifest as dispersions in external quantum efficiency, turn-on voltage, and luminance.

The preserved electroluminescence uniformity over centimetre scale areas indicates that performance degradation upon scaling does not originate from catastrophic morphological failure. Rather, it reflects the gradual accumulation of microscopic disorder that perturbs charge balance and enhances non-radiative recombination pathways. This highlights an inherent physical limitation of spin coated large-area PeLEDs, in which crystallization kinetics become increasingly difficult to synchronize over large lateral dimensions.

The scalability demonstrated here should be regarded as a physically constrained yet robust extension of small-area optimization, rather than a fully scale-invariant solution. The retention of reasonable device performance at the centimetre scale further

confirms that the observed degradation arises from process-induced macroscopic nonuniformities rather than a breakdown of the proposed material or interfacial mechanisms. We note that further improvements in large-area uniformity will require process-level innovations—such as controlled solvent evaporation or scalable coating techniques (for example, blade coating, slot-die coating, or inkjet printing)—which are beyond the scope of the present work.

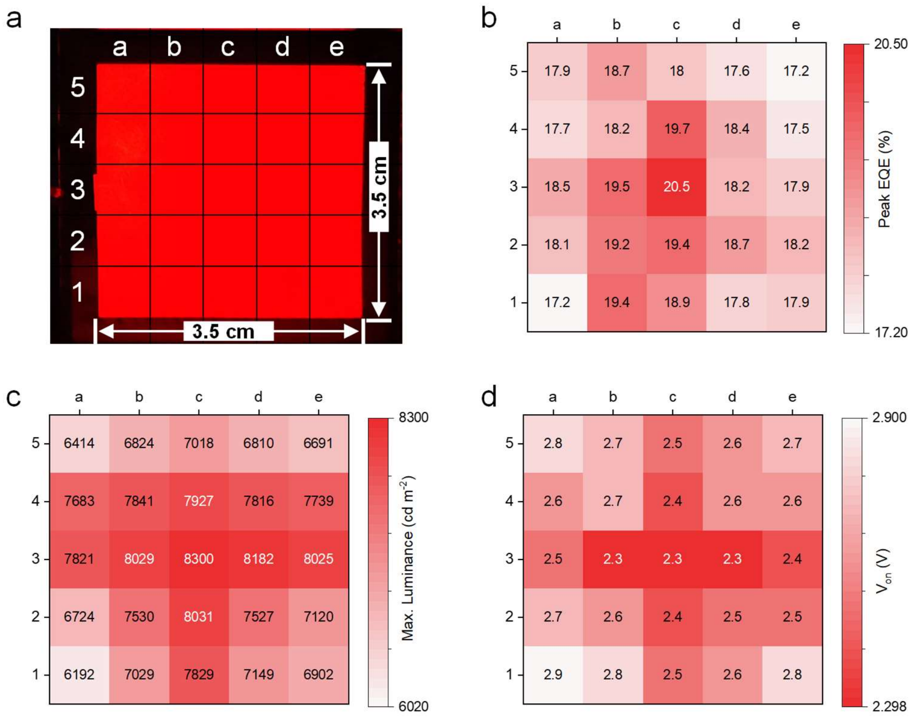

Fig. R10. Performance of different active areas for the large-area PeLED. (a) A working devices with 3.5 cm × 3.5 cm active area. (b) Peak EQEs of different active areas for the device. (c) Maximum luminance of different active areas for the device. (d) Turn-on voltage of different active areas for the device.

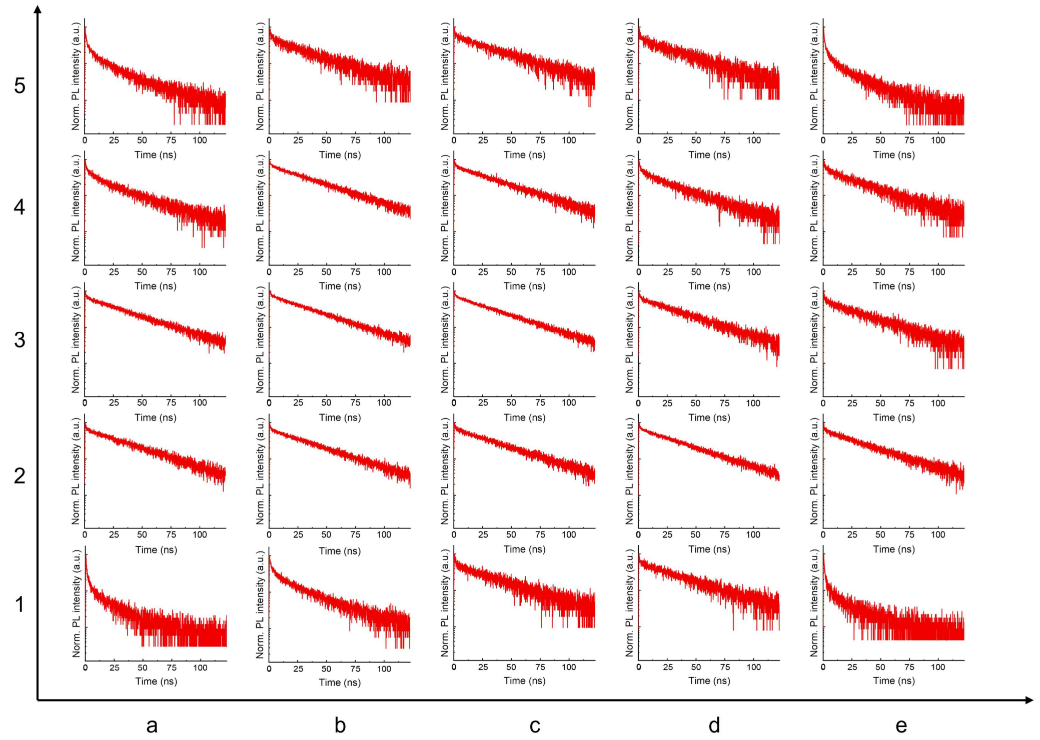

Fig. R 11. TRPL decay curves collected from 25 regions across a 3.5 cm × 3.5 cm active-area film.

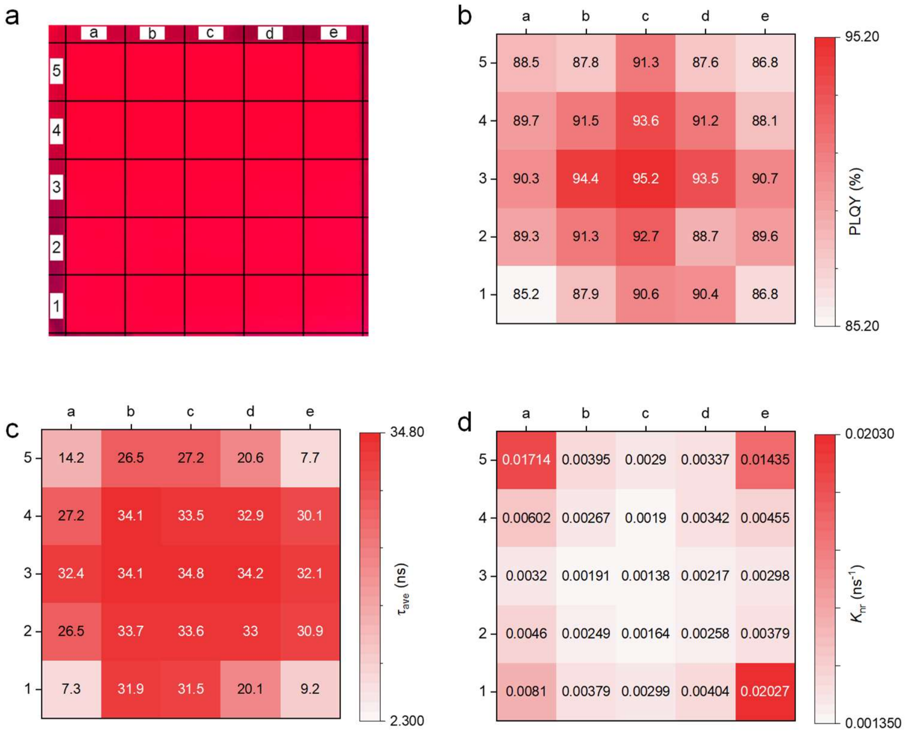

Fig. R12. (a) (a) Photograph of a 3.5 cm × 3.5 cm CF3-BSA perovskite film under 365

nm UV light. (b–d) Statistics of PLQE, PL lifetime, and non-radiative recombination rate.

Note:

The non-radiative recombination rate of perovskites were calculated by adopting photoluminescence quantum yields (PLQY) and the mean lifetimes (τ_{avg}) of the samples. PLQY can be obtained by using formula of PLQY = N_{pe}/N_{pa}, where N_{pe} is number of photons emitted as photoluminescence from sample, N_{pa} is number of photons absorbed by sample. And both of radiative recombination and non-radiative recombination are the excited state. Hence, by evaluating the PLQY of all the samples, the average radiative lifetime and non-radiative recombination rates were also able to be calculated.

$$\mathrm{PLQY} = \frac{K\mathrm{r}}{K\mathrm{nr} + K\mathrm{r}}$$

$$\tau\mathrm{avg} = \frac{1}{K\mathrm{nr} + K\mathrm{r}}$$

Then the non-radiative recombination rate (K_{nr}) can be calculated:

$$\mathrm{Knr} = \frac{1 - PLQY}{\tau\mathrm{avg}}$$

R1Q10. Line 154 contains a redundant phrase "the small small-area", which should be corrected to "the small-area".

Reply to R1Q10: Thank you for pointing out this wording issue. We have carefully revised the manuscript to correct the redundancy. Specifically, the phrase “the small small-area” has been corrected to “the small-area” in the revised manuscript. (Page 6, Lines 20)

R1Q11. The authors attribute the improved device operational stability to the suppression of ion migration by CF3-BSA, providing the increased activation energy as evidence. However, the fundamental physical origin of this elevated energy barrier remains unelucidated. To strengthen this argument, a more profound analysis of the underlying mechanism is necessary, specifically explaining how the incorporation of CF3-BSA physically raises the energy barrier that ion migration must overcome.

Reply to R1Q11: We thank the reviewer for raising this important question regarding

the physical origin of the increased ion-migration activation energy upon CF3-BSA incorporation.

The activation energy extracted from temperature-dependent ionic conductivity measurements reflects the effective energy required for mobile ions to become ionic charge carriers by overcoming the migration barrier, rather than a direct measurement of a single elementary hopping event (Appl. Phys. Lett. 2021, 119, 031902). As established in recent studies, the measured activation energy is jointly governed by two key factors: (i) the formation energy (or population) of mobile ionic defects, such as halide vacancies, and (ii) the intrinsic migration barrier along the diffusion pathway (Nano Lett. 2023, 23, 6465).

Firstly, CF3-BSA effectively passivates halide vacancy related defects, which serve as the dominant migration channels for halide ions in mixed-halide perovskites. By reducing the density of available vacancies, the number of ions that can participate in long-range migration is significantly suppressed. This leads to a reduced ionic conductivity and manifests as an increased apparent activation energy in temperature-dependent measurements.

Secondly, CF3-BSA introduces $-NH_2$ functional groups that form directional hydrogen bonds (H···I) with halide ions. Compared with the relatively weak van der Waals interactions between organic cation and halide ions, these hydrogen-bond interactions provide a stronger and more localized binding environment (Science Bulletin, 2021, 66, 2189). Consequently, additional energy is required for halide ions to break these interactions and migrate between adjacent lattice sites, thereby increasing the intrinsic migration barrier (Nano Lett. 2023, 23, 3385).

To elucidate the underlying mechanism, first-principles calculations were performed to investigate the influence of CF3-BSA on iodide ion migration in $CsPbI_3$ (Fig. R13a). On the pristine $CsPbI_3$ surface, the calculated migration barrier for iodide vacancies is 0.55 eV (Fig. R13b). In contrast, after surface modification with CF3-BSA, the migration barrier increases substantially to 0.79 eV (Fig. R13b), indicating a pronounced suppression of iodide vacancy diffusion. This enhancement originates from the strong electrostatic interaction between CF3-BSA and migrating I^- ions, which

effectively impedes ion transport along the surface diffusion pathway and raises the associated energy barrier (Fig. R13c).

In summary, the elevated activation energy arises from the synergistic effects of reduced mobile defect density and strengthened local halide binding induced by CF3-BSA. These two contributions cooperatively suppress ion migration, thereby accounting for the markedly improved operational stability of the devices.

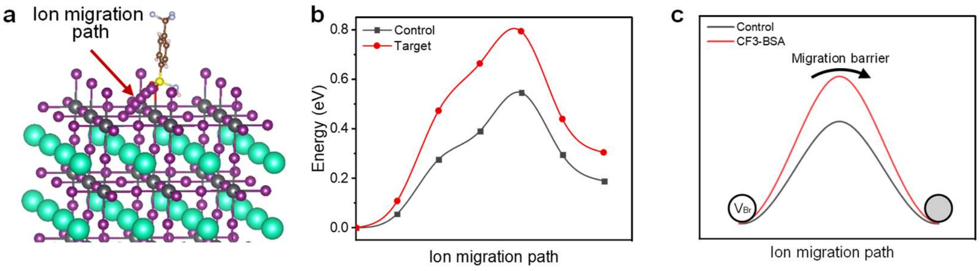

Fig. R13. (a) Illustration of halide ion vacancy migration pathway on the surface of the perovskites. (b) Calculated energy profile along the corresponding ionic migration pathway and (c) schematic of the migration barrier of halide ion vacancies on the surface of perovskites before and after treated by CF3-BSA.

Our revision: We have added a discussion in the revised manuscript to clarify how the incorporation of CF3-BSA physically increases the energy barrier for ion migration. (Page 8 line1 and page 9 line 1-7, Figure 12 in the revised supporting information.)

Reviewer #2 (Remarks to the Author):

After reading the article and the authors' response to the other reviewers, I find myself initially aligned with the points raised by Reviewer 1: the central limitation of this manuscript is the absence of new fundamental physical or chemical mechanistic insight. In essence, the work falls into a common category within the perovskite optoelectronics literature, demonstrating a reasonable cause-optimization-effect relationship, but offering limited advance in underlying technical approaches or new materials/device understanding.

That said, I also must acknowledge that the study itself is very systematic, and the authors have responded to the reviewer's comments well. The measurements, analyses, and data interpretation involved also contain no technical issues. Regarding the importance of the study, the major advance is the device performance (rather than conceptual novelty), which is generally considered as a secondary criterion for publication in high-profile journals.

Ultimately, I chose to give credit to the substantial effort behind the meticulous engineering that enabled the record device performance demonstrated in this work, and therefore I recommend the manuscript for publications in Nat Commun. As a peer in the field, I am more looking forward to seeing future studies that advances both the scientific understanding and the engineering aspects of this research area.

Our response to Reviewer#2: We thank the reviewer for the careful and balanced assessment. While we agree that this work does not introduce an entirely new physical or chemical paradigm, it provides a systematic and technically rigorous study that establishes a clear cause optimization performance relationship in quasi-2D perovskite PeLEDs. According the reviewer comments, we added the the central outcome of this work is the identification of a practical design principle. As shown in the revised manuscript (Marked red in the page 15-17), the combination of large steric hindrance and ordered molecular assembly in additives plays a decisive role in regulating crystallization pathways and carrier recombination in PeLEDs.

Distinct from conventional additives, the multifunctional molecule CF3-BSA simultaneously coordinates with organic spacer cations, Pb^{2+} ions, and halide ions, and

further forms ordered assemblies through hydrogen bonding and π–π stacking. This cooperative regulation drives the formation of quasi-2D perovskite nanocrystal architectures, suppresses defect formation and ion migration, and enhances exciton confinement and radiative recombination, ultimately enabling exceptional device performance. We believe that this engineering-oriented insight provides a broadly applicable strategy for the rational design of high-performance PeLED materials.

We also value the reviewer's perspective and share the aspiration that future studies will continue to advance both fundamental understanding and device engineering in this field.

Reviewer #3 (Remarks to the Author):

In this work, the authors propose a rational and generalizable molecular design strategy for multifunctional additives, addressing long-standing challenges in defect suppression and ionic regulation within quasi-2D perovskites. The introduction of CF3-BSA, which simultaneously controls organic-cation diffusion, passivates defects, and suppresses halide migration, represents a significant step forward in molecular engineering for perovskite emitters. Moreover, the demonstration of quasi-2D perovskite nanocrystals provides a new materials platform that combines quantum-well energy funneling with nanocrystal-enabled carrier confinement and enhanced light out-coupling, offering fundamentally new opportunities for optoelectronic device design. Building upon these advances, the authors achieve record-high efficiencies in both pure-red and deep-red PeLEDs, including high-performance large-area devices, demonstrating the robustness and broad applicability of their strategy. The depth of mechanistic understanding, supported by extensive experimental evidences, together with the clear technological impact of the device results, positions this study as a strong contribution of broad interest to the materials and optoelectronics communities, fully aligned with the standards of Nature Communications.

Our response to Reviewer#3: We sincerely thank the reviewer for this thoughtful and encouraging assessment of our work. We greatly appreciate the recognition that this study moves beyond incremental optimization by establishing a rational and generalizable molecular design strategy for multifunctional additives in quasi-2D perovskites.

As highlighted by the reviewer, the central advance of this work lies in demonstrating that a single molecule of CF3-BSA can simultaneously regulate organic-cation diffusion, passivate surface defects, and suppress halide-ion migration. This unified molecular functionality addresses multiple long-standing challenges in quasi-2D perovskite emitters within a coherent design framework. Importantly, this multifunctionality was systematically validated through comparative studies using structurally analogous molecules, allowing us to extract transferable structure–function relationships rather than relying on an isolated "magic-molecule" effect.

We also appreciate the reviewer's recognition of the quasi-2D perovskite nanocrystal platform, which uniquely integrates quantum-well–driven energy funneling with nanocrystal, enabled carrier confinement and enhanced light out-coupling. We believe this hybrid materials concept opens new opportunities for optoelectronic device engineering beyond conventional quasi-2D thin-film architectures.

We are grateful for the acknowledgment that the combination of in-depth mechanistic analysis and record-level device performance, including scalable large-area PeLEDs, demonstrates the robustness and broad applicability of our strategy. We share the reviewer's view that continued efforts should further advance both fundamental understanding and device engineering, and we hope this study will serve as a useful foundation toward that goal.
